# Probabilistic Isolation of Crystalline Inorganic Phases

**DOI:** 10.1021/acs.jcim.5c02256

**Published:** 2025-12-04

**Authors:** Daniel Ritchie, Michael W. Gaultois, Vladimir V. Gusev, Vitaliy Kurlin, Matthew J. Rosseinsky, Matthew S. Dyer

**Affiliations:** † Leverhulme Research Centre for Functional Materials Design, 151583 Materials Innovation Factory, 51 Oxford Street, Liverpool L7 3NY, U.K.; ‡ Department of Chemistry, 4591 University of Liverpool, Crown Street, Liverpool L69 7ZD, U.K.; § Department of Computer Science, University of Liverpool, Ashton Building, Liverpool L69 3DR, U.K.

## Abstract

We present Probabilistic Isolation of Crystalline Inorganic Phases (PICIP), a tool to accelerate materials discovery by automating the process of isolating unknown crystalline inorganic phases that have been experimentally detected. PICIP can be used by any lab worker, is well suited to both traditional as well as automated high-throughput exploratory workflows, and is a novel approach to isolating unknown phases based on experimental information from sampled compositions. PICIP infers the composition of an unknown phase in a mixed phase sample from the average composition of the sample and the weighted average composition of the known phases in that sample, relying on experimental phase identification and quantification. We implement a novel algorithm that infers the probability density for the unknown phase over a linear representation of compositional phase space. The accuracy of the suggested target compositions can be increased by systematically combining information from different sampled compositions across multiple experiments. This allows for the effective adoption of an iterative sampling strategy that suggests target compositions that converge to the composition of the unknown phase. The linear representation used for compositional phase space can exploit chemical constraints such as charge neutrality to reduce the dimension of the space, while implicitly ensuring only valid compositions are suggested. Simulated exploration of phase fields shows that after four sequential samples, or two batches of five samples, the median purity of the unknown crystalline phase is above 90%. PICIP’s probabilistic construction makes it robust to moderate levels of experimental error in phase quantification (13 wt %), and allows for the identification of scenarios where there are significant levels of experimental error.

## Graphical abstract

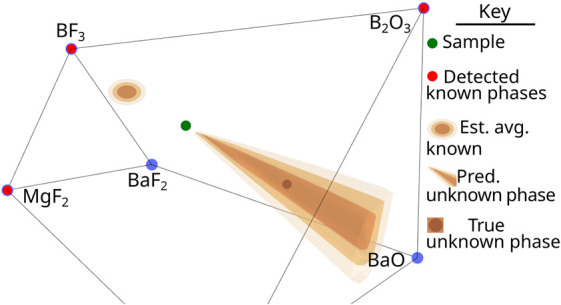

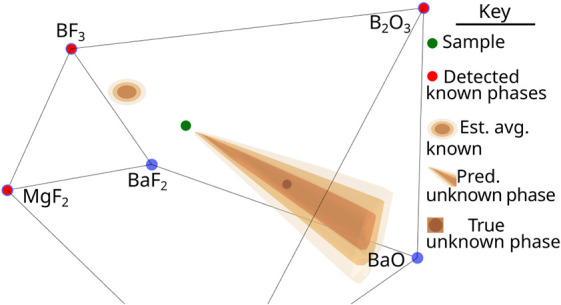

## Introduction

The discovery of novel crystalline phases with desirable properties is one of the aims of materials science and inorganic chemistry. A traditional approach is to choose a phase field consisting of a set of elements, choose different compositions consisting of these elements in different ratios, and then react appropriate starting materials under the required reaction conditions, often at elevated temperature if solid state diffusion is slow. In general, the resulting product from each of these compositions will be a mixture of different crystalline phases. Powder X-ray diffraction (PXRD) analysis of these mixtures can then determine whether any of these mixtures contain a previously undiscovered crystalline phase. If an unknown crystalline phase is detected, the yield is usually too low to accurately determine its structure and properties, so the initial composition must be adjusted to increase the yield. Ideally, the yield would be 100%, which, assuming that the system is closed, requires the sample’s composition to be equal to the composition of the unknown crystalline phase. In practice, reaction kinetics may mean that the composition that results in the highest yield may not exactly be the composition of the unknown crystalline phase. Nevertheless, we use the term “isolating the unknown crystalline phase” as the process of targeting the composition of the unknown crystalline phase with the aim of synthesizing it with a suitably high yield.

The standard approach for varying the composition to increase the yield of an unknown crystalline phase uses ideas based on the lever rule.^1^ The lever rule is a tool used in the analysis of phase diagrams to calculate the relative molar ratios of different crystalline phases formed from a given composition. It is a consequence of the fact that if a composition separates into several crystalline phases, then the relative amounts of each crystalline phase will ensure that their average composition is the same as the initial composition. For a mixture of an unknown crystalline phase and several known crystalline phases, the lever rule cannot be used directly owing to the presence of a phase with unknown composition. However, an estimation of the composition of the unknown phase may be made by inverting the lever rule. Given the overall composition of the sample and the average composition of the detected known phases, the unknown phase must be located in the region of the phase diagram opposite to the average composition of the known phases, from the overall composition's perspective. The average composition of the known phases can be calculated from their relative amounts. When these are known exactly, this restricts the estimated location of the unknown phase to a line through the phase field. This line is referred to as the “estimated direction” (Figure 2c).

In practice the relative amounts of the known phases are calculated from experimental data and have an associated uncertainty that depends on the accuracy of the quantification technique. This translates to an angular error of the estimated direction. As a consequence, the uncertainty on the composition of the unknown phase is proportional to the distance between the sampled composition and the unknown phase, which is determined by the relative molar ratio of the unknown phase. Compositional predictions are therefore less accurate the lower the yield of the unknown phase. In addition, it is difficult to make predictions using information from multiple sampled compositions, especially in higher-dimensional phase fields. Computing the estimated directions and finding their intersection points is not simple even in two dimensions. Further, three or more estimated directions are unlikely to share an intersection due to the presence of experimental error. Accounting for and integrating this experimental error when predicting the composition of the unknown phase is also challenging.

To overcome these difficulties modern approaches employ high-throughput synthesis techniques.^2–10^ These methods enable dense sampling of the phase field, which, when combined with automated methods for phase identification^11^ and composition targeting,^12^ facilitates the isolation of new crystalline phases. However, these methods often require specialized equipment such as synchrotron facilities and bespoke synthetic platforms, limiting accessibility and availability.

We present an algorithm that uses the estimated direction and its associated uncertainty to construct a conical probability distribution for the composition of an unknown phase, where the width of the cone is determined by the amount of uncertainty. The estimated direction is solely calculated from the initial composition, and the weighted average composition of all phases except the unknown phase. This has two important consequences: First it is required that there is only one unknown phase so that the relative ratios of all other phases may be estimated. Second, this calculation is independent of the temperature at which the experiment is performed, and whether or not thermodynamic equilibrium was reached. Therefore, as long as each experimental sample contains the single unknown phase being targeted, the information from multiple samples resulting from different reaction conditions may be combined by taking the intersection of the conical probability distributions. Accurate predictions of the composition of the unknown phase can then be made despite errors in the experimental quantification of phase ratios and changes to the reaction conditions. The resultant probability distribution may be used to inform the decision of further compositions to sample. Further discussion on handling situations where multiple unknown phases are present is available at the end of the algorithm overview.

“Probabilistic Isolation of Crystalline Inorganic Phases” (PICIP) is a collaborative tool for an experimental chemist, iteratively suggesting new compositions to sample and refining its predictions from the experimental results in a feedback loop. These steps are repeated until the unknown phase is synthesized at a suitably high purity. By ensuring the width of the cones correctly correspond to the experimental uncertainty, this process is optimized to reduce the number of compositions that must be prepared and analyzed, making PICIP an accessible method to those without high throughput phase quantification methods.

PICIP uses an underlying representation of compositional space that treats chemical constraints, such as charge neutrality, as linear equalities that restrict compositions to a lower-dimensional slice of the space. This allows for an affine transformation to be used which consists of a rotation such that the slice is horizontal, and a translation such that the vertical coordinate is zero everywhere, and so can be dropped. This reduces the dimensionality of the representation and ensures it satisfies the chemical constraints (Figure 1). Because the transformation is a rotation followed by a translation, distances and angles are preserved. This allows for a probabilistic model based on the lever rule to be employed. In two or three dimensions, the lower-dimensional representation may be used for isometric visualizations of the phase field. Informative graphic visualizations of the predicted probability density can then be produced, which give a clear understanding of PICIP’s decision making process (Figure 4). However, the explicit need for graphical representations and human interpretation is removed, allowing for phase fields of higher dimension to be explored.

**Figure 1:**
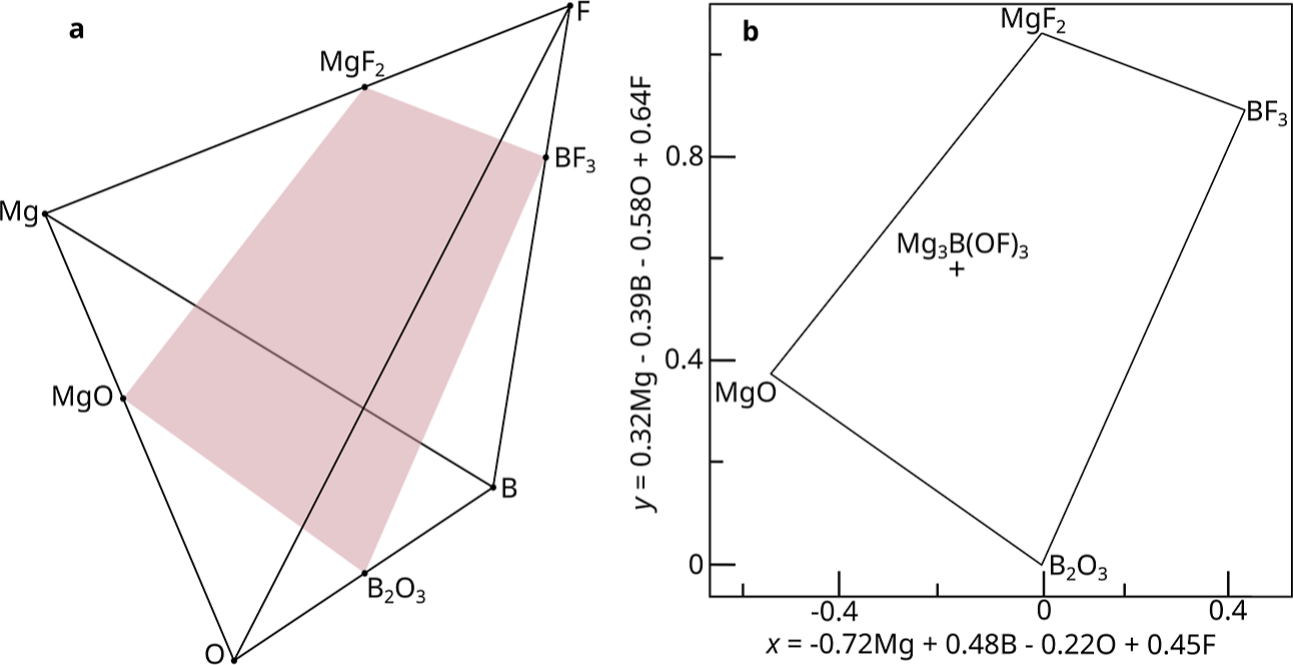
(a) Chemical constraints can be exploited to find lower-dimensional representations of phase fields. A standard tetrahedral barycentric visualization for the Mg—B—O—F phase field is shown, where the corners are the pure elemental compositions. When the formal charges are restricted, the resultant Mg^2+^—B^3+^—O^2–^—F^1–^ phase field is a 2D subspace, shown by the red slice bounded by all four possible charge neutral binary compositions. (b) The Mg^2+^—B^3+^—O^2–^—F^1–^ phase field. Because this charge constrained phase field is 2D, vectors $\vec{\hat{x}}$ and $\vec{\hat{y}}$ may be found which span the phase field and are perpendicular to each other. These are then used as a basis and all valid compositions in the phase field can be uniquely expressed in terms of them. For example the Mg_3_B­(OF)_3_ phase is shown here, which can now be represented with the 2D coordinate vector −0.16 $\vec{\hat{x}},0.58\vec{\hat{y}}$ . This lower dimensional representation preserves the distances and angles between phases.

PICIP has already been used to experimentally isolate new phases. In a recent study^13^ PICIP assisted in the isolation of a new phase, overcoming difficulties imposed by a competing glass field which meant other methods were inappropriate. To estimate PICIP’s performance, and to enable comparisons with alternative autonomous sampling strategies, we also introduce a computational model that simulates the process of isolating an unknown crystalline phase.

## Probabilistic Isolation of Crystalline Inorganic Phases

### Representation

To formulate PICIP it is necessary to fix a mathematical representation for the elemental composition. Given a set of *n* elements, a common method for describing the composition of crystalline phases consisting of those elements is to use an *n*-dimensional vector, where each coordinate of the vector gives the relative amount of the respective element, referred to here as the “standard representation.” These coordinates represent relative amounts of elements, and thus they are all greater than zero, and their sum is one. Geometrically, these conditions equate to constraining these composition vectors in the standard representation to a (*n* – 1) simplex, e.g. a triangle for three elements, a tetrahedron for four elements, etc. This allows for visual representations of phase diagrams which are one dimension fewer than the number of elements, commonly seen in the barycentric representations for phase diagrams (Figure 1a).

Ionic crystalline phases satisfy an additional constraint: charge neutrality. In situations where all elements in the system have a single formal charge, this constraint introduces a second linear equality. Geometrically, this further constrains composition vectors in the standard representation to a (*n* – 2)-dimensional slice of the (*n* – 1) simplex. For four elements, such as the Mg^2+^—B^3+^—O^2–^—F^1–^ phase field, this is a slice of the tetrahedron (Figure 1a). This phase field will be used as the example throughout the explanation of PICIP.

Because the *n*-dimensional composition vectors in the standard representation are constrained to a (*n* – 2)-dimensional space, it is possible to use an alternate representation for compositions consisting of (*n* – 2)-dimensional vectors. This is referred to here as the “constrained representation”. An affine transformation, consisting of a rotation of the phase space followed by a translation, is used for this purpose. Figure 1b shows the example Mg^2+^—B^3+^—O^2–^—F^1–^ phase field when plotted using coordinates obtained from such an affine transformation. The affine transformation can be visualized as rotating the tetrahedron so that the 2D red slice is parallel to the *xy* plane, and then lowering until the red slice lies on the *xy* plane. The “*z*” coordinate is then guaranteed to be zero everywhere and so can be dropped, leaving a 2D representation; the *xy* coordinates. Because the 2D representation remains valid under any in-plane rotation, there is some flexibility in defining the $\vec{\hat{x}}$ and $\vec{\hat{y}}$ directions. This freedom reflects that any orientation of the phase diagram is equivalent. In this example, we select $\vec{\hat{y}}$ to point from B_2_O_3_ toward MgF_2_, and $\vec{\hat{x}}$ such that MgO is on the left.

$$\begin{array}{ll} \vec{\hat{x}} & =\left[-0.72,0.48,-0.22,0.45\right]\\ \vec{\hat{y}} & =\left[0.32,-0.39,-0.58,0.64\right] \end{array}$$

where coordinates have been rounded to two decimal places. Denoting **p** as the composition of interest, **B** as the 2 × 4 truncated rotation matrix, and **c** as some chosen fixed composition, the *xy* coordinates of **p** can be calculated as

1 $$\left[\begin{array}{l} p_{x}\\ p_{y} \end{array}\right]=\mathrm{B⃗}\times \left[\begin{array}{l} p_{Mg}-c_{Mg}\\ p_{B}-c_{B}\\ p_{O}-c_{O}\\ p_{F}-c_{F} \end{array}\right]$$

where the elemental subscript refers to the relative amount of that element in the respective composition. For this specific phase field the matrix **B** has two rows and four columns because it maps the four-dimensional vectors in the standard representation onto the two-dimensional constrained representation. Each row of **B** is a unit vector in the constrained representation, and together they define the 2D coordinate system. The subtraction by **c** recenters the composition space around the chosen reference point before projection. The result is a consistent 2D representation of any composition satisfying the constraints, suitable for visualization and analysis. In general, for a charge constrained phase field consisting of *n* elements, the matrix **B** will have *n* columns and *n* – 2 rows. Further details of how **B** is constructed to incorporate both the charge neutrality and normalization constraints are given in Supporting Information Section S4.

We now focus on the constrained representation for the Mg^2+^–B^3+^–O^2–^–F^1–^ phase field. The fixed translation vector **c** is first chosen as B_2_O_3_ or [0,0.4,0.6,0] in the standard representation. Note that this means the *xy* coordinates of B_2_O_3_ will always be (0,0) in the constrained representation. Now consider a known quaternary in this phase field: Mg_3_B­(OF)_3_ or [0.3,0.1,0.3,0.3] in the standard representation. Using eq 1 we would find the *xy* coordinates to be approximately [-0.16,0.58] (Figure 1b). Similarly, every unique charge neutral composition in the Mg^2+^—B^3+^—O^2–^—F^1–^ phase field will have a unique 2D vector in the constrained representation, and this representation preserves the angles and distances between compositions in their standard representation. These coordinates may then be used both for visualization and computation.

This technique reduces the dimension of the chemical space that is to be explored, while implicitly ensuring compositions which are either not charge neutral or are scaled duplicates are never suggested as new sample points. For situations where elements do not take a single formal charge, such as intermetallic phase fields, it is simple to relax the charge constraint and transform to a representation one dimension fewer than the number of elements. This transformation results in an identical geometry to regular ternary or quaternary representations using equilateral triangles or tetrahedrons, although an orthonormal basis is used instead of the more common barycentric representations.

In principle, additional constraints can be added that further reduce the dimensionality of the representation. For example, one might constrain the total number of anions to be twice the number of cations within a composition. For the present example, the Mg^2+^—B^3+^—O^2–^—F^1–^ phase field would reduce to a 1D line between MgF_2_ and the composition BOF. For the case of mixed valence elements then the charge neutrality constraint instead becomes a pair of linear inequalities. Consider the Mn—O phase field where it is assumed the oxidation state of Mn is between +2 and +4. The maximum positive charge of Mn is +4 and the negative charge of O is −2 so we know 4*a* – 2*b* ≥ 0 where *a* and *b* are the molar amounts of Mn and O respectively. Correspondingly the minimum charges of Mn is +2 and so we know 2*a* – 2*b* ≤ 0. Conceptually each of these linear inequalities define a slice through the phase field, with one side of the slice remaining chemically feasible. The result will be a representation of one dimension fewer than the number of elements, but with a smaller volume than if charge neutrality is completely ignored. In this example, only the section of the Mn—O line between MnO and MnO_2_ satisfies the two inequalities.

### Inputs

PICIP proceeds by considering compositions in the constrained representation described above. It predicts the composition of a single unknown phase in a mixed phase sample based on the initial composition of the sample, *s* and the weighted average composition of the known phases in that sample, *k*. This requires accurate phase identification of all of the known crystalline phases in that sample, along with quantitative phase analyses. Powder X-ray diffraction (PXRD) is a technique that readily provides such bulk information within a single data set. PXRD can be used to identify multiple phases within a solid sample.^14,15^ Once a PXRD pattern has been obtained, Rietveld refinement can be used to estimate the relative mass fractions of the crystalline phases present. These can then be converted to the required molar fractions. We assume that it is not possible to calculate the relative mass fractions of any unknown crystalline phases.

The relative mass fractions returned from Rietveld analysis will include an experimental error. The extent of this error will depend on multiple factors and is likely to be larger in instances where the Rietveld refinement method itself may be limited, for example, when significant peak overlap exists between the patterns of different phases or where phases are present in small amounts leading to relatively weak diffraction peaks. In both cases, the input into PICIP is reliant upon both quality of data and quality of the analysis performed. PICIP accounts for this by assuming that the estimated relative mass fractions are sampled from a normal distribution centered on the measured value and with standard deviation equal to a user provided hyper-parameter, referred to as the PICIP error (σ_P_). The estimated standard deviation on the refined values computed by the refinement software is a good starting point for the choice of the PICIP error. The PICIP error can then be adjusted to account for different levels of experimental error on the relative mass fractions. This then directly controls the spread of the inferred probability distribution for the composition of the unknown phase. As a result, the PICIP error can also account for other sources of experimental error, such as precursor loss or trace amounts of other unidentified phases. In addition, mathematical assumptions made for the inference process itself mean that factors relating to the geometry of the phase diagram being explored affect the optimal choice of PICIP error. The relationship between the experimental error and the optimal PICIP error is explored in the results section.

PICIP is demonstrated on the Mg^2+^–B^3+^–O^2–^–F^1–^ phase field by treating Mg_3_B­(OF)_3_ as an unknown crystalline phase to be isolated (Figure 2a). First we choose *s* with composition Mg_0.41_B_0.02_O_0.31_F_0.26_ (Figure 2b). Given the phase diagram in Figure 2, accurate Rietveld analysis would return the relative molar fractions of the crystalline phases as 0.2Mg_3_B­(OF)_3_ + 0.3MgF_2_+0.5MgO. However, as Mg_3_B­(OF)_3_ is “unknown”, then the relative molar fractions PICIP would receive from accurate phase quantification would be 0.38MgF_2_ + 0.63MgO.

**Figure 2:**
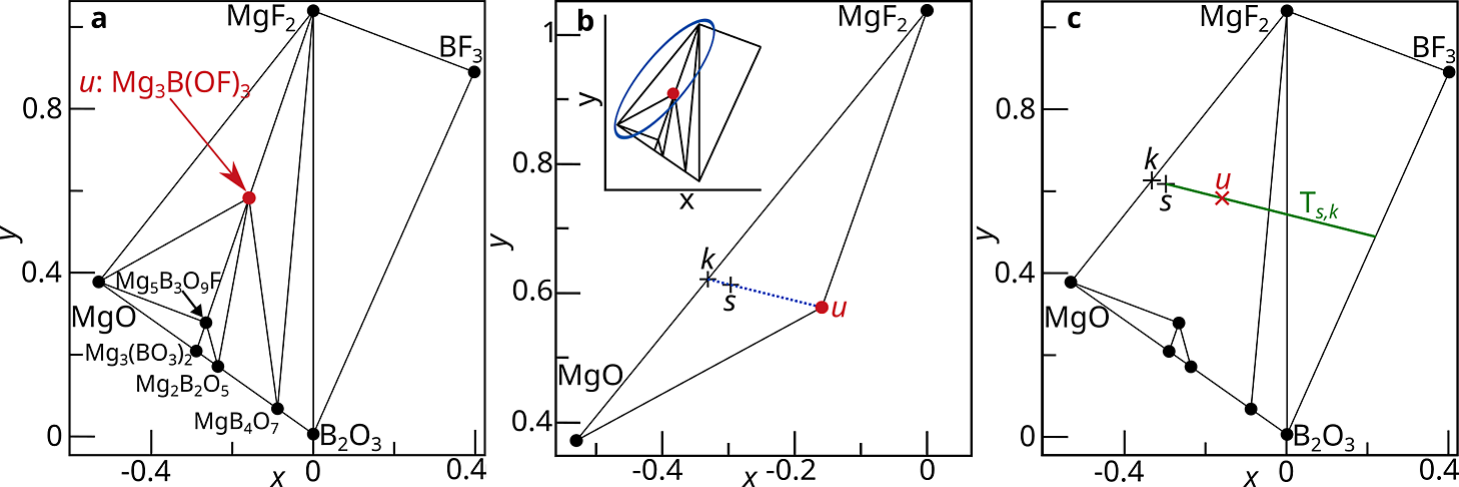
(a) The Mg^2+^—B^3+^—O^2–^—F^1–^ phase field. The known phase highlighted in red, Mg_3_B­(OF)_3_, is assumed to be unknown and labeled *u*. (b) Consider this small section of the phase diagram containing the unknown phase and two known phases. At thermodynamic equilibrium, the sampled composition, labeled *s*, will be a mixture of each of these phases. From the relative molar fractions of the known crystalline phases, PICIP can calculate the average composition of the known crystalline phases, labeled *k*. Importantly *k*, *s* and *u* will all be colinear. (c) If *k* and *s* are known exactly, then the line segment extending from *s* away from *k* will contain *u*. This line segment is referred to as the estimated direction, and is denoted *T*_*s*,*k*_.

### Algorithm Overview

Accurate relative molar fractions of the known phases can be used to calculate a line segment, referred to here as the “estimated direction”, which will contain the unknown phase. This construction relies on the fact that the sampled composition, *s*, will be a weighted average of the resultant crystalline phases’ compositions, with the weighting given by their respective molar fractions. Importantly, this weighted averaging can be considered as a nested process. First, the relative molar fractions of the known crystalline phases can be used to calculate the average composition of the known crystalline phases, denoted *k*. Second, *s* can be expressed as a weighted average of *k* and the unknown crystalline phase’s composition, denoted *u*. The weighting for the second average is unknown under the aforementioned assumption that it is not possible to calculate the relative mass fractions of any unknown crystalline phases. Despite this, because *s* is an average of *u* and *k*, all three points will be colinear. Consequently, *u* must lie on the line segment starting at *s* and extending away from *k*. This line segment is the estimated direction, and is denoted *T*_*s*,*k*_.

Applying this logic to the sampled composition in the Mg^2+^–B^3+^–O^2–^–F^1–^ phase field, the relative molar fractions of known phases are 0.38MgF_2_ + 0.63MgO. This results in *k* being the composition Mg_0.44_B_0_O_0.31_F_0.25_. Accordingly, *T*_*s*,*k*_ is a line segment starting at *s* and continuing in the direction −0.77Mg + 0.56B – 0.07O + 0.28F (Figure 2c).

The relative molar fractions obtained from Rietveld analysis will be estimates, and so the calculated value of *k* will be incorrect. To account for this, rather than representing *k* as a singular point, it is represented with a multivariate normal distribution, K, centered on the measured value of *k* and with a covariance matrix calculated from the PICIP error. This distribution is then discretized onto a finite set of points, each with an associated probability of being *k*. A set of estimated directions is then generated, one for each point, where the probability of each estimated direction containing the unknown crystalline phase is equal to the probability of its generating point being *k*. This set of lines is then interpolated onto a discretized grid, resulting in a probability density giving the probability of each point in the phase diagram being the composition of the unknown phase. Information from multiple sampled compositions can be combined by multiplying normalized probability densities together. Detailed calculations of K from the PICIP error (Section S7) and the construction of the probability density (Section S6) are provided in the Supporting Information.

Applying the procedure outlined above to the sampled composition in the Mg^2+^–B^3+^–O^2–^–F^1–^ phase field, we now assume that the relative mass fractions deviate from their true values by 2 wt %. Consequently, the relative molar fractions passed to PICIP are 0.394MgF_2_ + 0.606MgO, which yields *k* = Mg_0.43_B_0_O_0.30_F_0.26_ (Figure 3a). The PICIP error can then be chosen to control the size of K, which in turn affects the induced probability density for the unknown crystalline phase (Figure 3b,c). Note that in this example, for the purpose of explanation, we assume thermodynamic equilibrium is reached in order to calculate *k* from *s* and *u*. In general this assumption is not required for PICIP to be used, as *k* is estimated experimentally from the estimated relative mass fractions.

**Figure 3:**
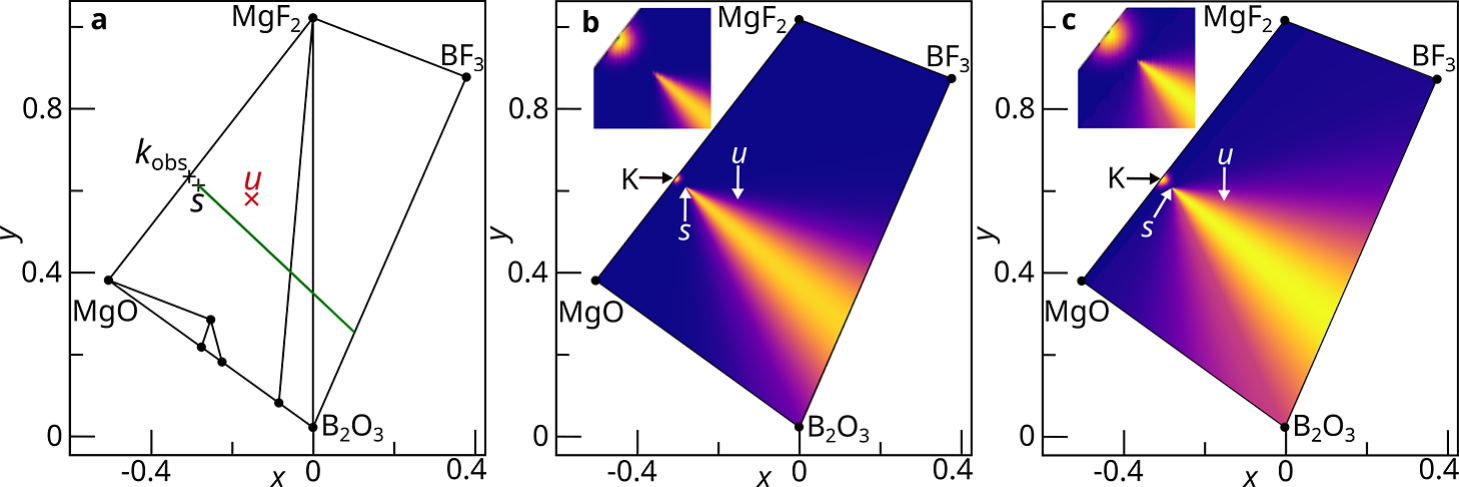
(a) *k*_obs_ is the measured average composition of known phases, where the measured relative mass fractions estimated by Rietveld analysis deviate from their true values by 2 wt %. *s* and *u* are the sample composition and unknown crystalline phase, respectively. (b) Heatmap showing the probability density for the unknown crystalline phase. K is the normal distribution PICIP uses to account for the expected error on *k*_obs_. In this case the PICIP error is chosen to be 1 wt % (σ_P_ = 0.01). By projecting K through the sampled composition *s*, a cone shaped probability density can be computed, giving the probability of each composition in the phase field being the composition of the unknown crystalline phase. (c) In this case the PICIP error is chosen to be 2 wt % (σ_P_ = 0.02), resulting in a broader normal distribution for K and a correspondingly wider cone.

The mean of this probability density is then suggested as the next composition to sample. A batch of samples can also be suggested rather than a single point, this will consist of the mean and further points randomly sampled from the probability density. After the composition(s) have been sampled the probability density can be updated allowing for an automated iterative sampling strategy to isolate the unknown crystalline phase.

This outline of PICIP’s decision-making process is most easily understood from the perspective of an isothermal exploration of a phase field but is not limited to it. The probability density associated with each sample is defined solely in compositional space: it estimates the composition of the unknown phase without information on the reaction conditions used. Because these compositional probability densities can be constructed and combined independently of reaction conditions, temperature-dependent phase diagrams may be explored. The limitation is that achieving a high-purity sample of the unknown phase may require specific reaction conditions which PICIP does not provide any information on.

This procedure is based on the assumption that there was a single unknown phase in the mixed phase sample. For situations where multiple unknown phases are present, then the estimated direction will no longer point toward the unknown phase and instead point toward the average composition of the unknown phases. This is problematic as the relative ratio between the unknown phases will change from sample to sample, and so their average composition is not constant. In the presence of multiple unknown phases, PICIP will still suggest compositions that reduce the amount of the known phases detected. This should result in diffraction peaks from the multiple unknown phases becoming more easily identifiable, as their relative intensity compared to the known phases will increase. Thus, sufficient experimental sampling of the region of chemical space defined by the probability cone is important. Where possible, changes in the relative intensities of the peaks arising from unknown phases may then be used to indicate the direction in which individual phases lie. Incorporation of information present within the intensities and positions of peaks assigned to unknown phases into a fully automated method is a potential area for future development of PICIP. In addition, once PICIP has directed compositions toward those in which the unknown phases are present in sufficiently high quantities, it is possible to utilize complementary discovery techniques such as EDX, electron diffraction, or single crystal X-ray diffraction to gain more information.

## Simulating Phase Isolation

In order to evaluate the performance of autonomous phase isolation algorithms such as PICIP, we have developed a computational model which can simulate the process of phase isolation. Given a suggested sample composition, this model assumes that thermodynamic equilibrium is reached, and then calculates which stable phases would form and their expected relative molar fractions. These fractions are then perturbed to simulate experimental error.

For computational phase diagrams, a convex hull is constructed from computed formation energies of all candidate phases in the system. By identifying the facet of the hull that contains the sampled composition, the resultant stable phases are found as the vertices of this facet. The relative molar fractions are then found with the lever rule: determining the unique combination of the stable phases whose weighted average matches the starting composition, with the weights being the molar fractions.

For experimental phase diagrams, the resultant stable phases are identified as the vertices of the region which contains the sampled composition. Their relative molar fractions are determined in the same way as for the computational phase diagrams.

Thermodynamic equilibrium must be assumed in order to calculate these values, and consequently provide a computational environment capable of assessing PICIP’s ability to predict the composition of an unknown phase. This does not limit PICIP to situations where thermodynamic equilibrium is reached, or even specific isotherms. The only requirement is the identity and estimated relative molar fractions of all but one of the resultant phases.

The Materials Platform for Data Science (MPDS) is used as a source for experimentally determined phase diagrams.^16^ Materials Project provides phase diagrams containing experimentally reported phases alongside the computed energies and tools used to identify the resultant stable phases and their relative molar fractions.^17^

To simulate the relative mass fractions of the known crystalline phases calculated from Rietveld analysis, the true mass fractions need to be perturbed by some amount, reflecting the experimental error. Mass fractions are constrained to the unit simplex, which is to say they are non-negative, lie between zero and one, and the total sum of all mass fractions is unity. The standard normal distribution is therefore not suitable due to these bounds. Compositional data analysis is the study of statistical distributions with such constraints. The two main distributions arising from this field are the Dirichlet distribution^18^ and the logistic normal distribution.^19^ However, in this work only phase fields where at most three different crystalline phases can be present at thermodynamic equilibrium are simulated and so the number of known crystalline phases will be at most two. This allows for a simpler solution which is more easily relatable to the errors in Rietveld analysis reported from round robins such as in.^20^ Given the true mass fractions [*k*_1_, *k*_2_], the simulated mass fractions are [*k*_1_^*^,*k*_2_^*^] where *k*_1_^*^ is sampled from a normal distribution with mean *k*_1_ and standard deviation σ truncated to the segment [0,1] and *k*_2_^*^ = 1 – *k*_1_^*^. We assume that samples that have a higher proportion of the unknown phase will have a less accurate relative mass fractions, as the relative amount of the phases being quantified is lower. For this reason we define a new parameter, the experimental error (σ_E_). The standard deviation σ of the normal distribution that *k*_1_^*^ is sampled from is then calculated as

2 $$\sigma =\sigma_{E}\left(1+w_{u}\right)$$

where *w*_u_ is the relative mass ratio of the unknown phase. To test the applicability of this algorithm to different experimental situations, a range of experimental errors will be used.

This simulation environment can be used to evaluate the performance of PICIP for different phase fields and degrees of experimental error. For the Mg^2+^—B^3+^—O^2–^—F^1–^ phase field with σ_E_ = 0.02 an illustration of PICIP’s iterative isolation process is provided (Figure 4a–c). In this example σ_P_ = 0.02 and the batch size is three.

**Figure 4:**
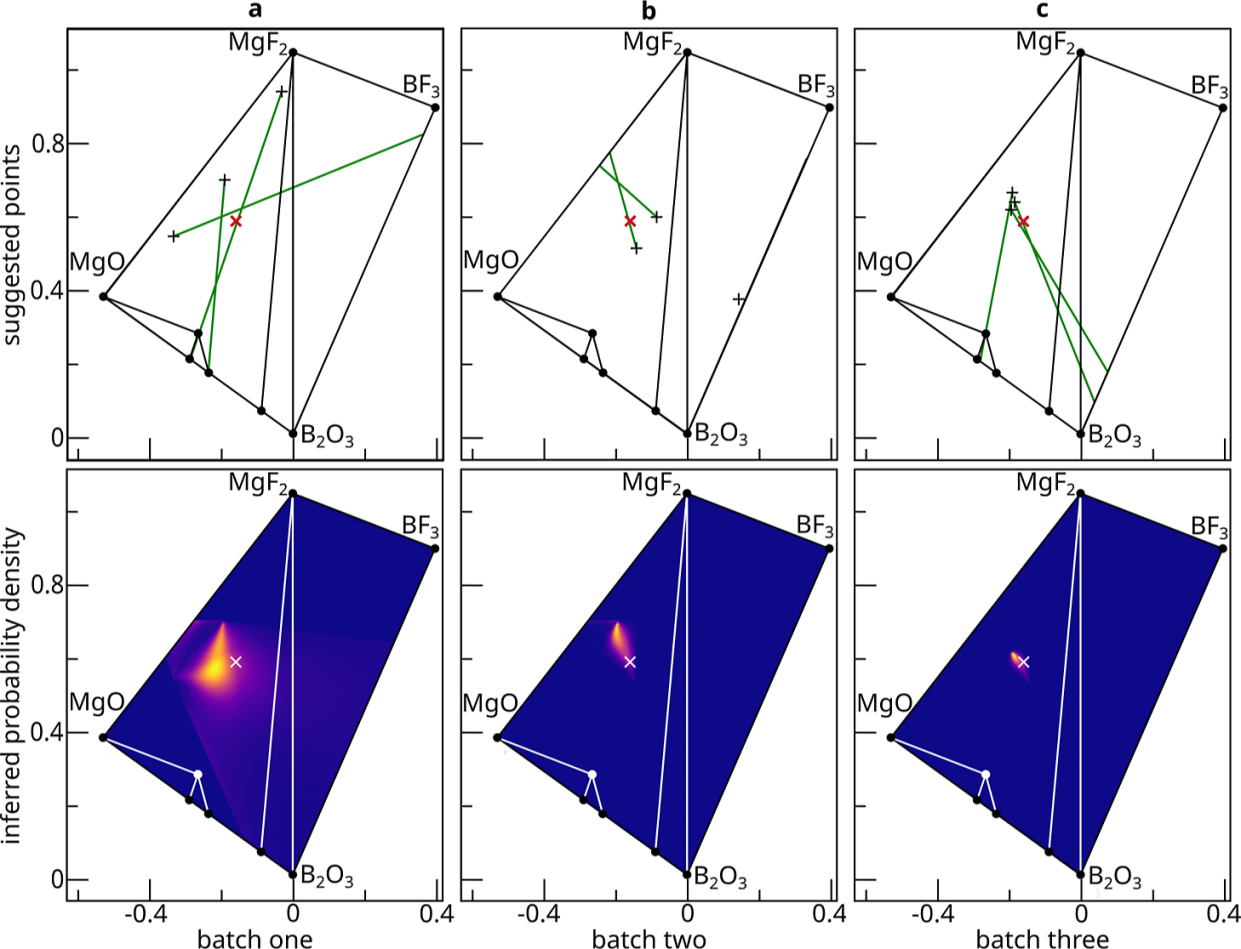
Illustration of how PICIP chooses future compositions to sample. This simulation was an exploration of the Mg^2+^—B^3+^—O^2–^—F^1–^ phase field, where the experimental error is 2 wt %, the PICIP error is 2 wt %, and the batch size is three. (a) In the first batch three compositions are randomly selected, shown as black crosses in the top panel. Each contain some of the unknown crystalline phase, *u* in red (top) or white (bottom). For each sampled composition, the simulating environment computes the relative mass fractions of the known crystalline phases, perturbs them, and then passes them to PICIP. The green lines for each sampled composition are the estimated direction, *T*_*s*,*k*_, and indicate where the unknown crystalline phase would be if the received ratios were accurate. The combined probability density resulting from the first batch is shown in the bottom panel. (b) A second batch of three samples is then sampled from this probability density, and the probability density is updated. Note that in this batch one sample would not have contained any of the unknown phase, and so it is disregarded. (c) A final third batch of three samples is sampled from the updated probability density, with the combined probability density from all nine samples shown.

## Methods

To estimate how successful PICIP is at isolating unknown crystalline phases, the previously described simulation environment is used. Each simulation begins with a random composition that contains some of the unknown phase, PICIP then iteratively suggests up to 15 further compositions to sample. For each simulation the percentage purity of the unknown crystalline phase is calculated for each suggested sample in that simulation. The maximum percentage purity of the unknown crystalline phase, denoted “Purity Score”, is then recorded as a function of the number of samples, which is used as the evaluation metric.

To examine PICIP’s performance over a range of experimental conditions, we simulate different phase fields, degrees of experimental error, and batch size.

For each phase field, all crystalline phases that are not on the border of the phase field (i.e., any phase that contains all of the elements) are considered to be possible candidate unknown phases, and the algorithm is tasked with isolating them. These candidate unknown phases are shown in Table 1. All unknown phases have been experimentally reported. The first phase field explored is the Li^1+^—Al^3+^—B^3+^—O^2–^ phase field, using an experimental phase diagram obtained from the MPDS.^16^ In this phase field there are 6 candidate unknowns. The second phase field explored is the Mg—Al—Cu phase field, using a computed phase diagram obtained from Materials Project.^17^ As this is an intermetallic phase field, no assumptions about charge states are made. Owing to the lack of a charge constraint, the representation for compositions in this phase field is only one dimension lower than the number of elements. In this phase field there are 3 candidate unknowns. The third phase field explored is the Mg^2+^—B^3+^—O^2–^—F^1–^ phase field, using a computed phase diagram obtained from the Materials Project. In this phase field there are 2 candidate unknowns.

**Table 1:** 11 Crystalline Phases Treated as Unknown for the Purpose of the Simulation^a^

unknown	phase field	source
Mg_3_B(OF)_3_	Mg^2+^—B^3+^—O^2–^—F^1–^	a
Mg_5_B_3_O_9_F	Mg^2+^—B^3+^—O^2–^—F^1–^	a
MgAl_2_Cu	Mg—Al—Cu	a
Mg_2_Al_5_Cu_6_	Mg—Al—Cu	a
Mg_3_(AlCu_2_)_2_	Mg—Al—Cu	a
Li_2·46_Al_0·18_BO_3_	Li^1+^—Al^3+^—B^3+^—O^2–^	b
LiAl_7_B_4_O_17_	Li^1+^—Al^3+^—B^3+^—O^2–^	b
Li_2_AlB_5_O_10_	Li^1+^—Al^3+^—B^3+^—O^2–^	b
Li_2_AlBO_4_	Li^1+^—Al^3+^—B^3+^—O^2–^	b
Li_3_AlB_2_O_6_	Li^1+^—Al^3+^—B^3+^—O^2–^	b
LiAlB_2_O_5_	Li^1+^—Al^3+^—B^3+^—O^2–^	b

a Each candidate phase contains every element in its respective phase field, and so does not lie on an edge. All phases, including those used in computational phase diagrams, have been experimentally reported. Source key: (a) Materials Project,^17^ (b) Materials Platform for Data Science (MPDS).^16^ The data from MPDS was originally published in ref 21.

The amount of experimental error was chosen with the guidance of experimental colleagues to represent small, medium, and large errors in the estimation of the relative mass ratios in the presence of an unknown phase. Here, we use 2, 5, and 10 wt % (σ_E_ ∈ [0.02, 0.05, 0.1]). For the PICIP error, the values used are 1, 2, and 4 wt % (σ_P_ ∈ [0.01, 0.02, 0.04]). For the batch size, either one, three or five samples are suggested at each stage. For each phase field the experimental error, PICIP error and batch size are varied independently of each other. For each choice of unknown phase, experimental error, PICIP error and batch size, the simulation is repeated 3000 times, and the distribution of the Purity Score across these repeats is considered.

The standard deviation on the simulated relative mass fractions is assumed to depend on the amount of unknown phase present (eq 2). Therefore, the chosen values of experimental error (σ_E_ ∈ [0.02, 0.05, 0.1]) correspond to a Root Mean Squared Error (RMSE) on the simulated relative mass fractions of 3, 7, and 13 wt % respectively when a third of the sample consisted of the unknown phase. Additionally, because a truncated normal distribution is used to ensure that simulated relative mass fractions are between zero and one, this reduces the standard deviation when the true relative mass fractions are close to zero or one. This is rarely a significant effect and so is ignored in the calculation of the RMSE.

A consequence of the assumptions made in the formulation of the probability density is that it is possible for the probability density resulting from multiple sampled compositions to be zero everywhere, i.e. the “cones” do not intersect. From this position it is impossible to suggest subsequent sampled compositions, and so the simulation terminates. While this phenomenon would be useful experimentally, as it highlights that either one or more assumptions is invalid in the specific case, or that there is an unusually high amount of measurement error, for modeling purposes it is treated as a failure. Reported results on the distribution of the Purity Score ignore these failures, and the failure rate is reported separately.

The application of PICIP in this simulation environment is implemented in Python. All code and the required libraries are available at https://github.com/lrcfmd/PICIP. All simulation runs were performed on Intel CPUs with at least 16Gb of RAM, running on Ubuntu 22.04 with Python 3.10.

## Results and Discussion

For each of the 11 candidate unknown phases the PICIP error, experimental error and batch size are varied and the Purity Score, as defined in the Methods section, is used as the main indicator of success. Individual plots of the phase diagram, Purity Score vs number of samples and failure rate are shown for every candidate unknown phase in Figures S1–S55. Here we present plots averaged over different unknowns to extract general trends in the performance of PICIP under different conditions, as well as specific plots to explore the effect of the PICIP error.

We begin by considering serial sampling of the phase field (a batch size of one) and investigating the performance of PICIP under different levels of experimental error. Consider the median Purity Score vs number of samples averaged across all unknowns, for PICIP error values of 0.01, 0.02 and 0.04, and experimental error values of 0.02 (Figure 5a) and 0.1 (Figure 5b). PICIP performs best when the experimental error is lowest (σ_E_ = 0.02), with a median Purity Score greater than 90% after 4 samples. Increasing the experimental error decreases the convergence rate, with 6 samples required to exceed a median Purity Score of 90% when σ_E_ = 0.1. This suggests PICIP can successfully isolate an unknown phase across a range of different experimental errors, but performs better when the experimental error is lower. For a fixed experimental error, tuning the PICIP error has little effect on the average performance, with the exception of a slightly slower convergence for a large PICIP error when the experimental error is small.

**Figure 5:**
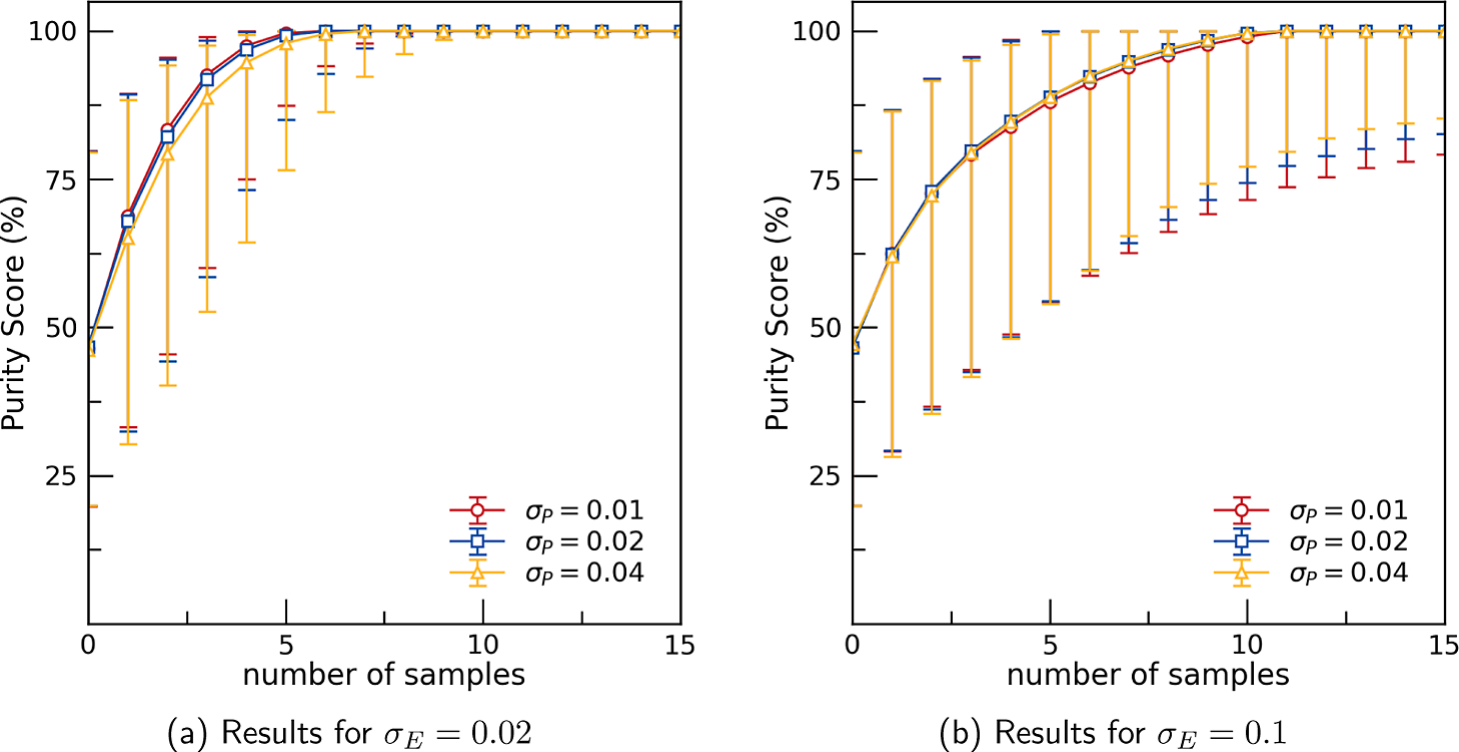
Evolution of the Purity Score, defined as the highest obtained purity of the unknown phase up to a given point in the experiment, plotted against the number of sampled compositions. Each experiment starts with a randomly chosen composition which contains the unknown phase, and at each sampling step, PICIP proposes a new composition expected to increase the unknown phase’s purity. The curves represent the median Purity Score across 3000 simulation repeats, and the error bars indicate the 16th and 84th percentiles, capturing the central 68% of the distribution. Results are shown for different values of experimental error (σ_E_) and PICIP error (σ_P_), averaged over all candidate unknown phases.

To find cases where tuning the PICIP error can lead to significant benefits, we examine below two phase diagrams with extreme differences in phase diagram geometry, where the isolation of the unknown phases MgAl_2_Cu (Figure 6a) and Li_2_AlB_5_O_10_ (Figure 6b) is compared for σ_E_ = 0.01. For the MgAl_2_Cu phase a larger PICIP error leads to faster convergence whereas for the Li_2_AlB_5_O_10_ phase the inverse is true. These differences are likely due to a number of factors relating to the geometry of the diagram.

**Figure 6:**
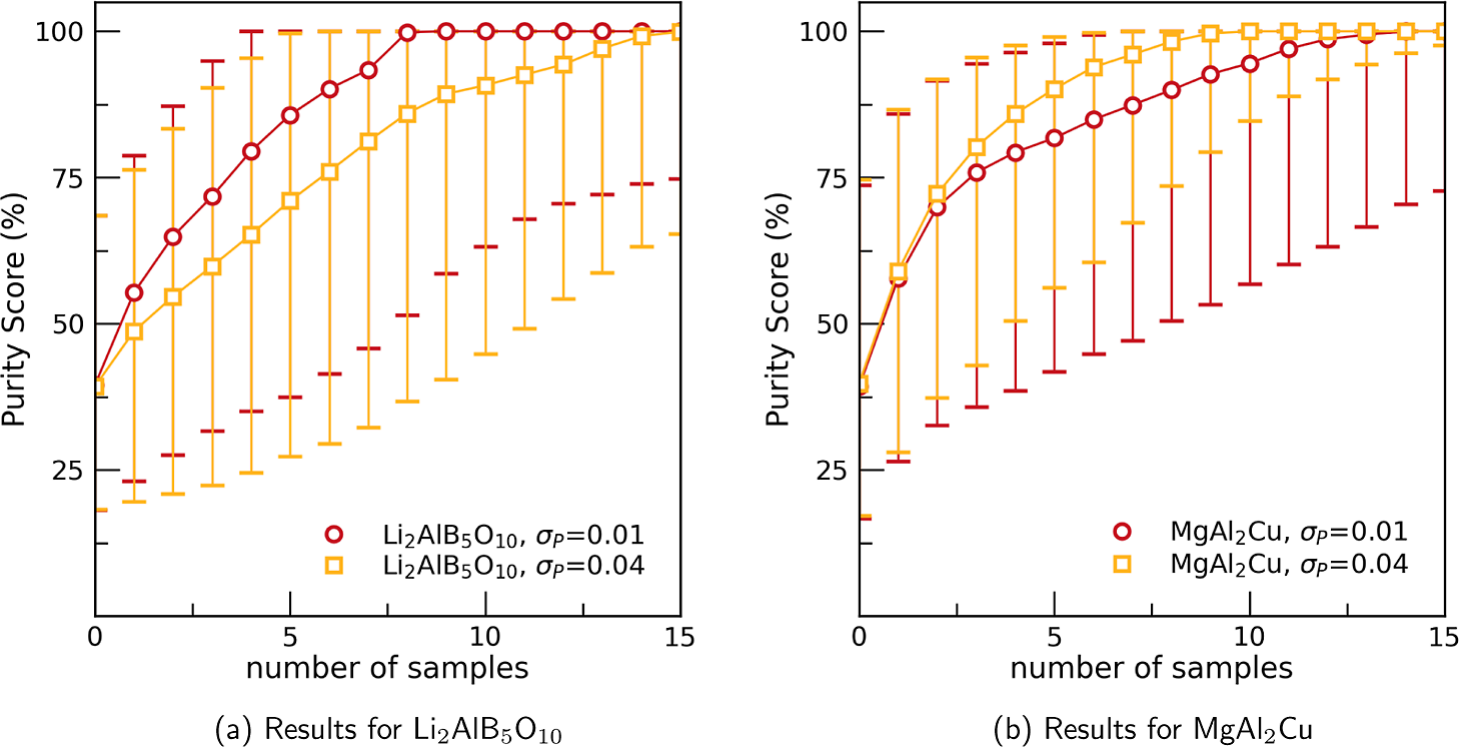
Effect of different choices of the PICIP error (σ_P_) on the median Purity Score for the unknown phases MgAl_2_Cu and Li_2_AlB_5_O_10_. The experimental error is fixed at 10 wt % (σ_E_ = 0.1) and the error bars show the 16th and 84th percentiles, respectively.

One geometric metric to consider is the area of the phase diagram which forms the unknown phase. As an alternative indication of performance, we denote the median Purity Score after 6 samples as Score_6_. The difference in Score_6_ is compared for PICIP error values of 4 and 1 wt % (σ_P_ ∈ [0.04, 0.01]) (Figure 7). For unknowns in regions with areas below 0.1, performance is better with the smaller value of PICIP error, however this behavior reverses as the regions containing unknowns have higher areas. These results suggest it is better to use a larger PICIP error when the area is larger.

**Figure 7:**
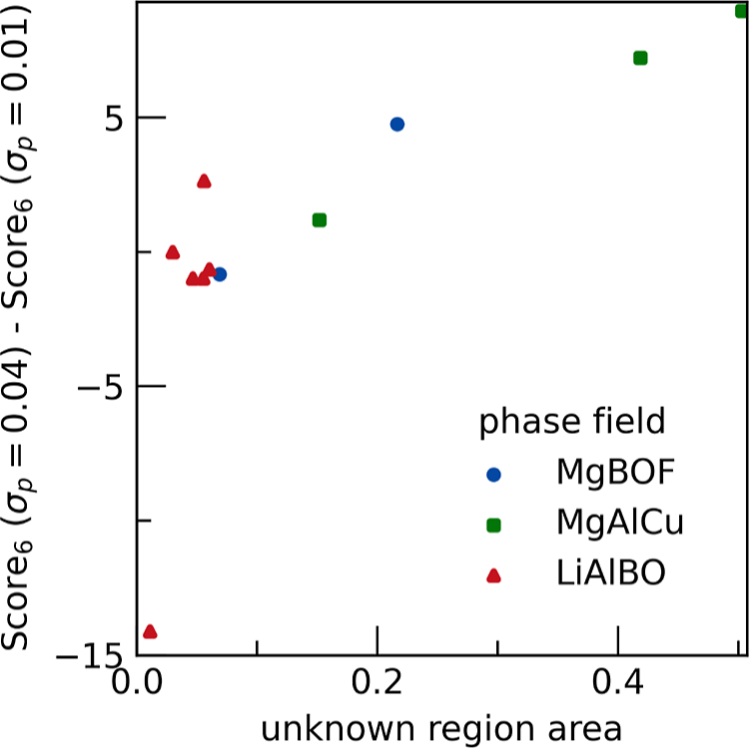
Effect of the area of the region containing the unknown phase on the optimal choice of PICIP error. Score_6_ is the median Purity Score obtained after 6 samples. The plotted difference in Score_6_ compares PICIP errors of 4 and 1 wt % (σ_P_ ∈ [0.04, 0, 01]), where a larger positive value indicates better performance using the 4 wt % error. The horizontal axis shows the area of the region containing the unknown phase; for example, for Mg_3_B­(OF)_3_, this area is the sum of all triangles in (Figure 2a) that have Mg_3_B­(OF)_3_ as a vertex.

Another geometric metric to consider is the compositional distance between known phases present in a sample. This is due to the fact that the experimental error is incorporated as an error on the normalized relative mass fractions. Consequently, when the two known phases are further apart, the relative error translates to a greater distance between the estimated and true average compositions of the known phases (Figure 8a,b). The estimated direction is then less accurate and so the center of the induced probability density will be further from *u*.

**Figure 8:**
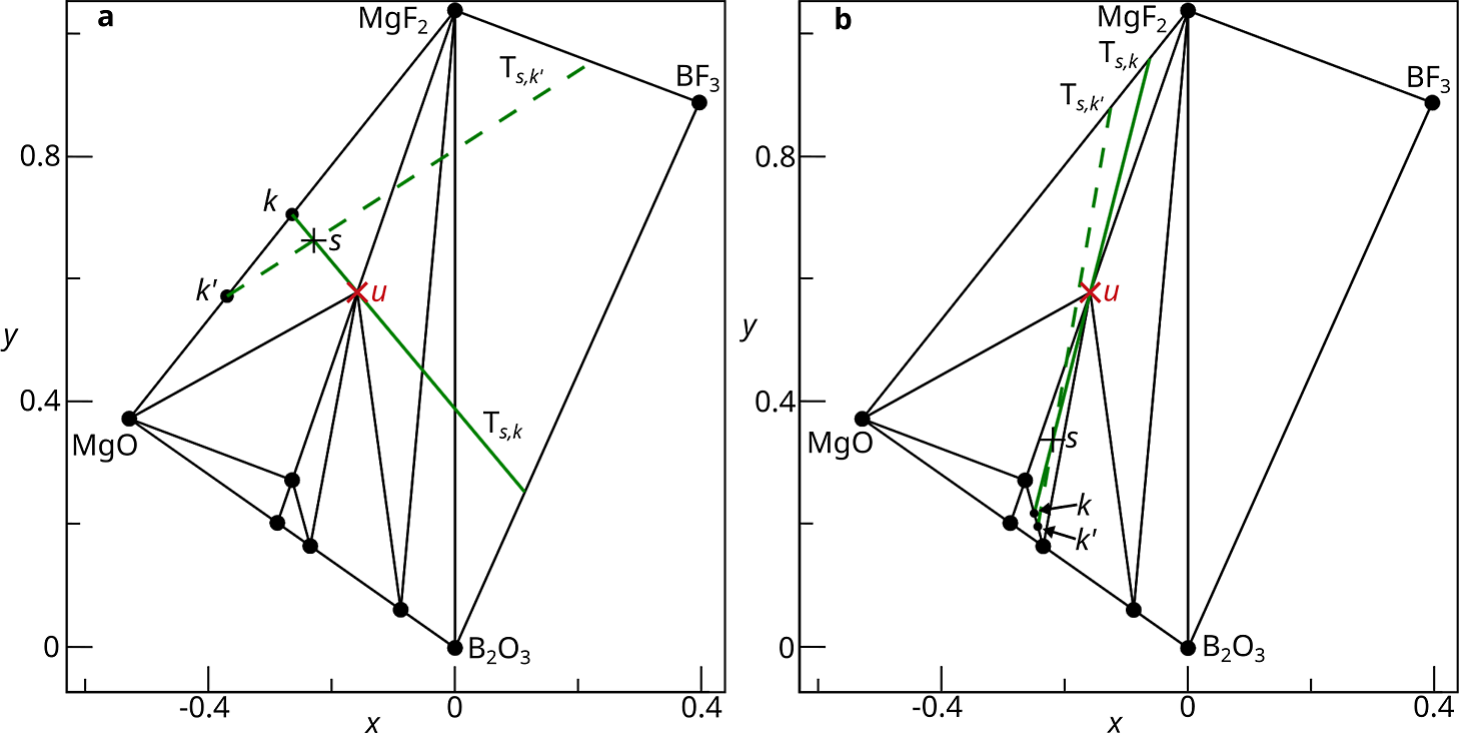
(a) A sample, denoted *s*, has been chosen with composition 1/3 MgF_2_ + 1/3 MgO + 1/3 Mg_3_B­(OF)_3_. *k*, the true average composition of the known phases, is then the midpoint of the two known phases. An experimental error corresponding to a change of 0.2 in the relative molar ratios results in the estimated average composition of the known phases, *k*′, with composition 0.3 MgF_2_ + 0.7 MgO. (b) Moving the sample to the composition 1/3 Mg_5_B_3_O_9_F + 1/3 Mg_2_B_2_O_5_ + 1/3 (Mg_3_B­(OF)_3_), we have *k* as the midpoint of Mg_5_B_3_O_9_F and Mg_2_B_2_O_5_. *k*′ is again calculated as a change of 0.2 on the relative molar fractions, with composition 0.3 Mg_5_B_3_O_9_F + 0.7 Mg_2_B_2_O_5_. In this example, the distance between the points *k* and *k*′ is much less than in (a). This is because the distance between the two known phases is much less.

The failure rate of PICIP is dependent on the PICIP error and the experimental error (Figure 9). The failure rate indicates the proportion of times that PICIP is unable to proceed as the probability density has become zero everywhere. The failure rate increases when the experimental error increases, an important confirmation that a key reason for failure is inaccurate estimations of the relative weight fractions. For the smallest PICIP error (σ_P_ = 0.01) the failure rate is consistently highest. This highlights that underestimating the amount of experimental error will lead to observed measurements contradicting each other. In an experimental application, failure of the PICIP method suggests there is a high amount of experimental error. If the experimental error cannot be reduced, then increasing the PICIP error may allow the experiment to proceed.

**Figure 9:**
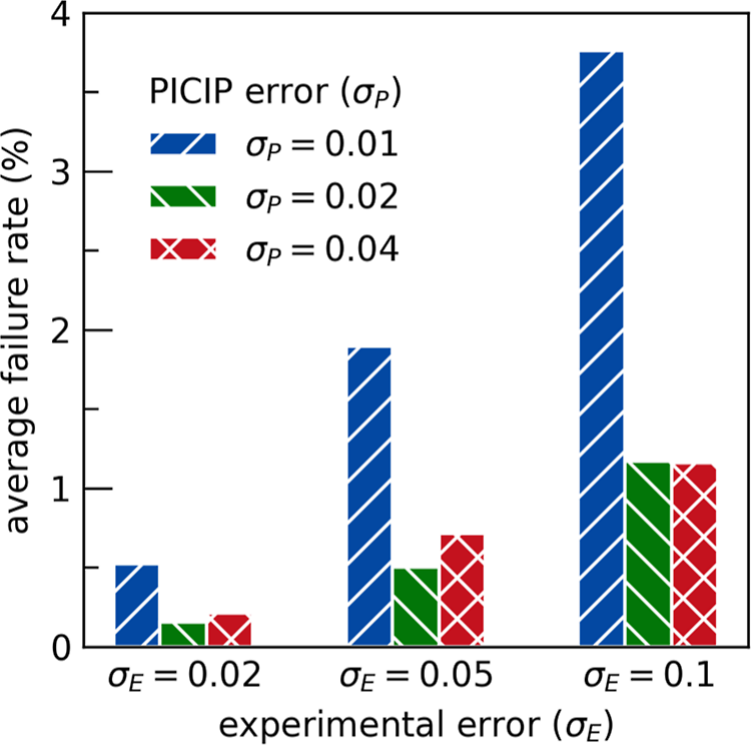
PICIP’s Failure Rate for each combination of experimental error (σ_E_) and PICIP error (σ_P_), averaged over all unknowns in all phase fields.

In situations where much of the experimental work can easily be run in parallel, it may be preferable to synthesize samples in batches rather than serially. For batch sizes 1, 3, and 5 the median Purity Score vs number of samples, averaged across all unknowns, for a PICIP error of 2 wt % (σ_P_ = 0.02), and an experimental error of 5 wt % (σ_E_ = 0.05) is examined (Figure 10). A median Purity Score of ∼90% is achieved after only two batches when the batch size is increased to five, compared to four samples generated in serial. Isolation of the unknown phase will therefore be more efficient for batch-wise synthesis under conditions where the time taken to prepare, fire, and analyze a batch of samples is much shorter than the time to prepare the same number of samples serially. This is likely to be the case when there is a long firing time or high-throughput synthesis methods are used. For batch suggestions, the mean of the probability distribution is always used, and further points are sampled randomly from the probability distribution. This method makes no attempt to spread samples out, neither in the same batch nor across multiple batches, and so it is likely that batch-wise selection can be improved further.

**Figure 10:**
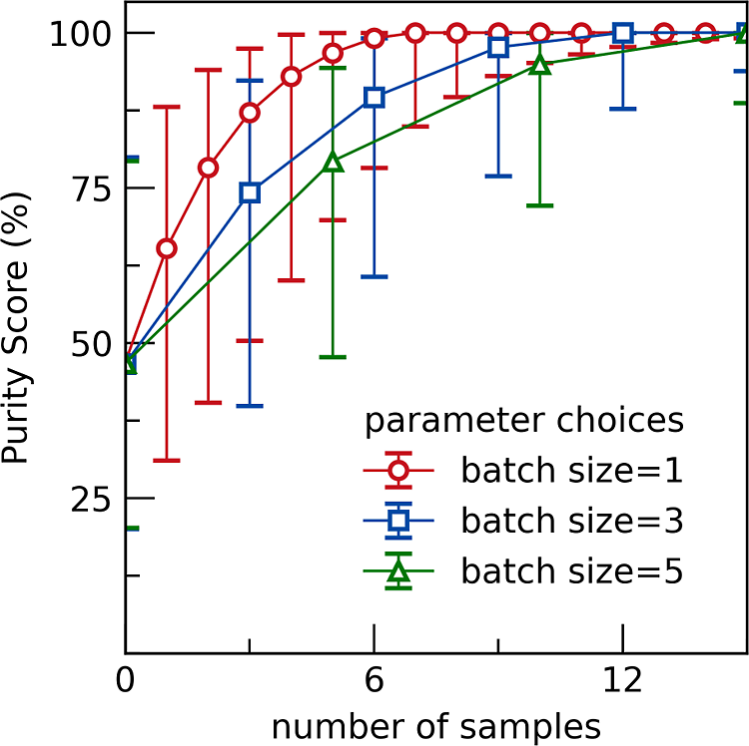
Effect of sampling in batches. Results shown are averaged across all unknown phases, the PICIP error is 2 wt % (σ_P_ = 0.02) and the experimental error is 5 wt % (σ_E_ = 0.05). The error bars show the 16th and 84th percentiles, respectively.

Throughout this report, the three phase fields investigated here all have two-dimensional representations. This decision was made to make a clear interpretable visual demonstration and convincingly demonstrate its effectiveness. The method which PICIP uses to construct its probability densities is applicable to any number of dimensions. While the size of the discrete grid which PICIP uses to represent the phase field grows exponentially with the number of elements, the density of points can be decreased to reduce the computational requirements. This has allowed PICIP to be applied to three-dimensional phase fields such as in,^13^ and in theory it is able to handle higher dimensions.

These results indicate that PICIP performs well within the provided simulation environment, however it is important to note that this environment relies on multiple assumptions and as such does not account for all sources of error that will be encountered in experimental isolation. Nevertheless, PICIP’s probabilistic construction is designed to make it a robust tool in the face of such additional sources of uncertainty. For example, the simulations assume that the composition of the sample (point *s*) is known exactly. This may not be the case due to measurement error when weighing the precursors, volatility of the precursors or products, and reactions with the atmosphere or crucible. Referring to Figure 3 these effects would result in the point *s*, the apex of the cone, being in a different position and the estimated direction extending in a different direction. This effect will be more pronounced the closer *s* is to the composition of the unknown, *u*. As long as the resulting estimated direction is still within 90° of the true direction to the unknown phase, then this effect can be accounted for by increasing the PICIP error. Within the simulation environment it is assumed that the estimated relative mass fractions will be normally distributed about their true value. This will not be the case in the presence of systematic errors. This effect can be mitigated by using a batch of samples in which the combinations of known phases are not all the same. It is likely that the systematic error for one combination of known phases will not be common across all samples containing different combinations of known phases.

## Conclusion

We present PICIP, a novel sampling algorithm for the isolation of unknown crystalline phases, alongside a computational simulation for evaluating its performance. These two parts are intentionally separate, with the aim of being able to compare the performance of future alternative sampling algorithms.

PICIP uses experimentally determined relative phase ratios to construct a probability density, which accounts for the estimated uncertainties on the phase ratios. This probability density estimates the composition of the unknown crystalline phase. An iterative sampling strategy then suggests subsequent compositions to sample, where the information from each new sampled composition is used to update the probability density for the next suggestions.

Throughout the isolation process, samples used to refine the probability density must contain the unknown phase, all other phases must be known, and it must be possible to estimate the relative ratios of these known phases. The prediction that PICIP makes is purely compositional, containing no information on the reaction conditions required to form the unknown phase from starting materials with the targeted average composition. However, the reaction conditions used to obtain samples for PICIP do not need to be constant. Furthermore, while it was necessary to assume thermodynamic equilibrium in the simulation environment, this is not a constraint on experimental samples.

PICIP may be used to suggest samples serially or batch-wise. Suggesting samples in batches leads to fewer batches, but requires more samples in total. Owing to its probabilistic construction, PICIP is capable of combining information from multiple sampled compositions to increase the accuracy of its suggestions. These factors make PICIP equally well-suited to autonomous, automated, or manual synthesis approaches, in either serial or batch mode.

We demonstrate PICIP is a useful tool at the laboratory bench scale, capable of isolating unknown compositions in phase fields with three or four different elements. Importantly, the method used to construct probability densities works independently of the dimensionality of the representation, which allows PICIP to suggest sample compositions in phase fields with an arbitrary number of elements. Given the drive toward materials with increasingly complex compositions, experimental tools such as PICIP will become increasingly beneficial.

The main hyper-parameter affecting PICIP’s performance is the PICIP error (σ_P_), the assumed standard deviation on the relative mass ratios returned by Rietveld analysis. The optimal choice of PICIP error is dependent both on the geometry of the phase field and the amount of error on the estimated relative mass fractions of known crystalline phases. A PICIP error of 2 wt % (σ_P_ = 0.02) is a good starting value for any investigation. Reducing the PICIP error to 1 wt % may help speed up the isolation when the region of the phase diagram forming the unknown phase is small, or when the experimental error is expected to be small. Where the PICIP error underestimates the experimental error, it is more likely that PICIP will fail to produce a nonzero probability distribution and hence identify a direction for further synthesis (Figure 9). In these circumstances, the PICIP error should be increased (e.g., to 4 wt %) until a reasonable probability distribution is obtained.

Across three chemically distinct phase fields investigated here, computational simulations show sample compositions suggested by PICIP consistently converge to the unknown phase being sought. When using batch sizes of one and an experimental error of 10 wt % (σ_E_ = 0.1), a median of six samples is required to obtain at least 90% purity of the unknown phase.

In addition to its demonstrated use within a manual materials discovery approach,^13^ PICIP has a potential role within a materials discovery workflow incorporating a level of automation. PICIP can provide a feedback loop for any procedure which generates reacted samples within a phase field and is able to analyze both whether there is an unknown material present and what the weight percentages of known materials are within some known or estimated error. The current study demonstrates that a relatively small number of cycles of this PICIP informed feedback loop should be required to isolate the identified unknown phase.

## Supplementary Material



## Data Availability

Data and Software Availability: All code used to run simulations and produce the data, along with the required libraries are available at https://github.com/lrcfmd/PICIP.

## Notes

Detailed results for each simulated target unknown phase and additional details of the methods (PDF)
