# Supplementary material for: Probabilistic Isolation of Crystalline Inorganic Phases
Source: J Chem Inf Model. 2025 Dec 4;65(24):13226–37. doi: 10.1021/acs.jcim.5c02256 (PMC12728945; doi:10.1021/acs.jcim.5c02256)
Supplement: Supplementary file 1 [file ci5c02256_si_001.pdf]

Probabilistic Isolation of Crystalline Inorganic Phases

Daniel Ritchie[1,2], Michael W. Gaultois[1,2], Vladimir V. Gusev[1,3], Vitaliy Kurlin[1,3], Matthew J. Rosseinsky[1,2], Matthew S. Dyer[1,2]

[1]Leverhulme Research Centre for Functional Materials Design, Materials Innovation Factory, 51 Oxford St

[2]Department of Chemistry, University of Liverpool, Crown Street, Liverpool, L69 7ZD, UK

[3]Department of Computer Science, University of Liverpool, Ashton Building, Liverpool, L69 3DR, UK

November 6, 2025

Contents

1 Mg-Al-Cu Phase Field **S2**
1.1 $MgAl_2Cu$ S2
1.2 $Mg_2Al_5Cu_6$ S5
1.3 $Mg_3(AlCu_2)_2$ S8

2 Li^{1+}-Al^{3+}-B^{3+}-O^{2-} **S11**
2.1 $Li_{2.46}Al_{0.18}BO_3$ S11
2.2 $LiAl_7B_4O_{17}$ S14
2.3 $Li_2AlB_5O_{10}$ S17
2.4 Li_2AlBO_4 . . . . . S20
2.5 $Li_3AlB_2O_6$ S23
2.6 $LiAlB_2O_5$ S26

3 Mg^{2+}-B^{3+}-O^{2-}-F^{1-} **S29**
3.1 $Mg_3B(OF)_3$ S29
3.2 $Mg_5B_3O_9F$ S32

4 Mathematical representation of phase field **S35**

5 Calculating direction from relative mass fractions **S35**

6 Allowing for experimental uncertainty **S35**

7 PICIP error **S36**

8 Edge cases in model simulation **S36**

9 Discretisation schemes **S37**

10 Choice of hyperparameters **S37**

1 Mg-Al-Cu Phase Field

1.1 $MgAl_2Cu$

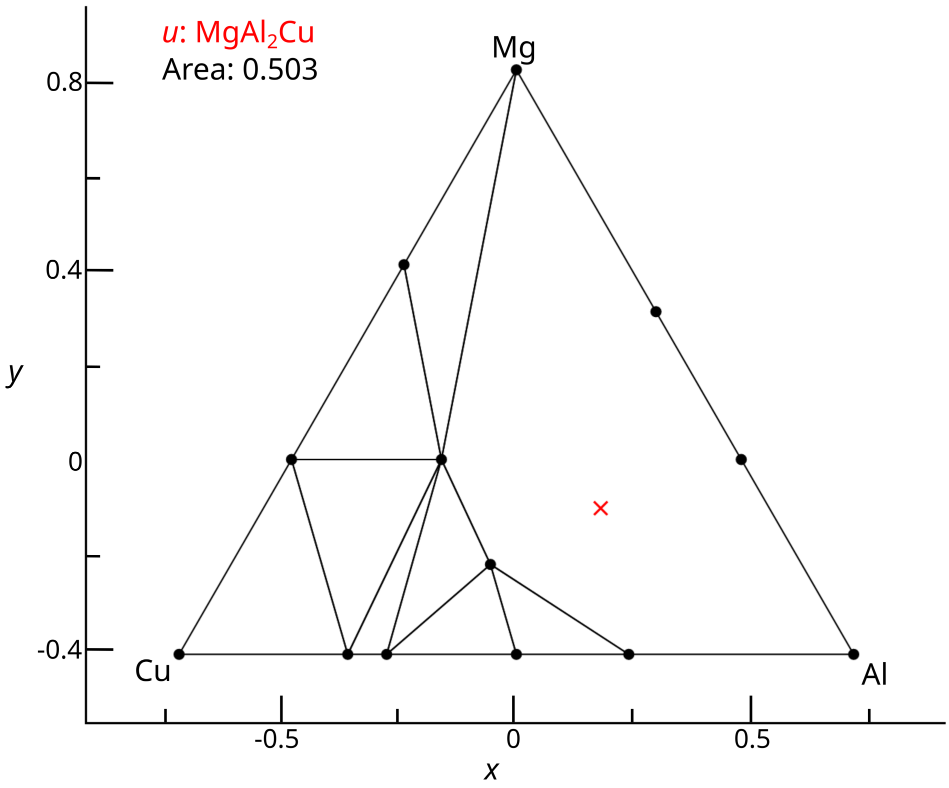

Figure S1: The Mg-Al-Cu phase field, assuming the $MgAl_2$ phase is unknown. Area describes the relative area of the region which would form $MgAl_2Cu$ at thermodynamic equilibrium.

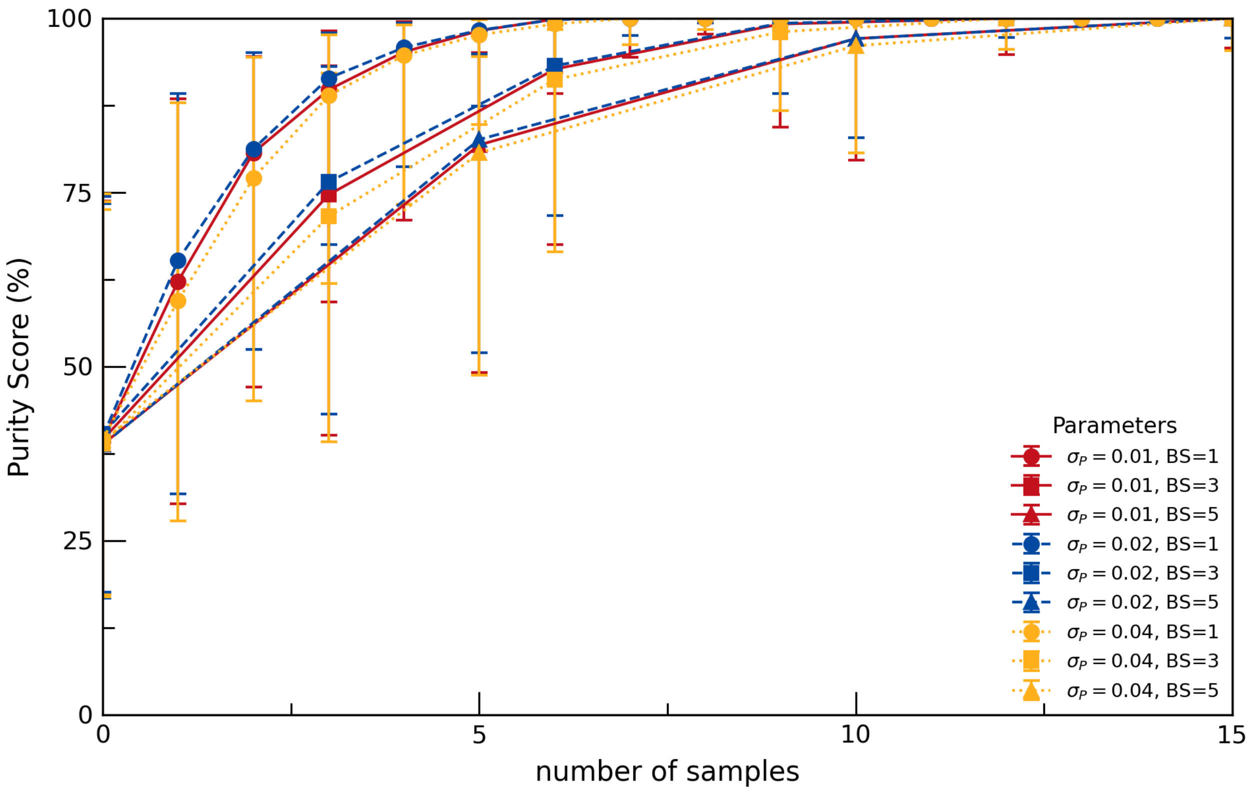

Figure S2: Median Purity Score vs number of samples for the $MgAl_2Cu$ phase and an experimental error of 2 wt% (σ_E=0.02). Results are shown for all combinations of PICIP error (σ_P) and Batch size (BS). The error bars show the 16th and 84th percentiles respectively.

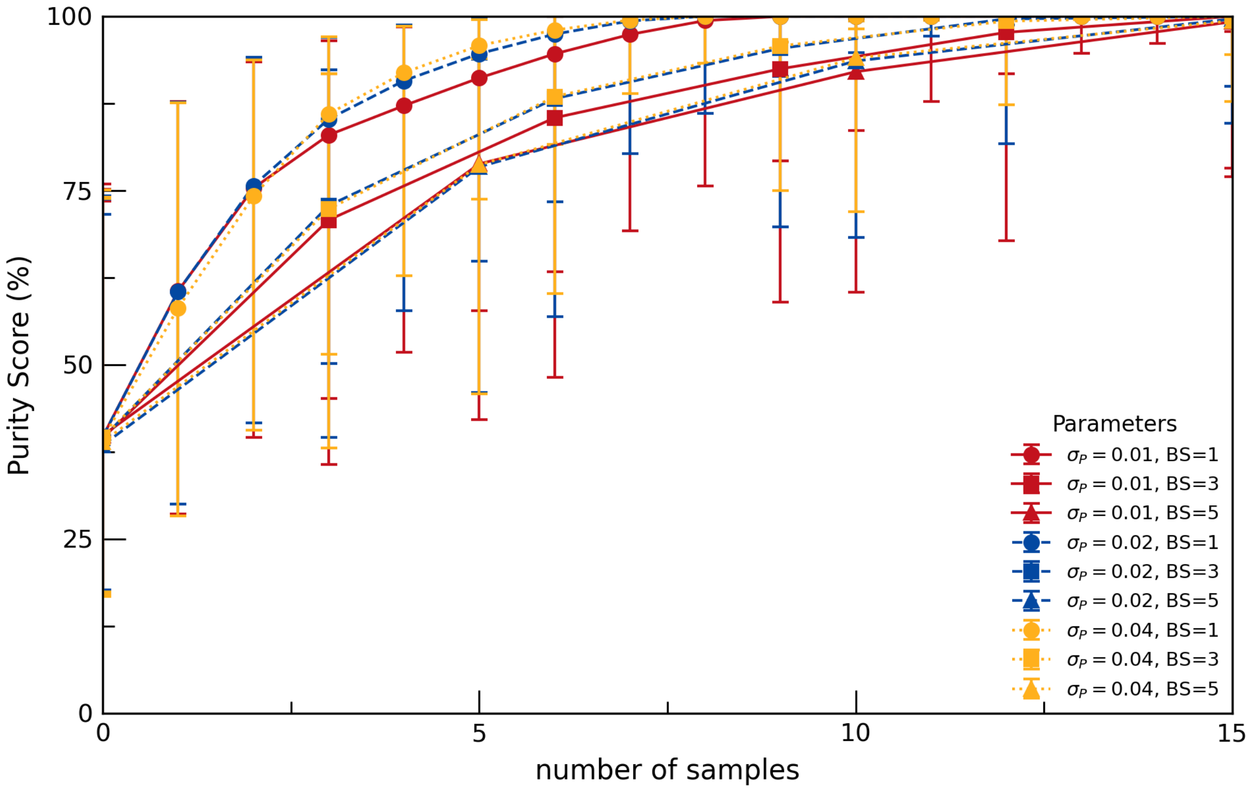

Figure S3: Median Purity Score vs number of samples for the $MgAl_2Cu$ phase and an experimental error of 5 wt% (σ_E=0.05). Results are shown for all combinations of PICIP error (σ_P) and Batch size (BS). The error bars show the 16th and 84th percentiles respectively.

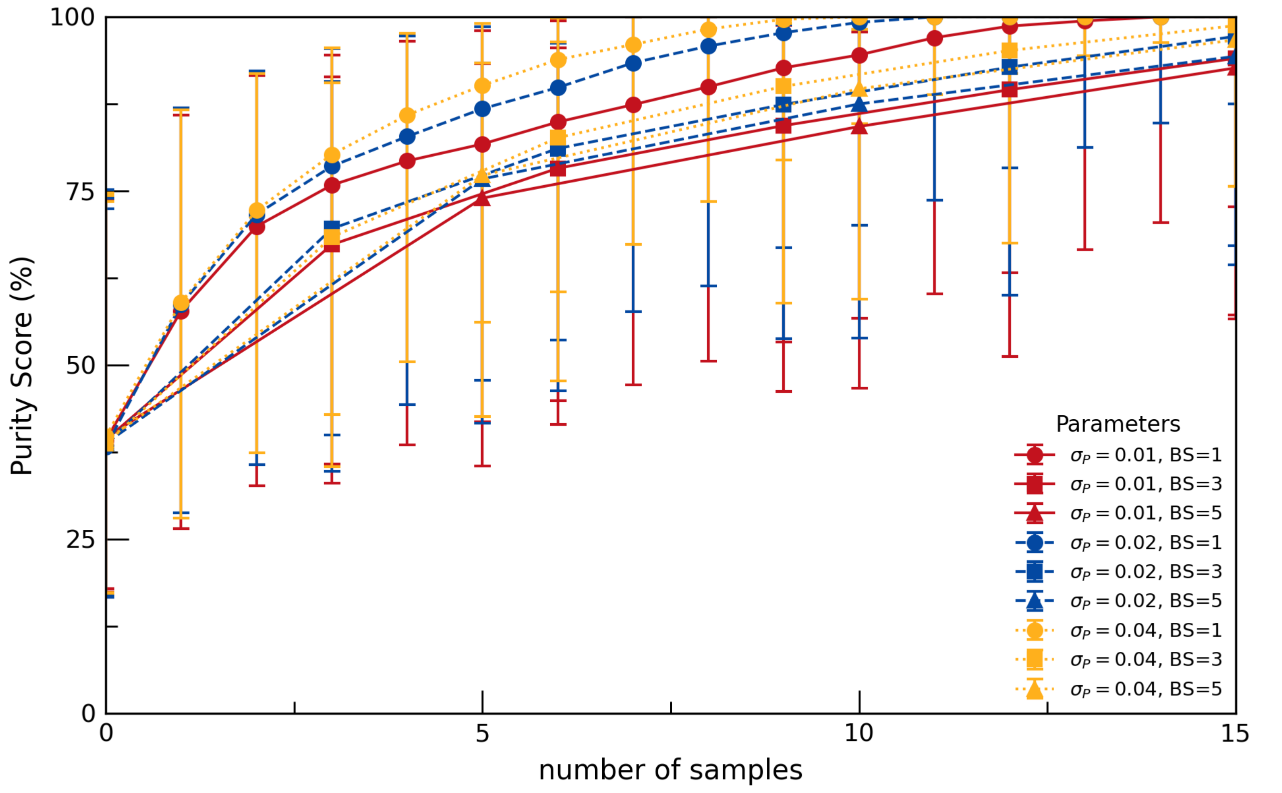

Figure S4: Median Purity Score vs number of samples for the $MgAl_2Cu$ phase and an experimental error of 10 wt% (σ_E=0.1). Results are shown for all combinations of PICIP error (σ_P) and Batch size (BS). The error bars show the 16th and 84th percentiles respectively.

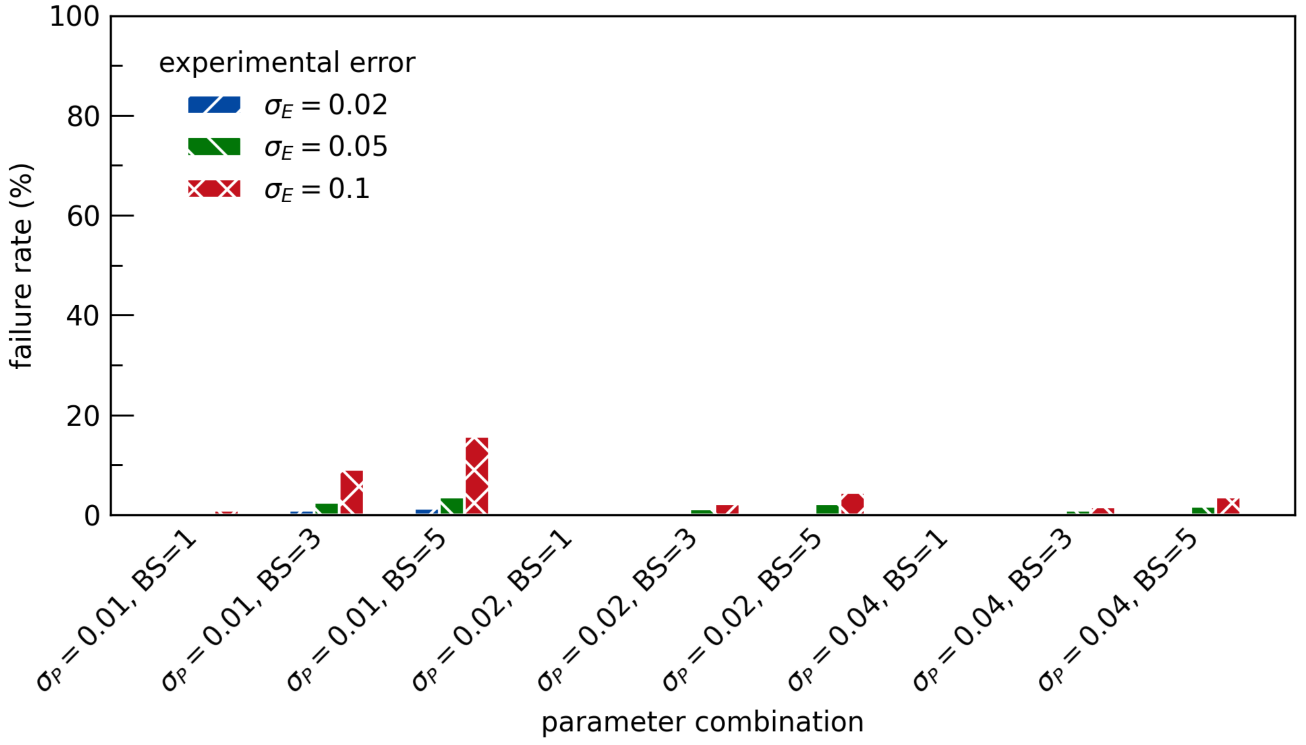

Figure S5: PICIP's failure rate for the $MgAl_2Cu$ phase, for all combinations of PICIP error (σ_P), Experimental error (σ_E), and Batch size (BS)

1.2 $Mg_2Al_5Cu_6$

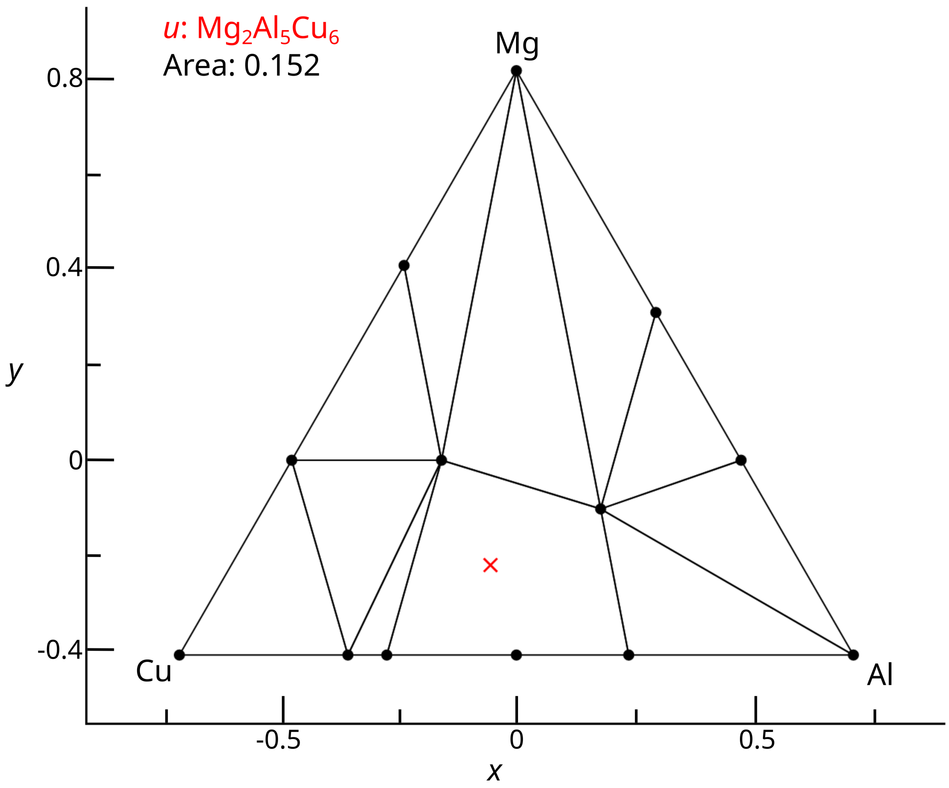

Figure S6: The Mg-Al-Cu phase field, assuming the $Mg_2Al_5Cu_6$ phase is unknown. Area describes the relative area of the region which would form $Mg_2Al_5Cu_6$ at thermodynamic equilibrium.

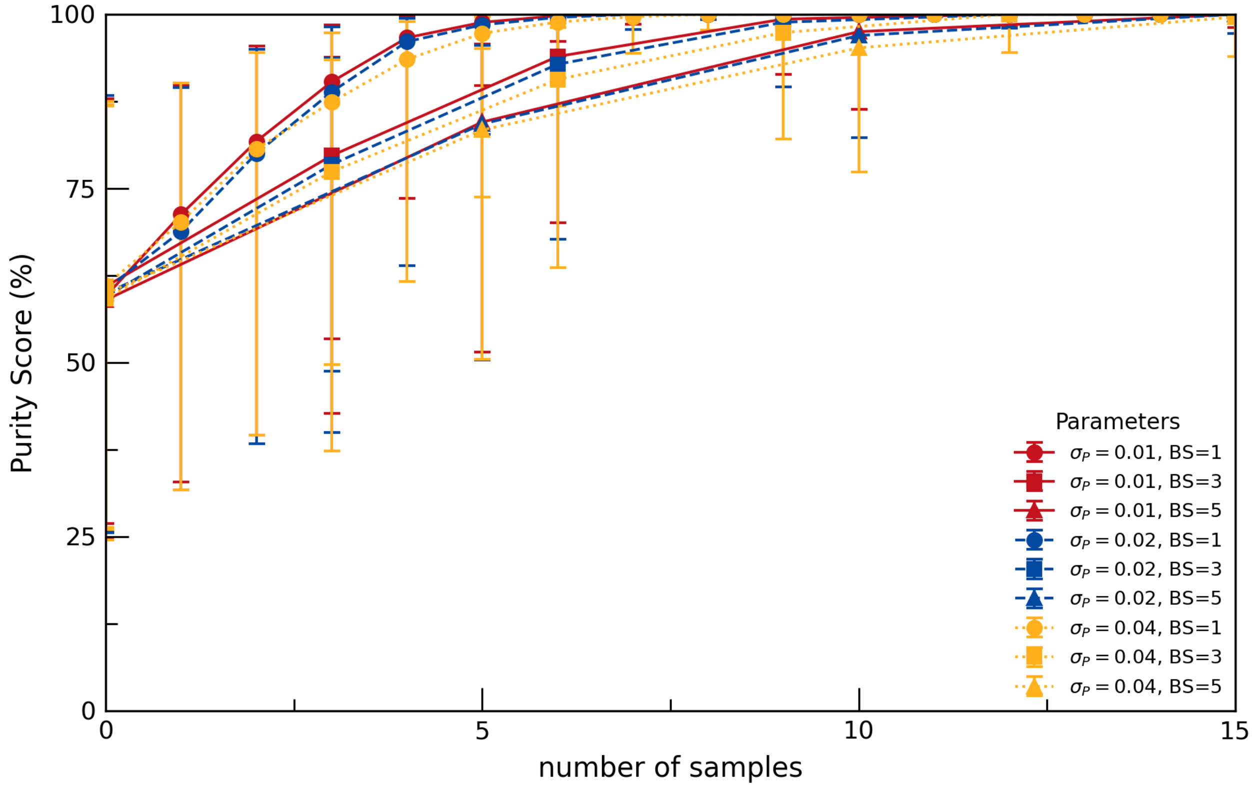

Figure S7: Median Purity Score vs number of samples for the $Mg_2Al_5Cu_6$ phase and an experimental error of 2 wt% (σ_E=0.02). Results are shown for all combinations of PICIP error (σ_P) and Batch size (BS). The error bars show the 16th and 84th percentiles respectively.

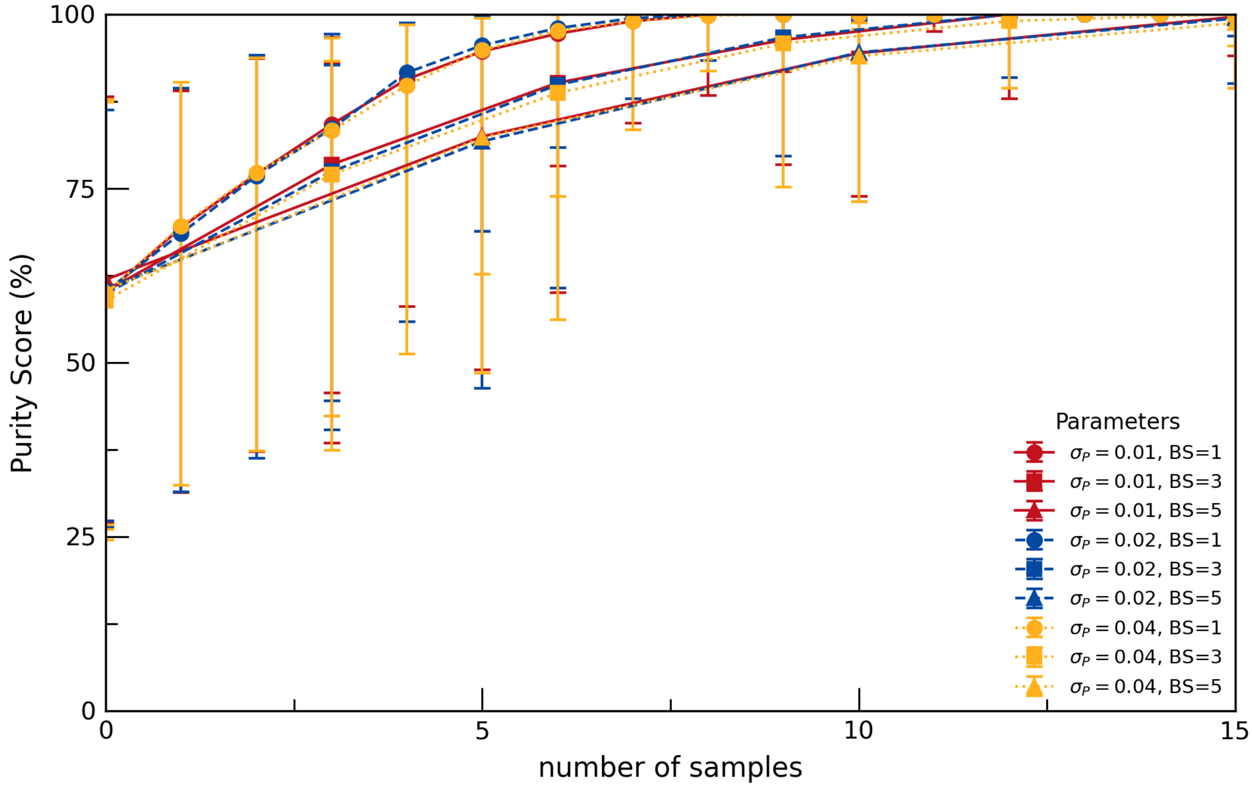

Figure S8: Median Purity Score vs number of samples for the $Mg_2Al_5Cu_6$ phase and an experimental error of 5 wt% (σ_E=0.05). Results are shown for all combinations of PICIP error (σ_P) and Batch size (BS). The error bars show the 16th and 84th percentiles respectively.

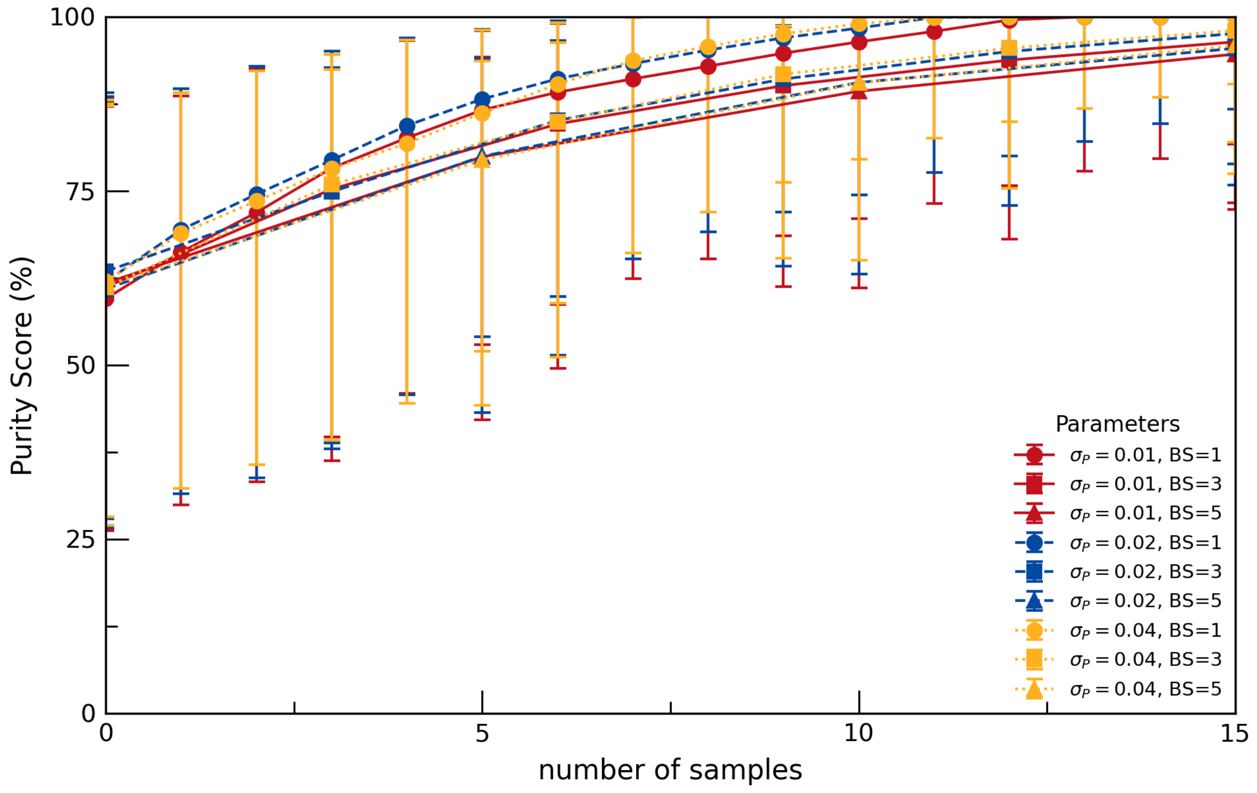

Figure S9: Median Purity Score vs number of samples for the $Mg_2Al_5Cu_6$ phase and an experimental error of 10 wt% (σ_E=0.1). Results are shown for all combinations of PICIP error (σ_P) and Batch size (BS). The error bars show the 16th and 84th percentiles respectively.

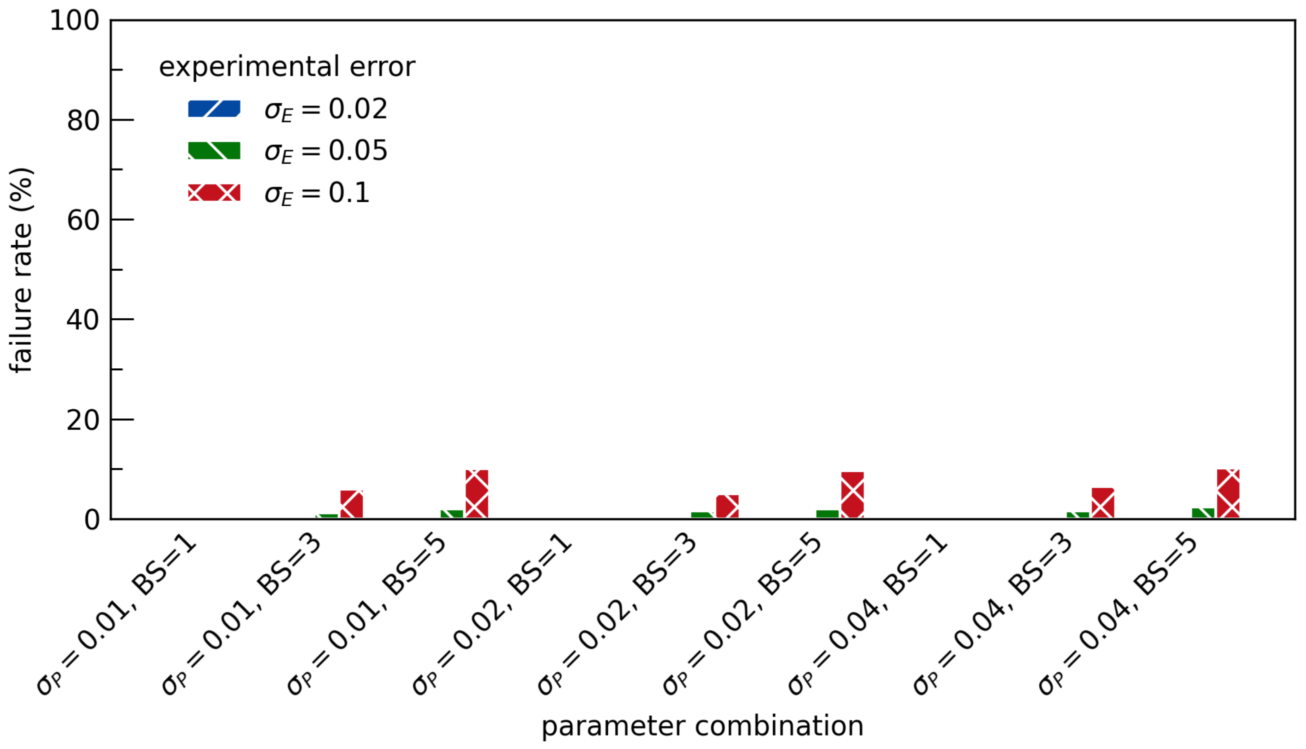

Figure S10: PICIP's failure rate for the $Mg_2Al_5Cu_6$ phase, for all combinations of PICIP error (σ_P), Experimental error (σ_E), and Batch size (BS)

1.3 $Mg_3(AlCu_2)_2$

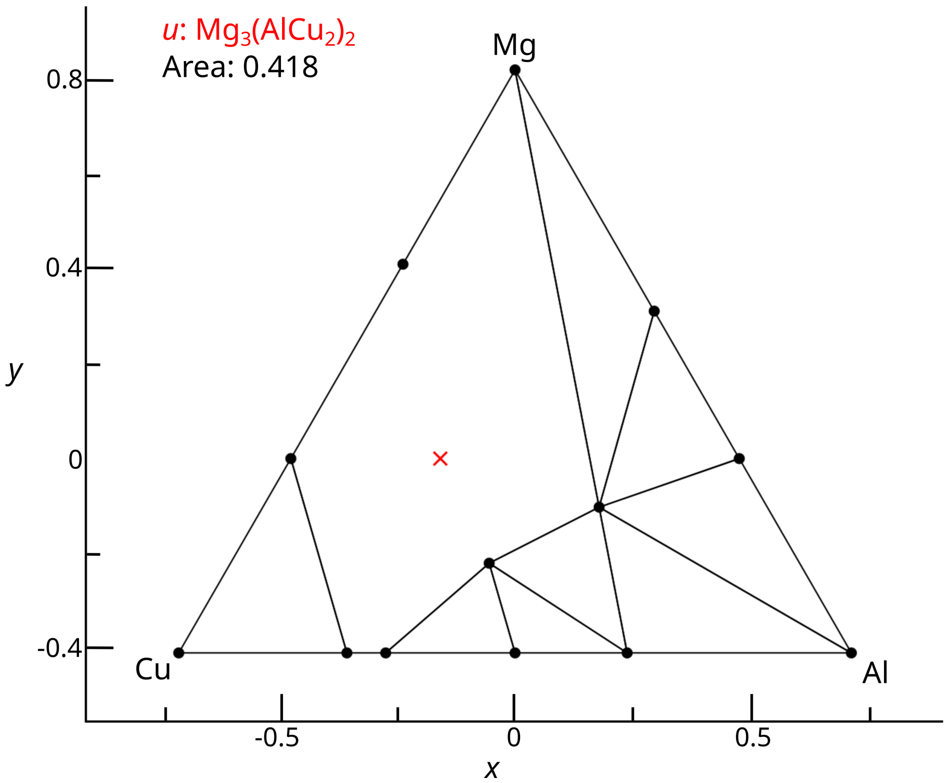

Figure S11: The Mg-Al-Cu phase field, assuming the $Mg_3(AlCu_2)_2$ phase is unknown. Area describes the relative area of the region which would form $Mg_3(AlCu_2)_2$ at thermodynamic equilibrium.

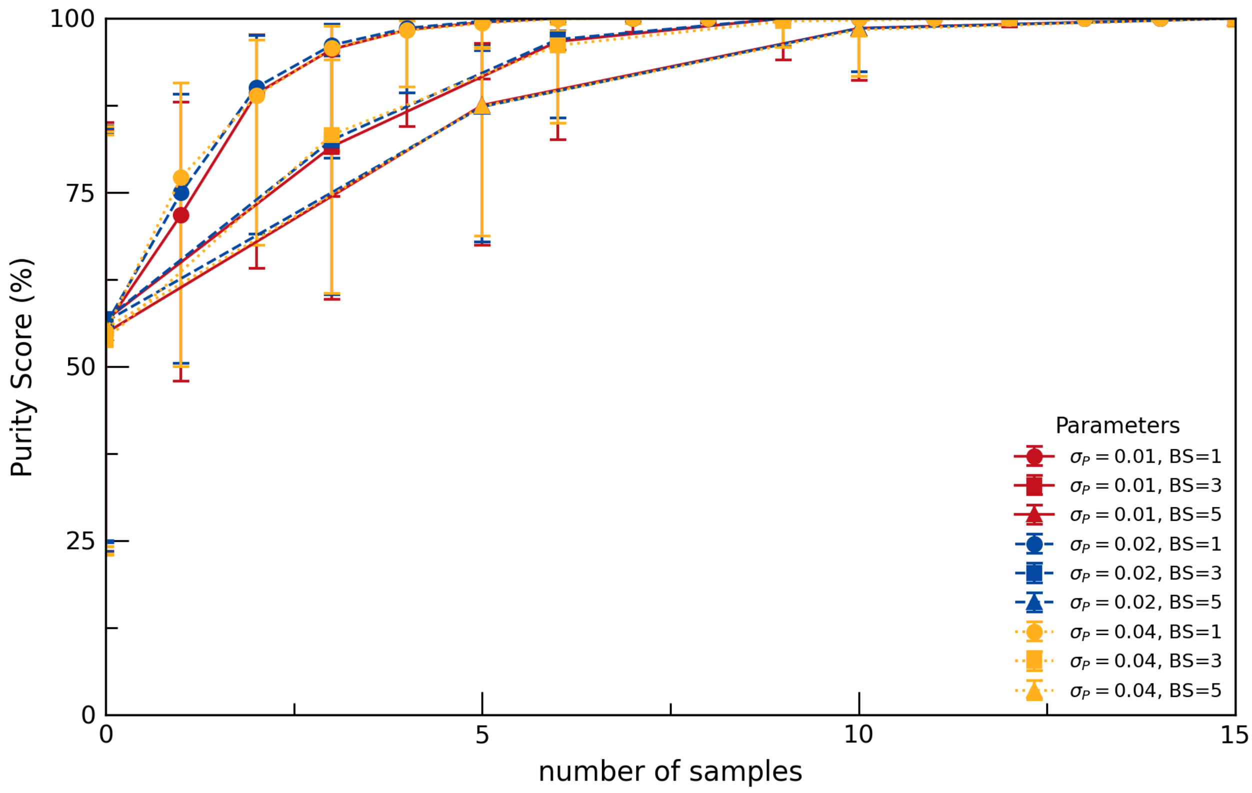

Figure S12: Median Purity Score vs number of samples for the $Mg_3(AlCu_2)_2$ phase and an experimental error of 2 wt% (σ_E=0.02). Results are shown for all combinations of PICIP error (σ_P) and Batch size (BS). The error bars show the 16th and 84th percentiles respectively.

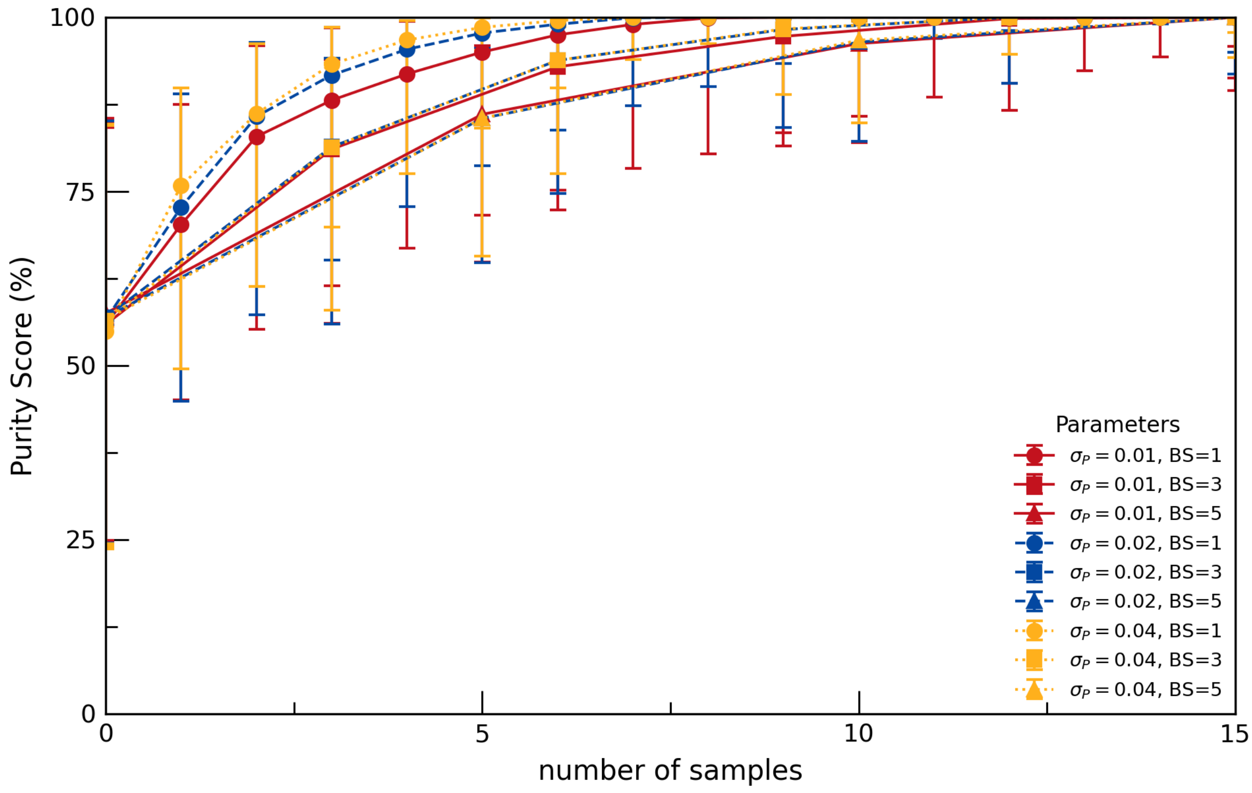

Figure S13: Median Purity Score vs number of samples for the $Mg_3(AlCu_2)_2$ phase and an experimental error of 5 wt% (σ_E=0.05). Results are shown for all combinations of PICIP error (σ_P) and Batch size (BS). The error bars show the 16th and 84th percentiles respectively.

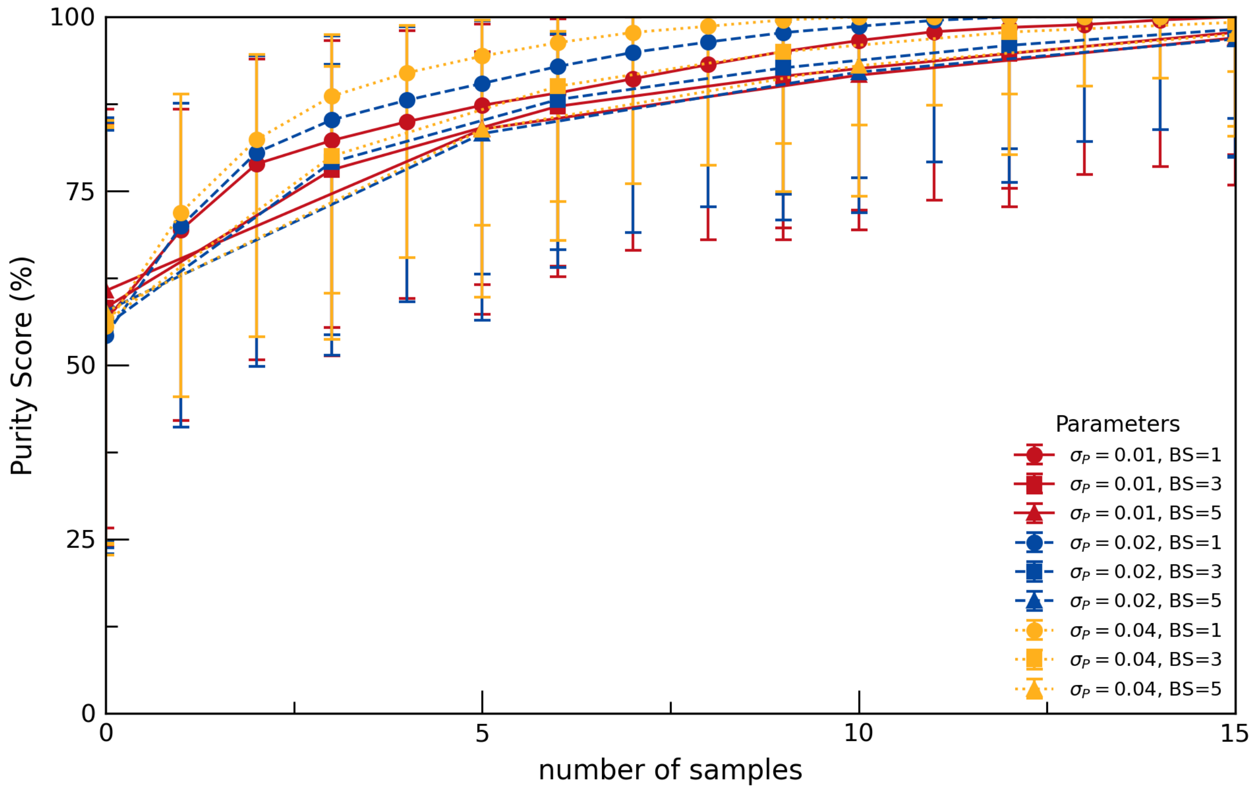

Figure S14: Median Purity Score vs number of samples for the $Mg_3(AlCu_2)_2$ phase and an experimental error of 10 wt% (σ_E=0.1). Results are shown for all combinations of PICIP error (σ_P) and Batch size (BS). The error bars show the 16th and 84th percentiles respectively.

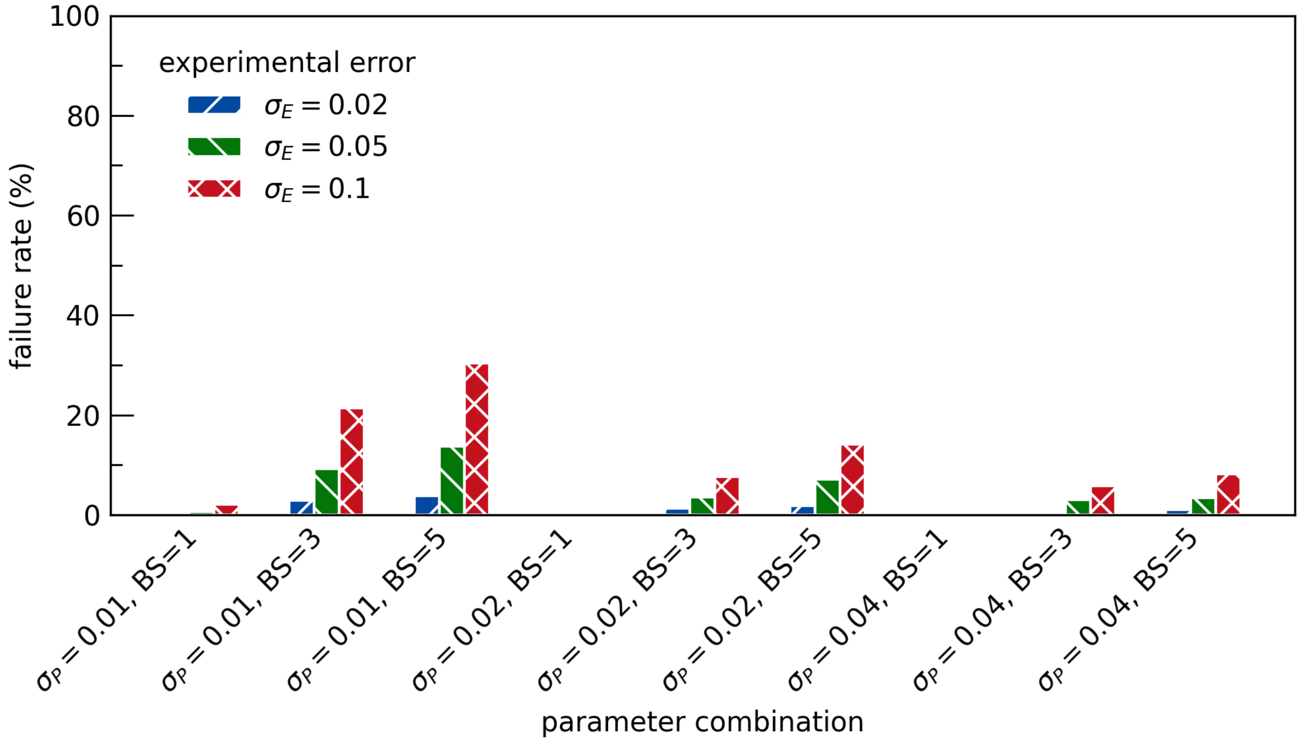

Figure S15: PICIP's failure rate for the $Mg_3(AlCu_2)_2$ phase, for all combinations of PICIP error (σ_P), Experimental error (σ_E), and Batch size (BS)

2 Li^{1+}-Al^{3+}-B^{3+}-O^{2-}

2.1 $Li_{2.46}Al_{0.18}BO_3$

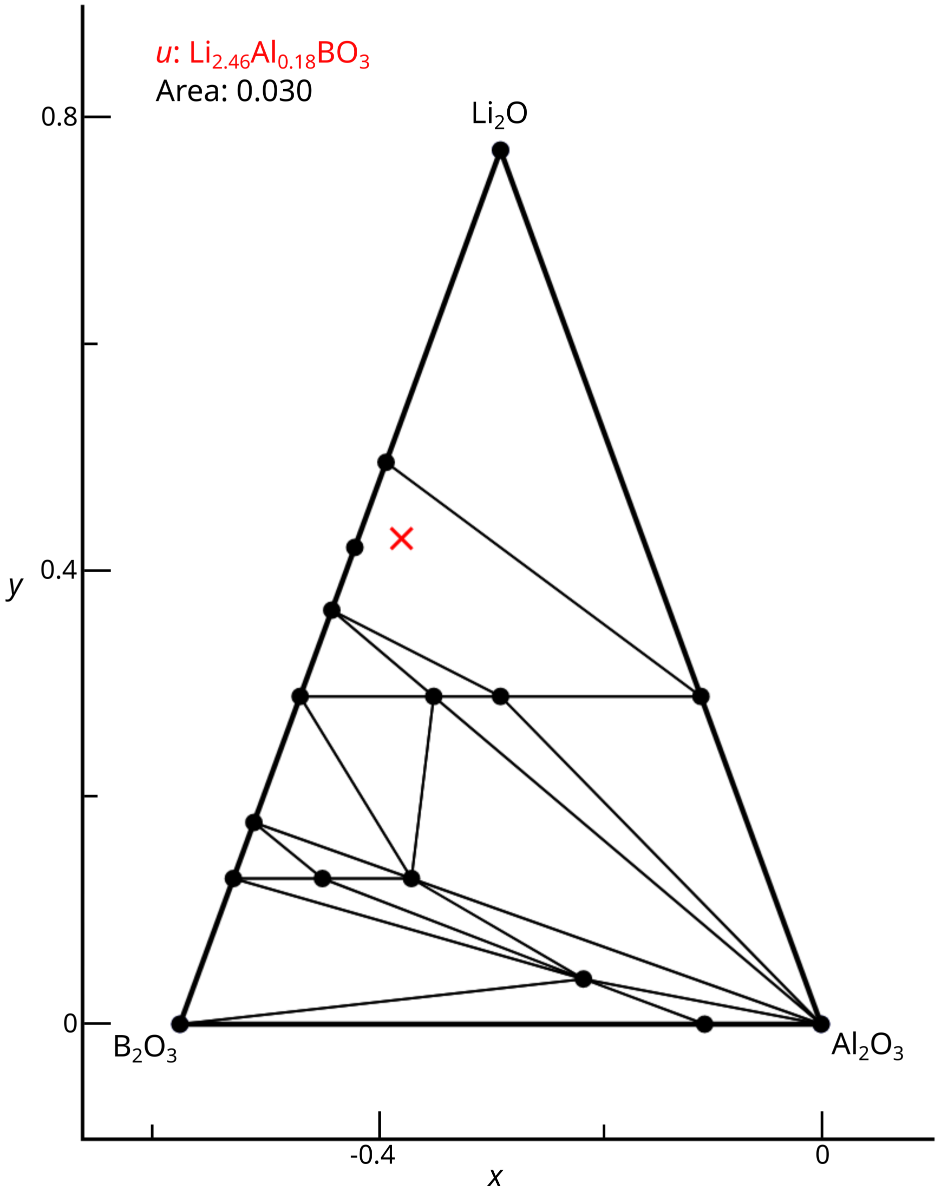

Figure S16: The Li^{1+}-Al^{3+}-B^{3+}-O^{2-} phase field, assuming the $Li_{2.46}Al_{0.18}BO_3$ phase is unknown. Area describes the relative area of the region which would form $Li_{2.46}Al_{0.18}BO_3$ at thermodynamic equilibrium.

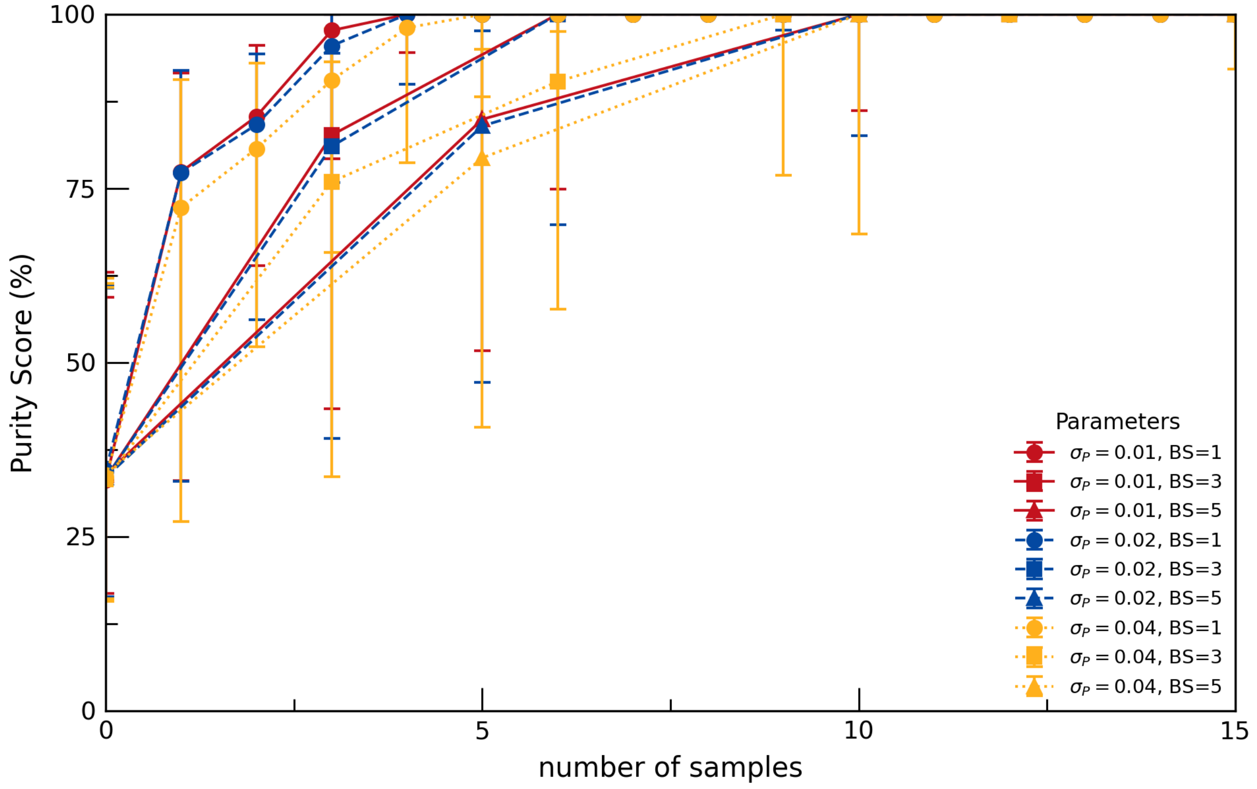

Figure S17: Median Purity Score vs number of samples for the $Li_{2.46}Al_{0.18}BO_3$ phase and an experimental error of 2 wt% (σ_E=0.02). Results are shown for all combinations of PICIP error (σ_P) and Batch size (BS). The error bars show the 16th and 84th percentiles respectively.

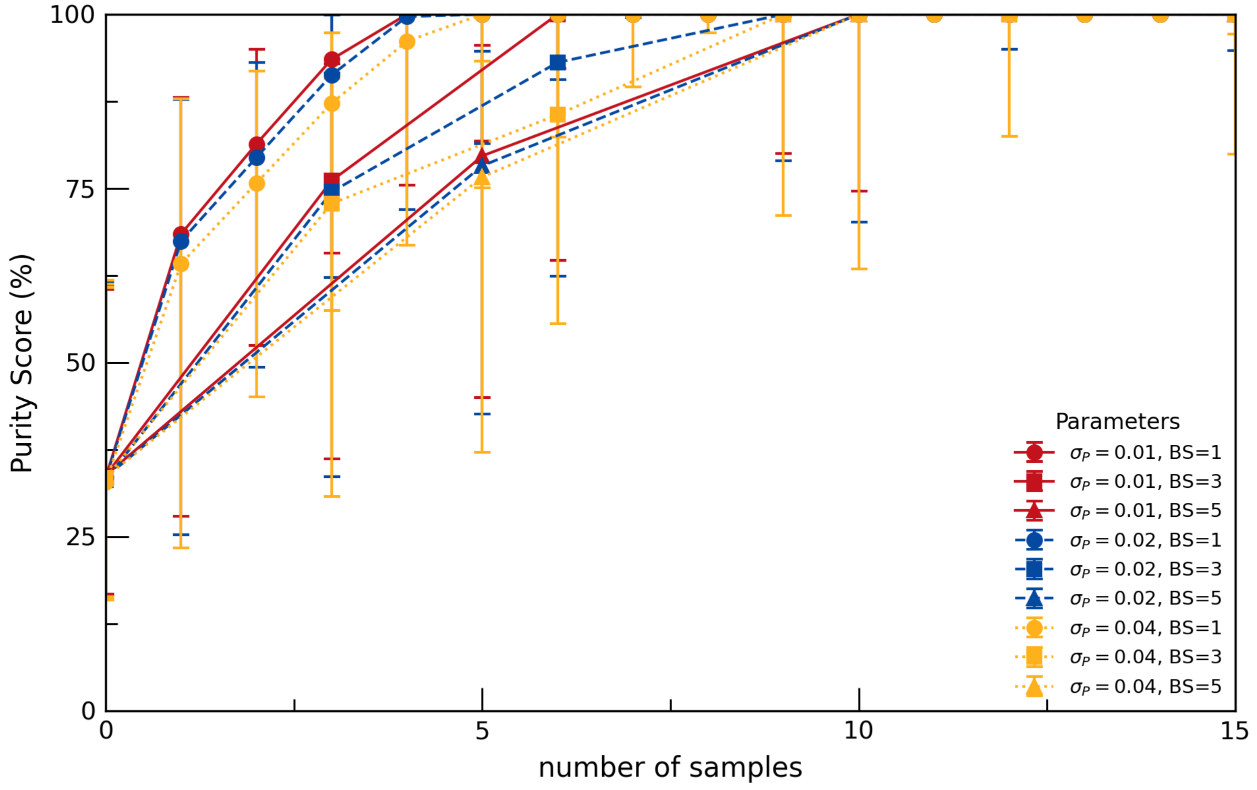

Figure S18: Median Purity Score vs number of samples for the $Li_{2.46}Al_{0.18}BO_3$ phase and an experimental error of 5 wt% (σ_E=0.05). Results are shown for all combinations of PICIP error (σ_P) and Batch size (BS). The error bars show the 16th and 84th percentiles respectively.

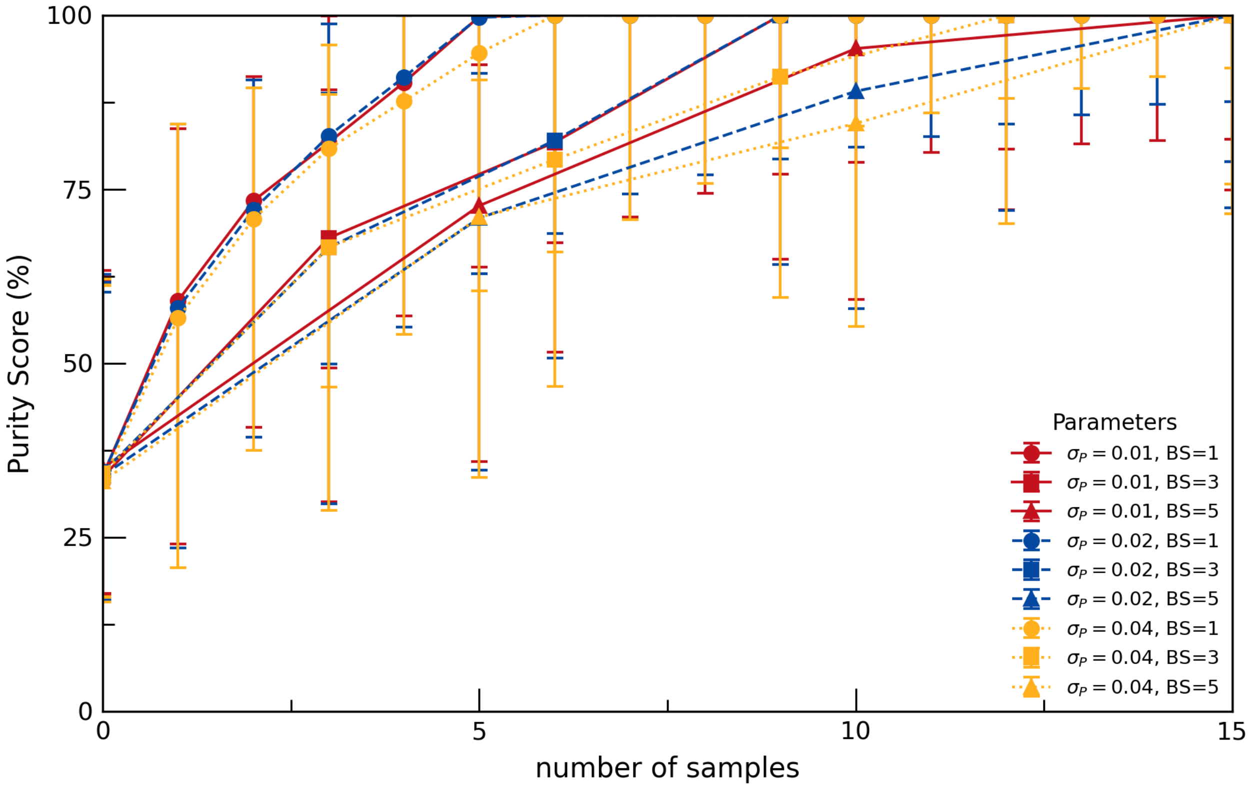

Figure S19: Median Purity Score vs number of samples for the $Li_{2.46}Al_{0.18}BO_3$ phase and an experimental error of 10 wt% (σ_E=0.1). Results are shown for all combinations of PICIP error (σ_P) and Batch size (BS). The error bars show the 16th and 84th percentiles respectively.

Experiment ID

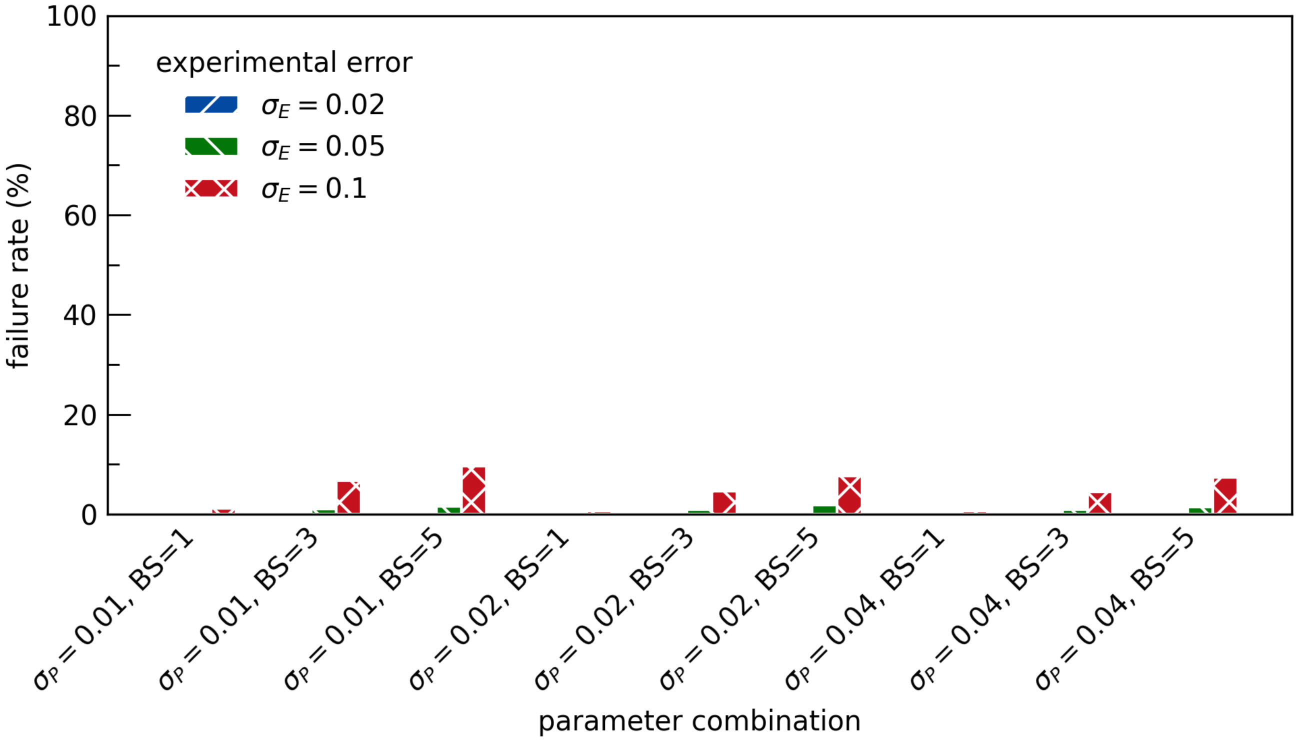

Figure S20: PICIP's failure rate for the $Li_{2.46}Al_{0.18}BO_3$ phase, for all combinations of PICIP error (σ_P), Experimental error (σ_E), and Batch size (BS)

2.2 $LiAl_7B_4O_{17}$

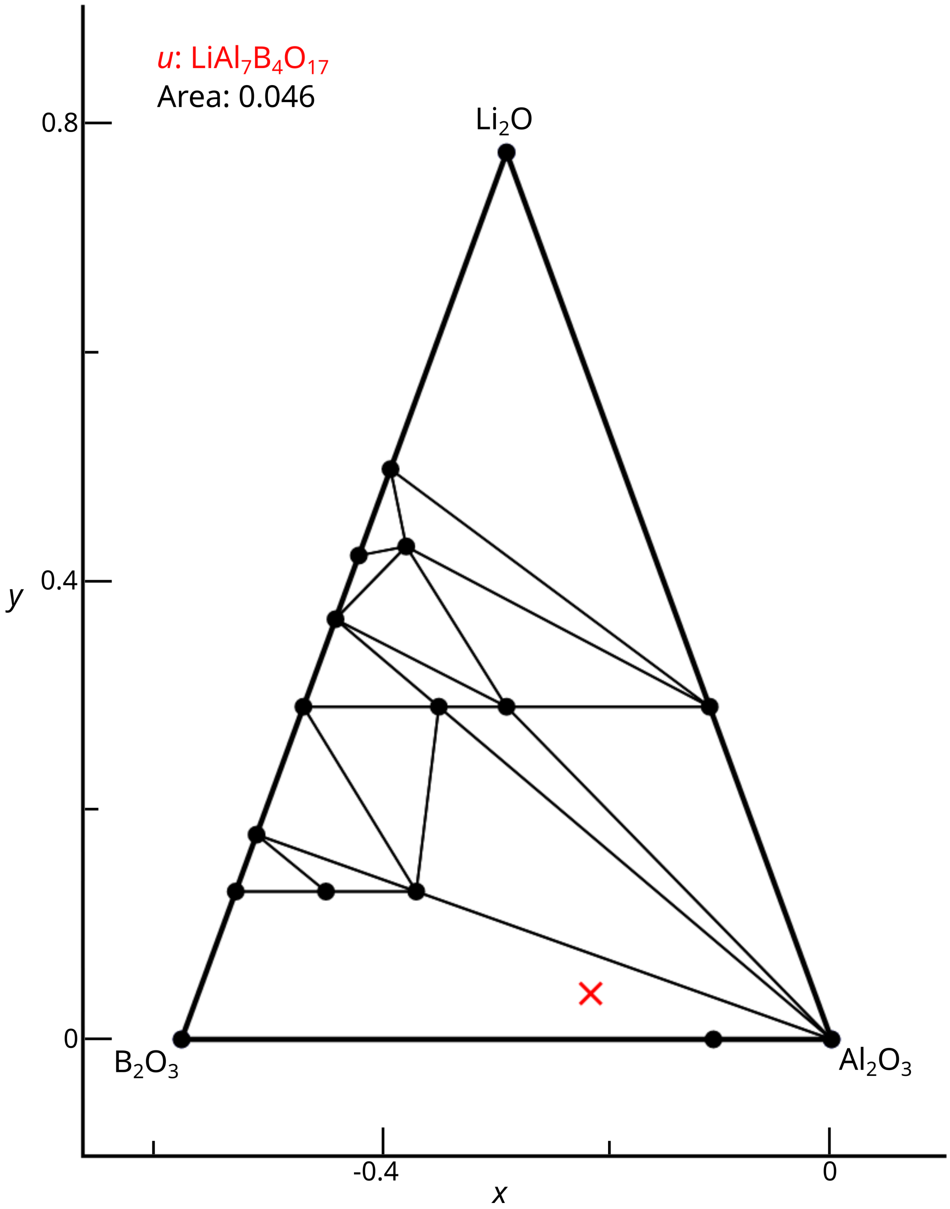

Figure S21: The Li^{1+}-Al^{3+}-B^{3+}-O^{2-} phase field, assuming the $LiAl_7B_4O_{17}$ phase is unknown. Area describes the relative area of the region which would form $LiAl_7B_4O_{17}$ at thermodynamic equilibrium.

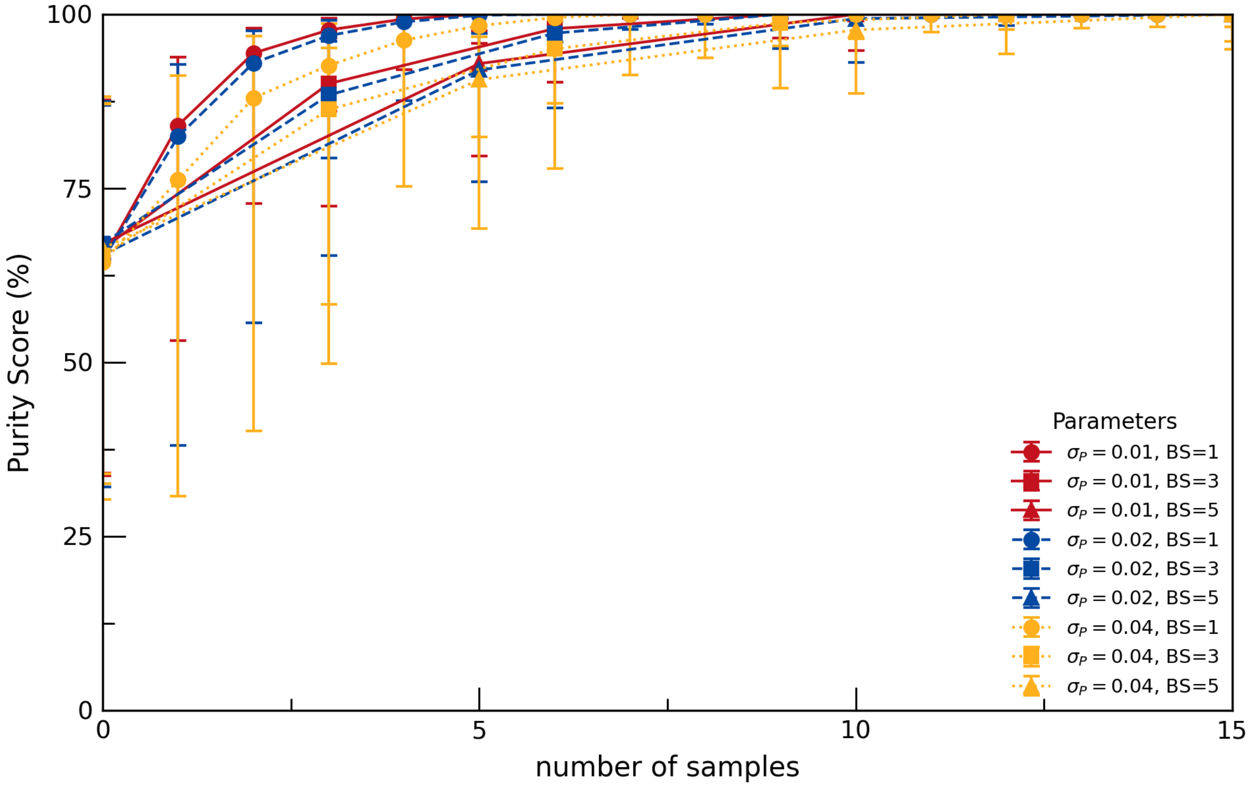

Figure S22: Median Purity Score vs number of samples for the $LiAl_7B_4O_{17}$ phase and an experimental error of 2 wt% (σ_E=0.02). Results are shown for all combinations of PICIP error (σ_P) and Batch size (BS). The error bars show the 16th and 84th percentiles respectively.

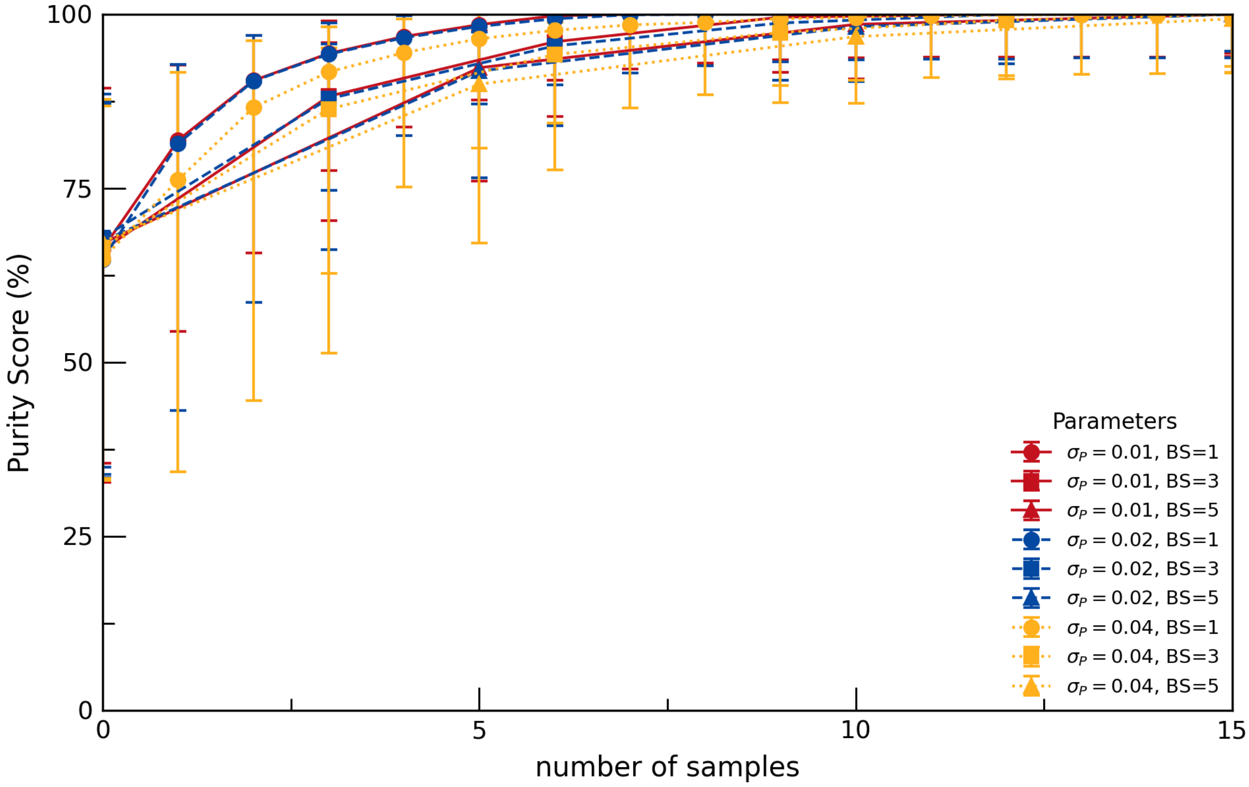

Figure S23: Median Purity Score vs number of samples for the $LiAl_7B_4O_{17}$ phase and an experimental error of 5 wt% (σ_E=0.05). Results are shown for all combinations of PICIP error (σ_P) and Batch size (BS). The error bars show the 16th and 84th percentiles respectively.

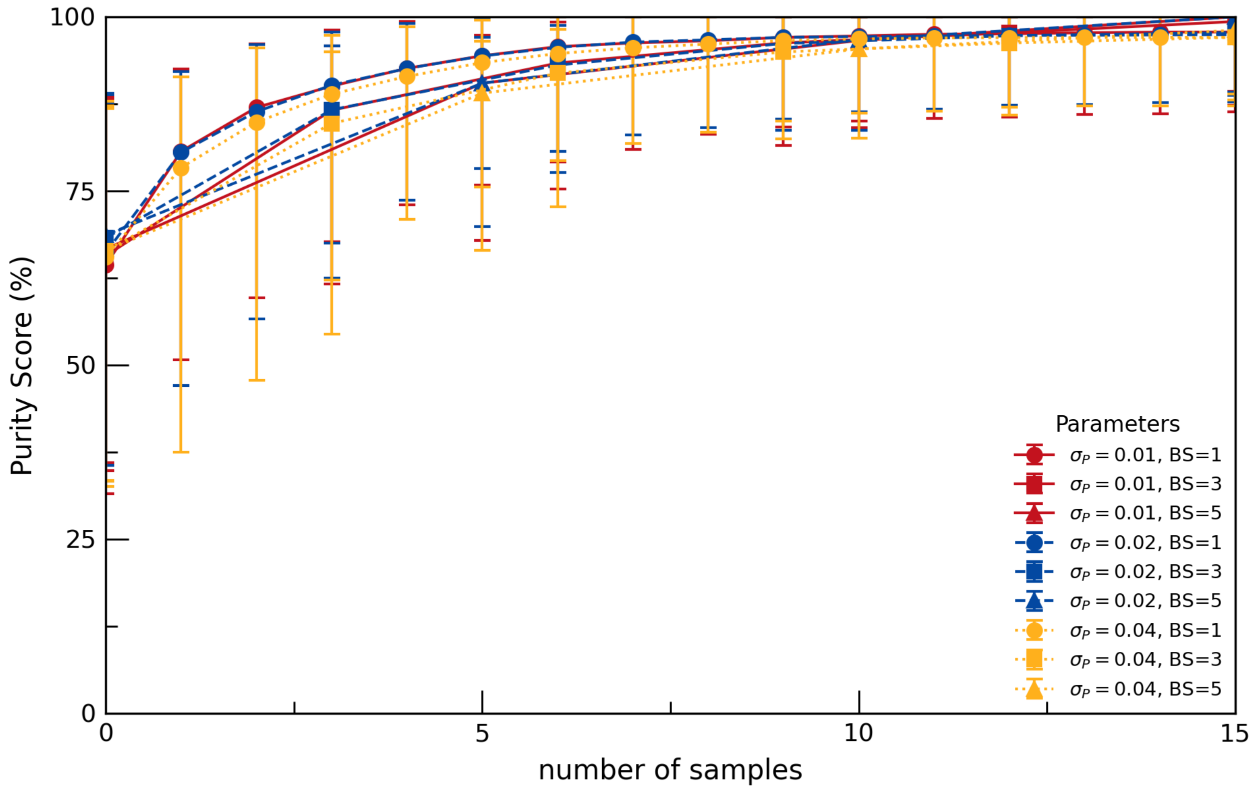

Figure S24: Median Purity Score vs number of samples for the $LiAl_7B_4O_{17}$ phase and an experimental error of 10 wt% (σ_E=0.1). Results are shown for all combinations of PICIP error (σ_P) and Batch size (BS). The error bars show the 16th and 84th percentiles respectively.

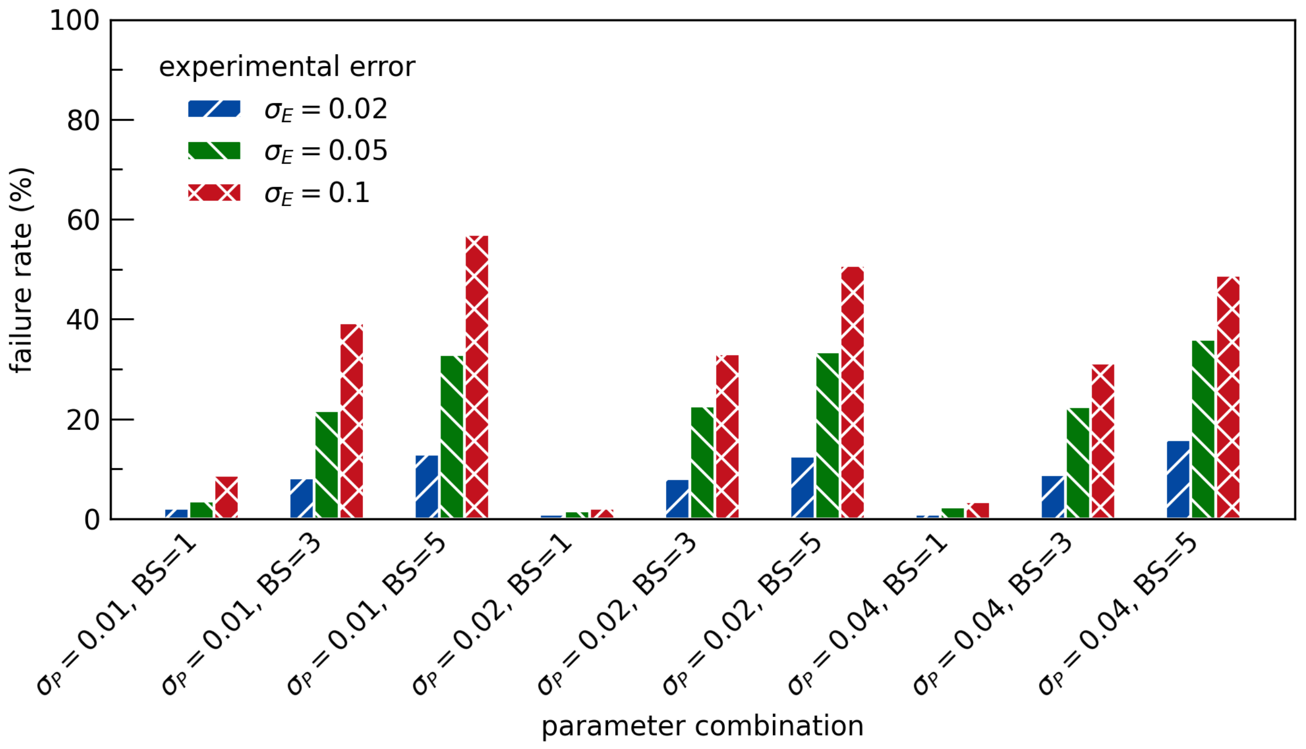

Figure S25: PICIP's failure rate for the $LiAl_7B_4O_{17}$ phase, for all combinations of PICIP error (σ_P), Experimental error (σ_E), and Batch size (BS)

2.3 $Li_2AlB_5O_{10}$

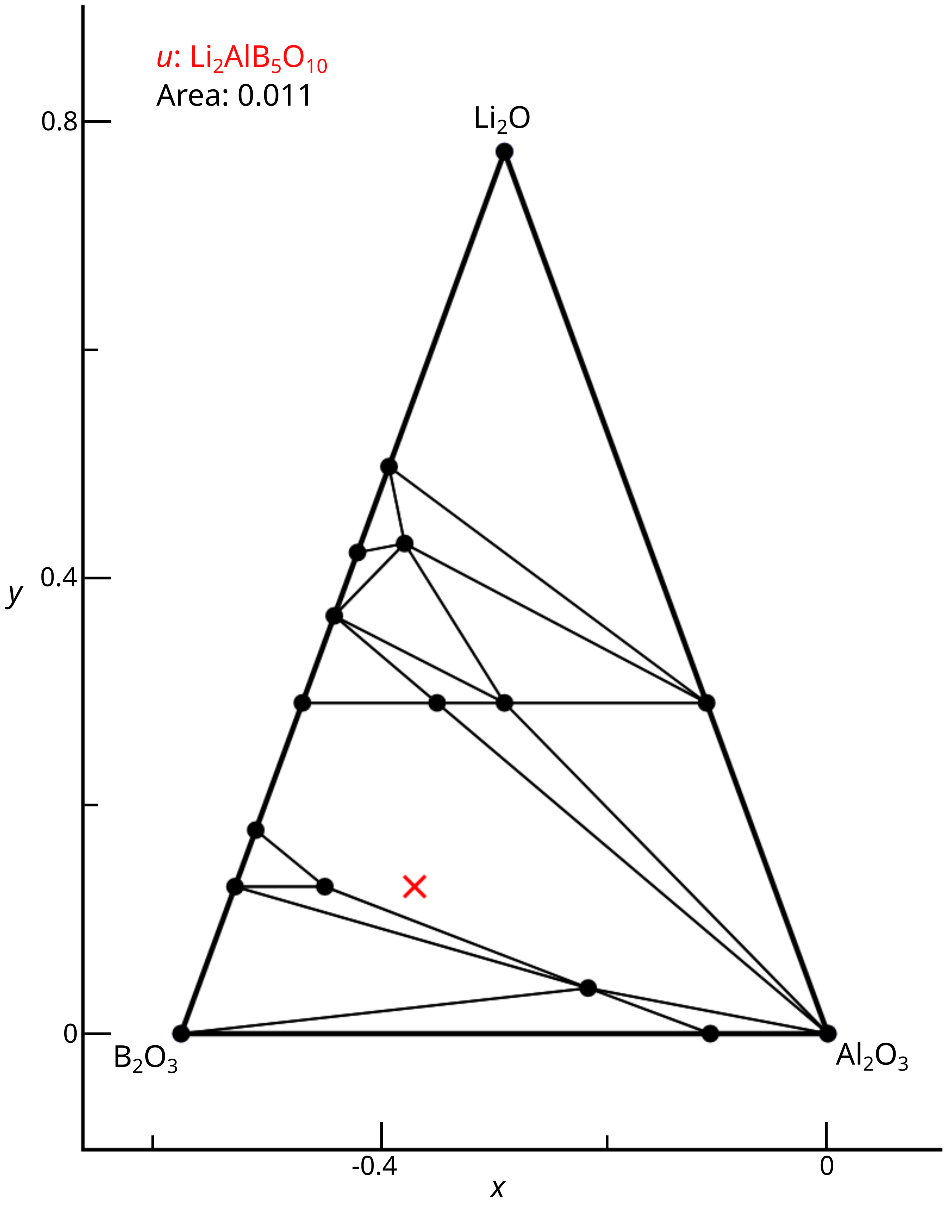

Figure S26: The Li^{1+}-Al^{3+}-B^{3+}-O^{2-} phase field, assuming the $Li_2AlB_5O_{10}$ phase is unknown. Area describes the relative area of the region which would form $Li_2AlB_5O_{10}$ at thermodynamic equilibrium.

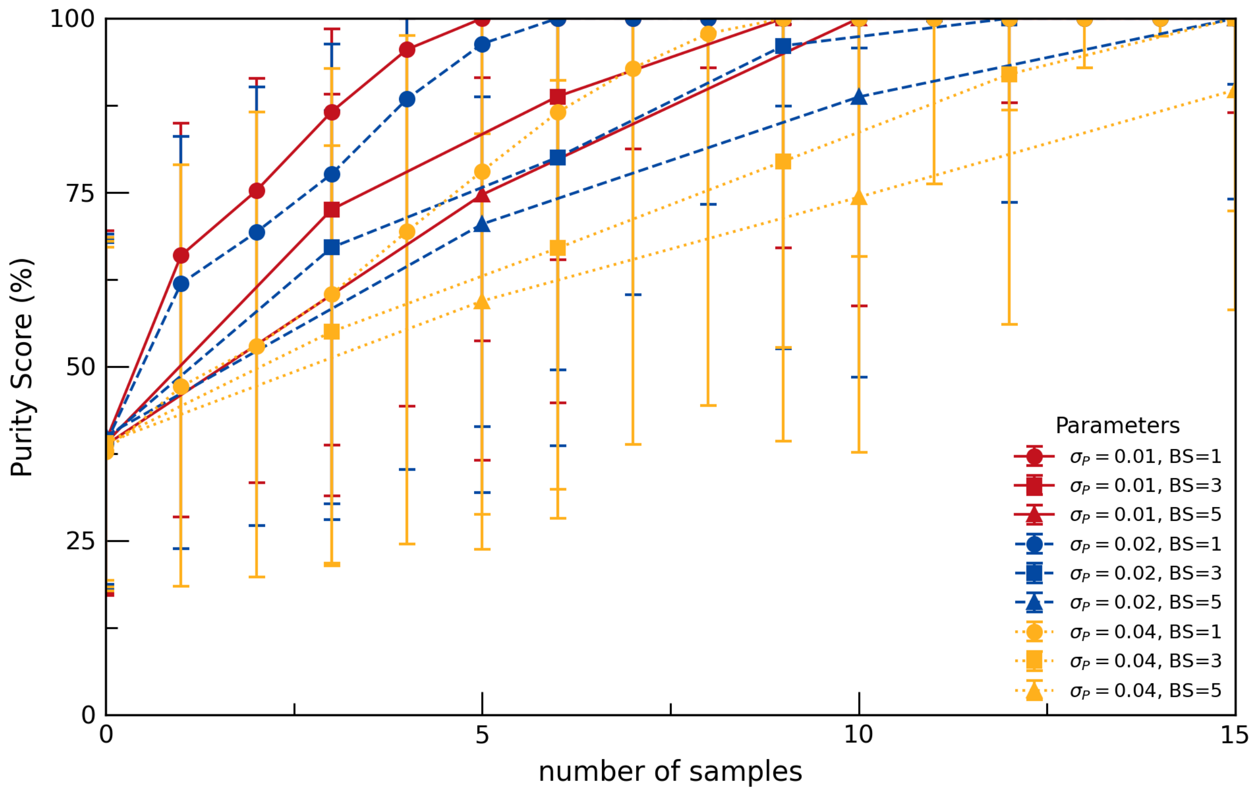

Figure S27: Median Purity Score vs number of samples for the $Li_2AlB_5O_{10}$ phase and an experimental error of 2 wt% (σ_E=0.02). Results are shown for all combinations of PICIP error (σ_P) and Batch size (BS). The error bars show the 16th and 84th percentiles respectively.

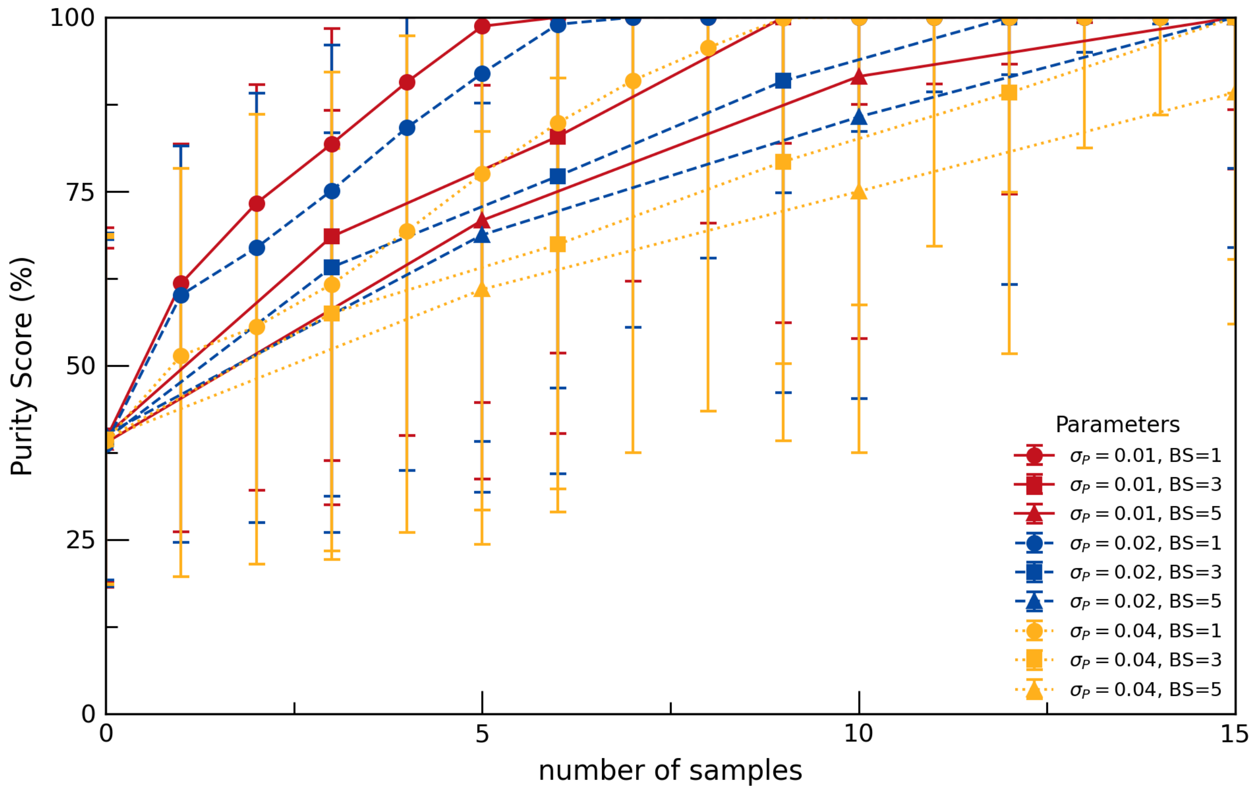

Figure S28: Median Purity Score vs number of samples for the $Li_2AlB_5O_{10}$ phase and an experimental error of 5 wt% (σ_E=0.05). Results are shown for all combinations of PICIP error (σ_P) and Batch size (BS). The error bars show the 16th and 84th percentiles respectively.

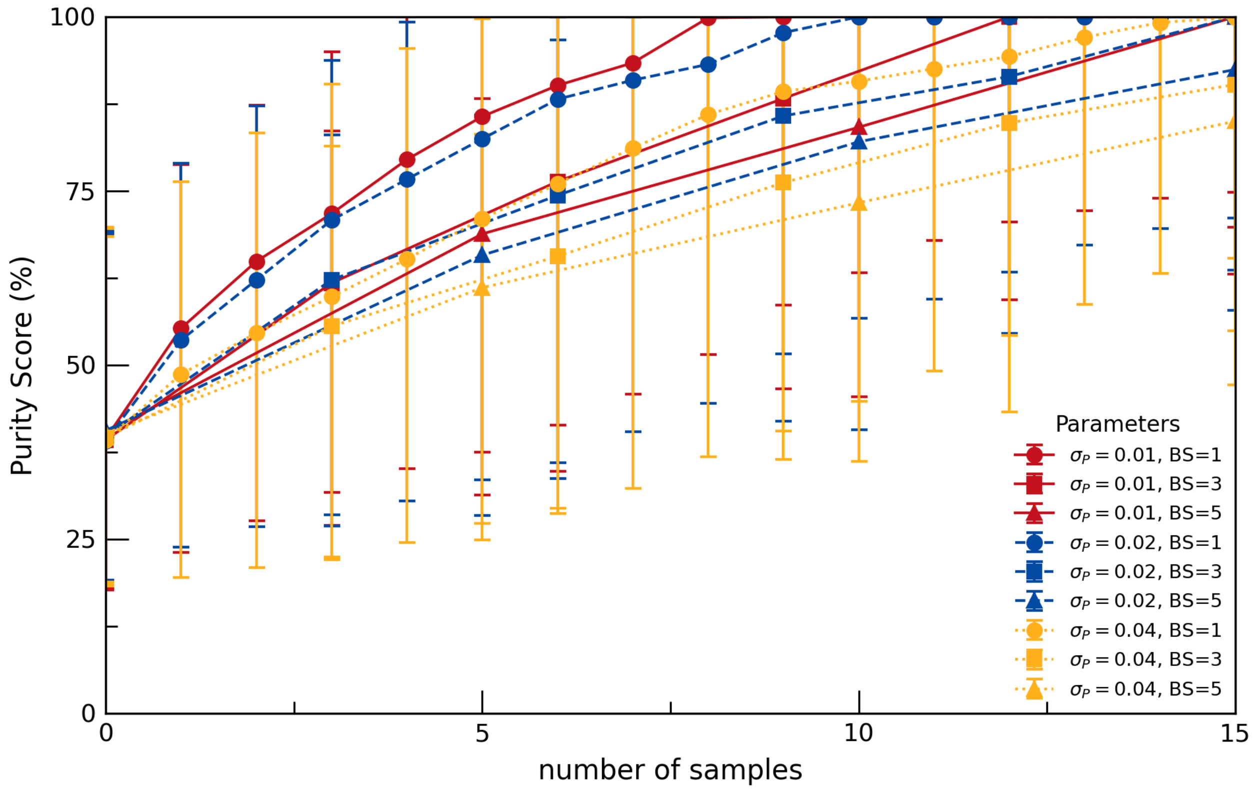

Figure S29: Median Purity Score vs number of samples for the $Li_2AlB_5O_{10}$ phase and an experimental error of 10 wt% (σ_E=0.1). Results are shown for all combinations of PICIP error (σ_P) and Batch size (BS). The error bars show the 16th and 84th percentiles respectively.

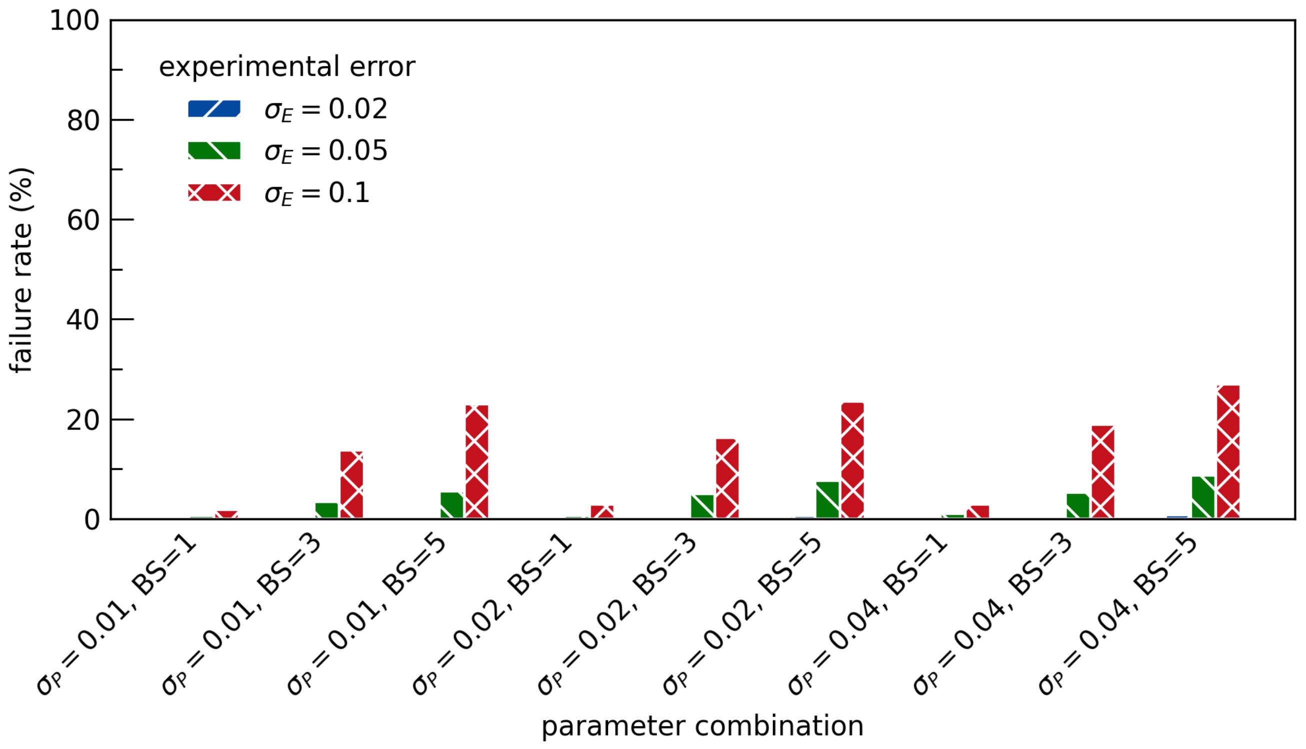

Figure S30: PICIP's failure rate for the $Li_2AlB_5O_{10}$ phase, for all combinations of PICIP error (σ_P), Experimental error (σ_E), and Batch size (BS)

2.4 Li_2AlBO_4

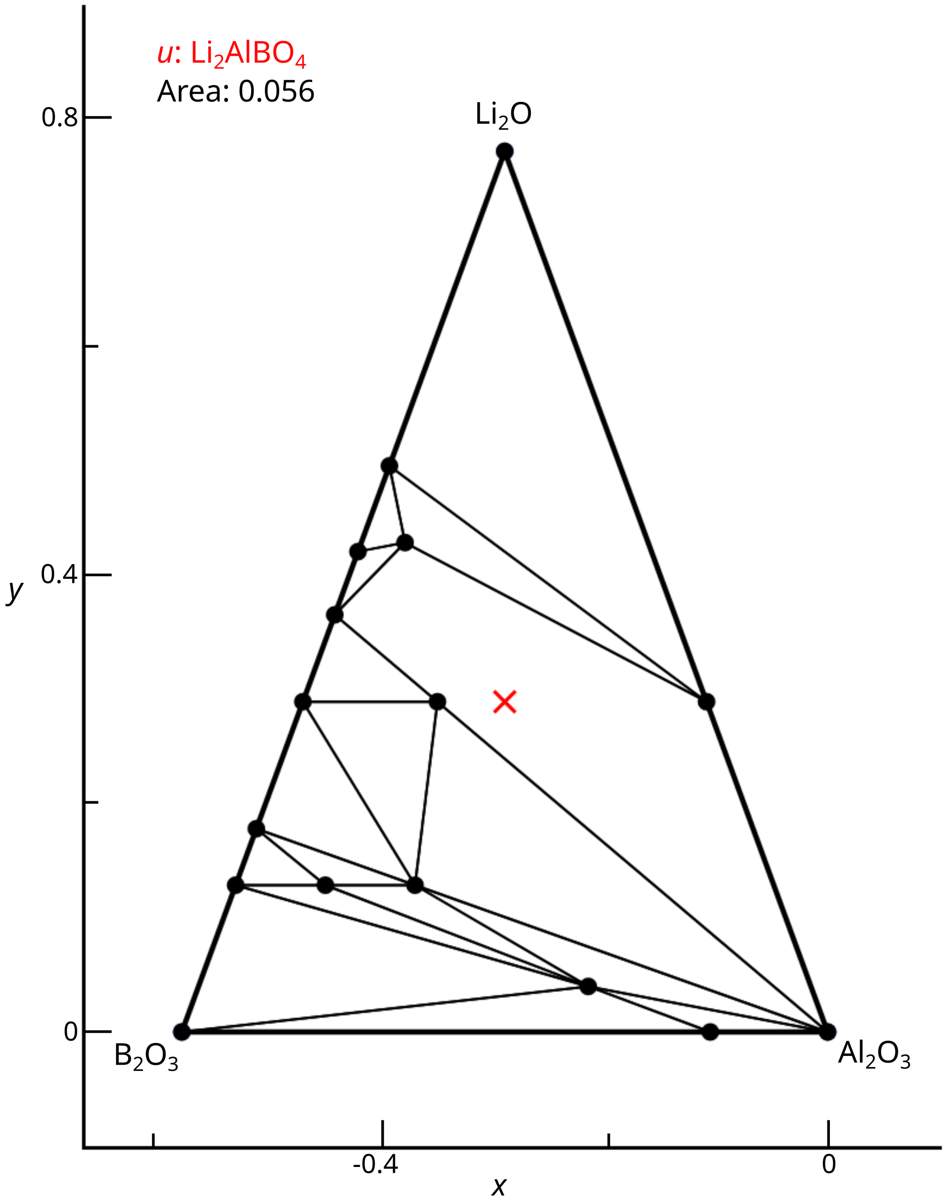

Figure S31: The Li^{1+}-Al^{3+}-B^{3+}-O^{2-} phase field, assuming the Li_2AlBO_4 phase is unknown. Area describes the relative area of the region which would form Li_2AlBO_4 at thermodynamic equilibrium.

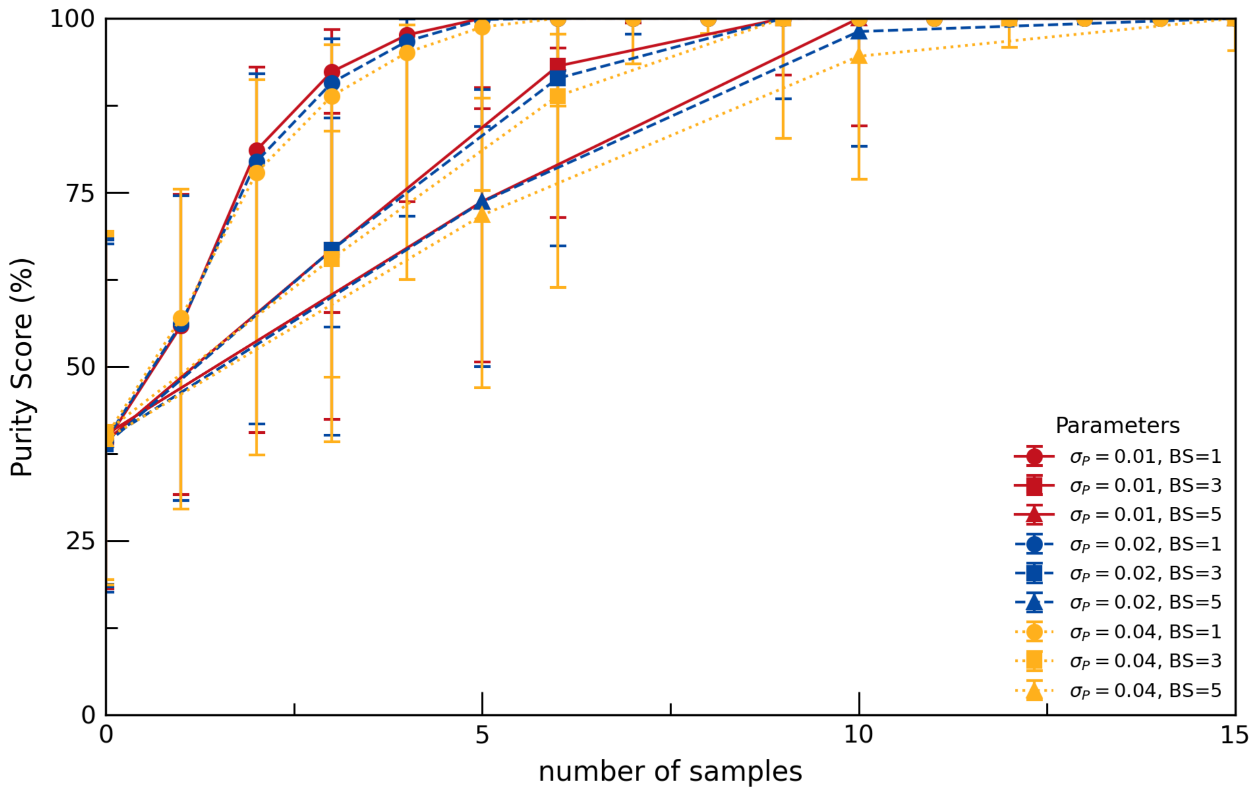

Figure S32: Median Purity Score vs number of samples for the Li_2AlBO_4 phase and an experimental error of 2 wt% (σ_E=0.02). Results are shown for all combinations of PICIP error (σ_P) and Batch size (BS). The error bars show the 16th and 84th percentiles respectively.

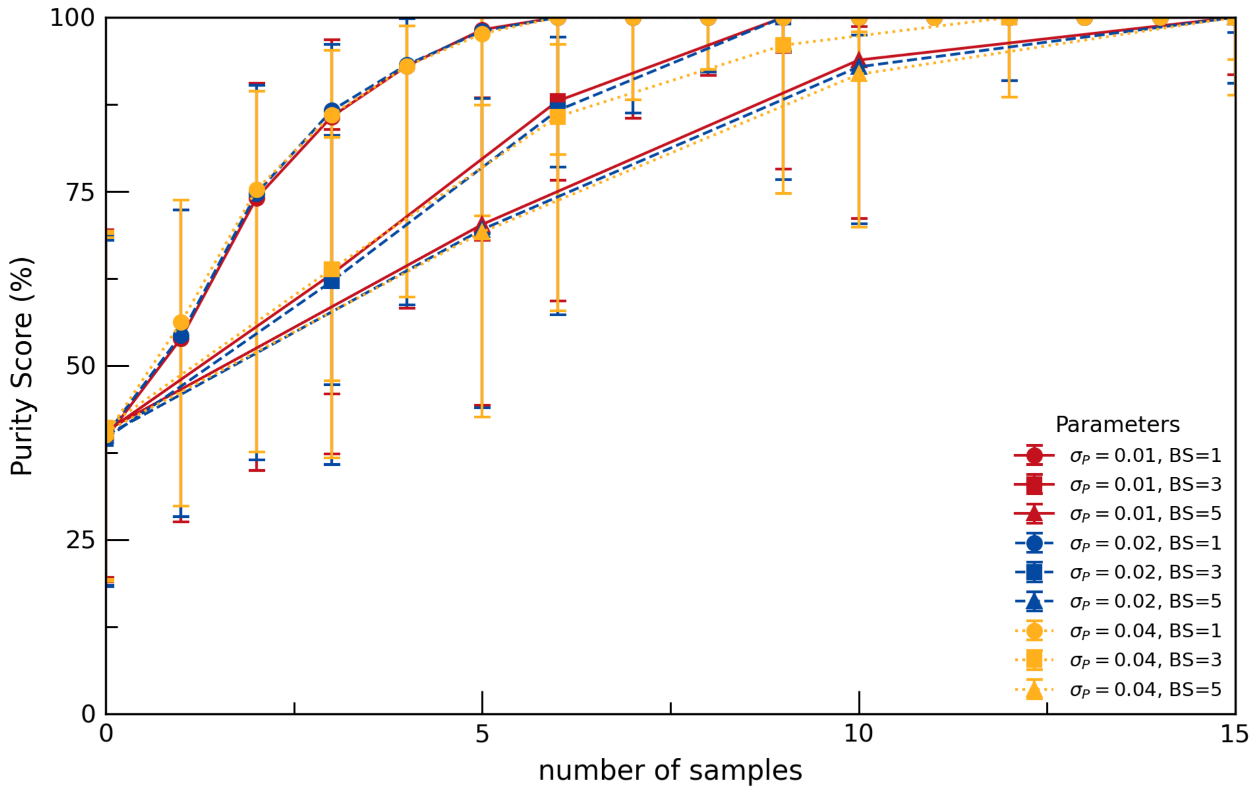

Figure S33: Median Purity Score vs number of samples for the Li_2AlBO_4 phase and an experimental error of 5 wt% (σ_E=0.05). Results are shown for all combinations of PICIP error (σ_P) and Batch size (BS). The error bars show the 16th and 84th percentiles respectively.

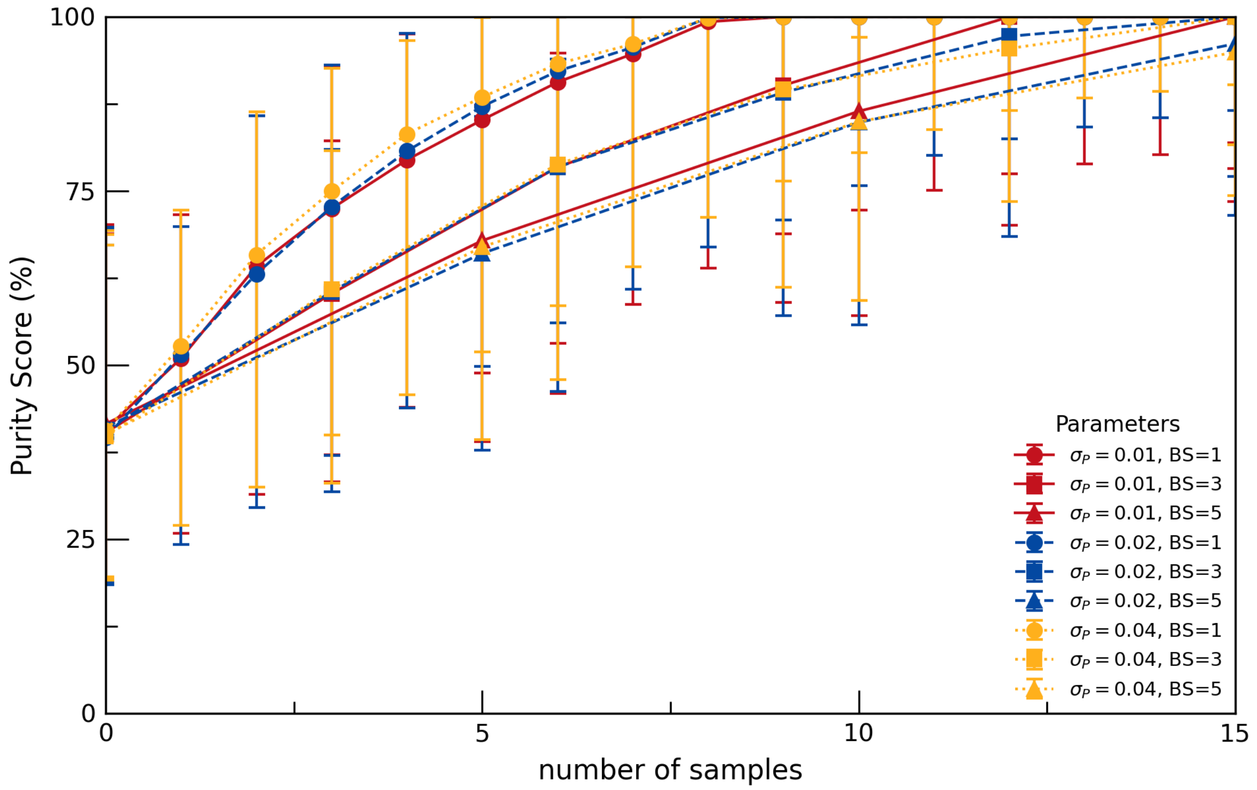

Figure S34: Median Purity Score vs number of samples for the Li_2AlBO_4 phase and an experimental error of 10 wt% (σ_E=0.1). Results are shown for all combinations of PICIP error (σ_P) and Batch size (BS). The error bars show the 16th and 84th percentiles respectively.

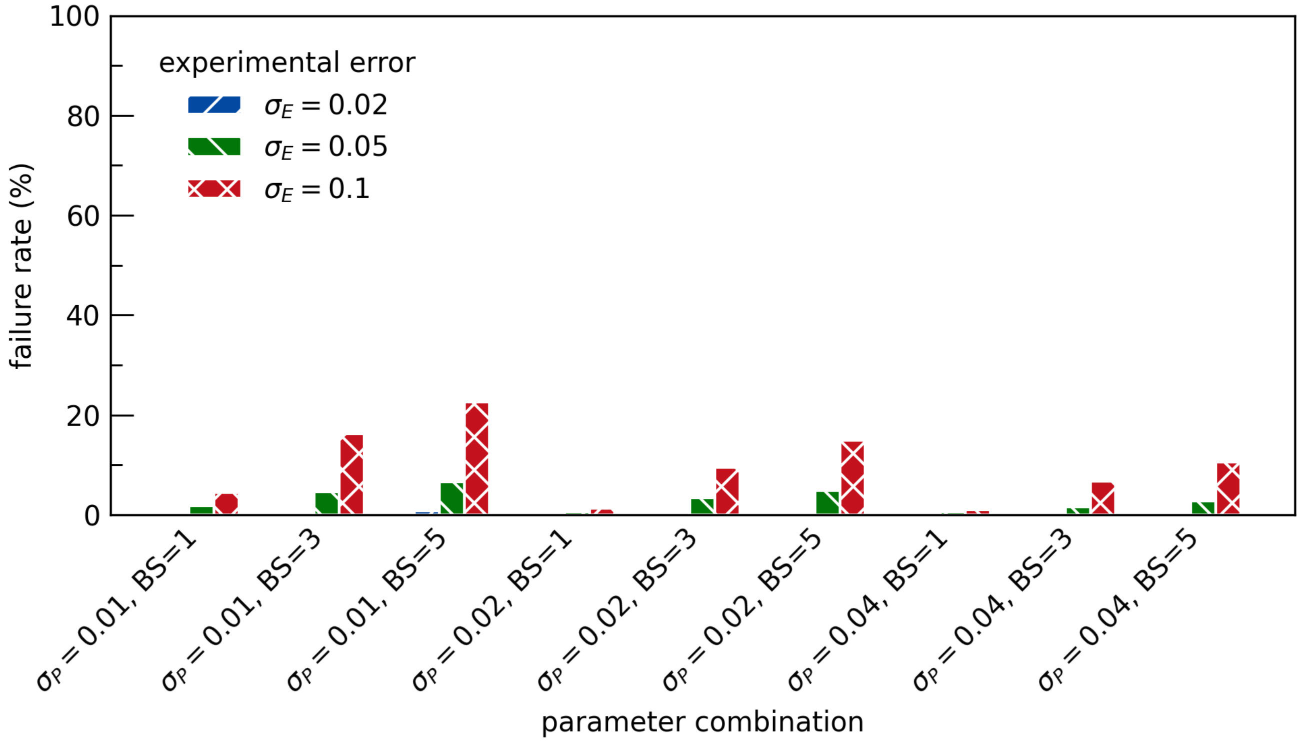

Figure S35: PICIP's failure rate for the Li_2AlBO_4 phase, for all combinations of PICIP error (σ_P), Experimental error (σ_E), and Batch size (BS)

2.5 $Li_3AlB_2O_6$

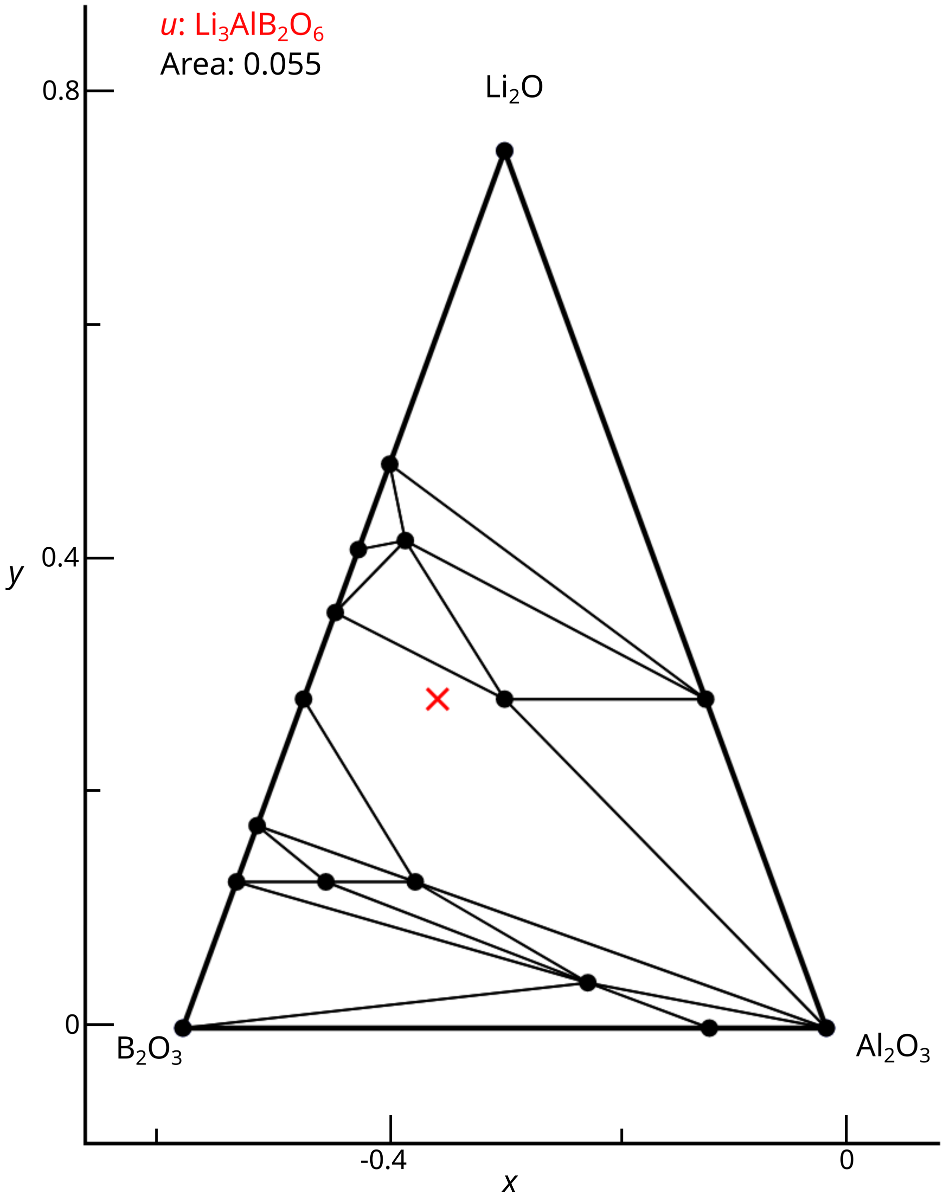

Figure S36: The Li^{1+}-Al^{3+}-B^{3+}-O^{2-} phase field, assuming the $Li_3AlB_2O_6$ phase is unknown. Area describes the relative area of the region which would form $Li_3AlB_2O_6$ at thermodynamic equilibrium.

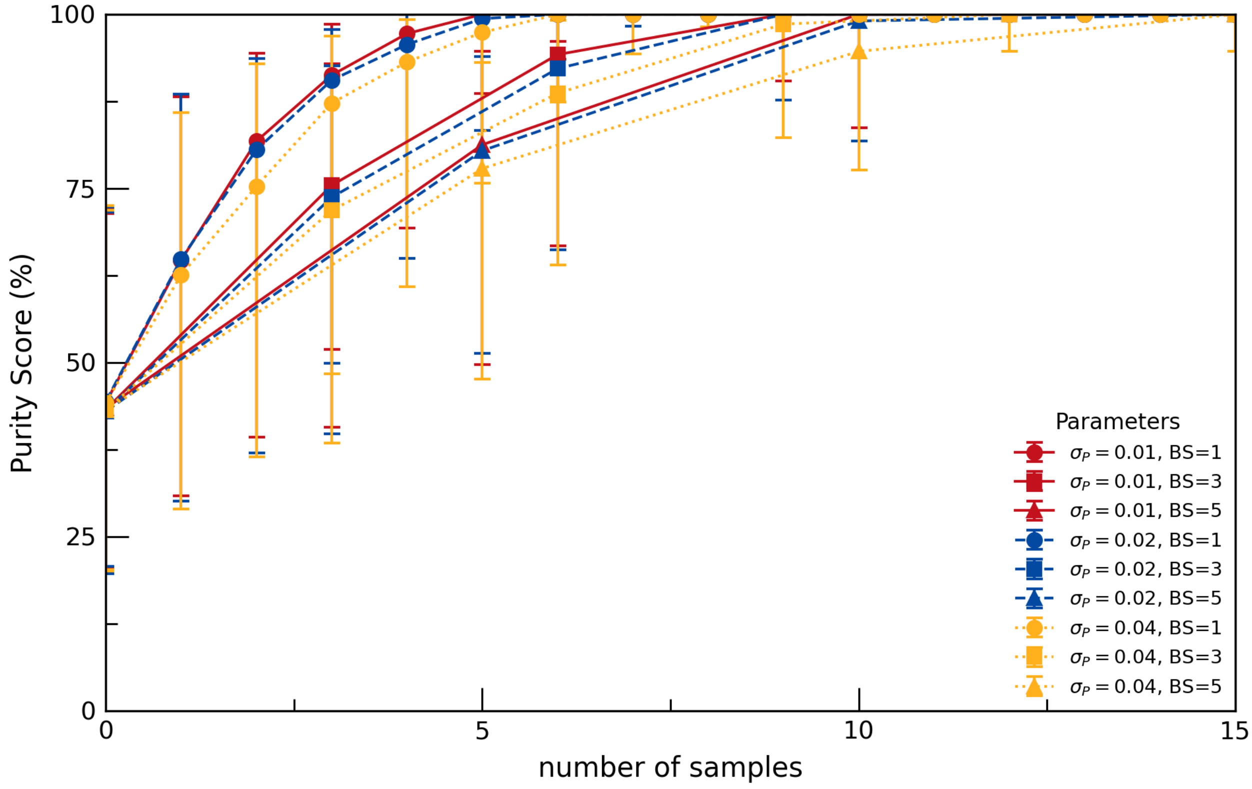

Figure S37: Median Purity Score vs number of samples for the $Li_3AlB_2O_6$ phase and an experimental error of 2 wt% (σ_E=0.02). Results are shown for all combinations of PICIP error (σ_P) and Batch size (BS). The error bars show the 16th and 84th percentiles respectively.

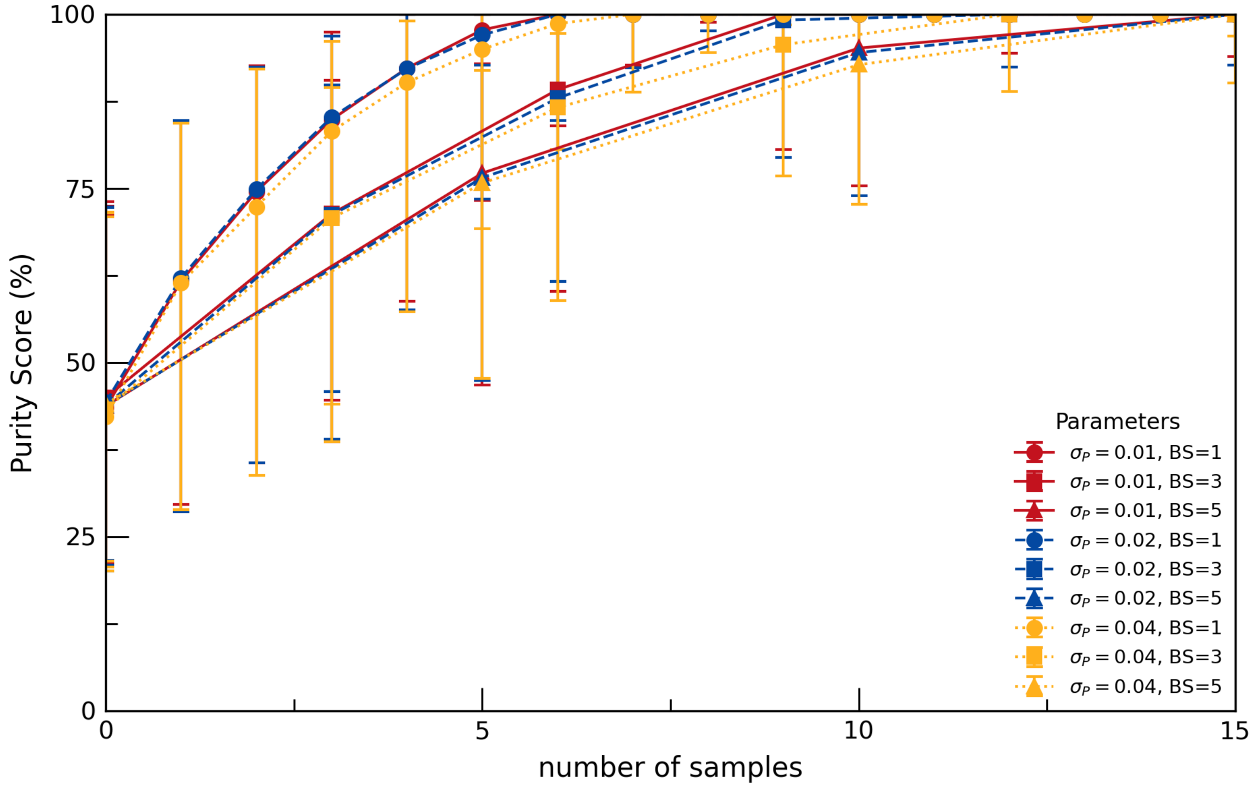

Figure S38: Median Purity Score vs number of samples for the $Li_3AlB_2O_6$ phase and an experimental error of 5 wt% (σ_E=0.05). Results are shown for all combinations of PICIP error (σ_P) and Batch size (BS). The error bars show the 16th and 84th percentiles respectively.

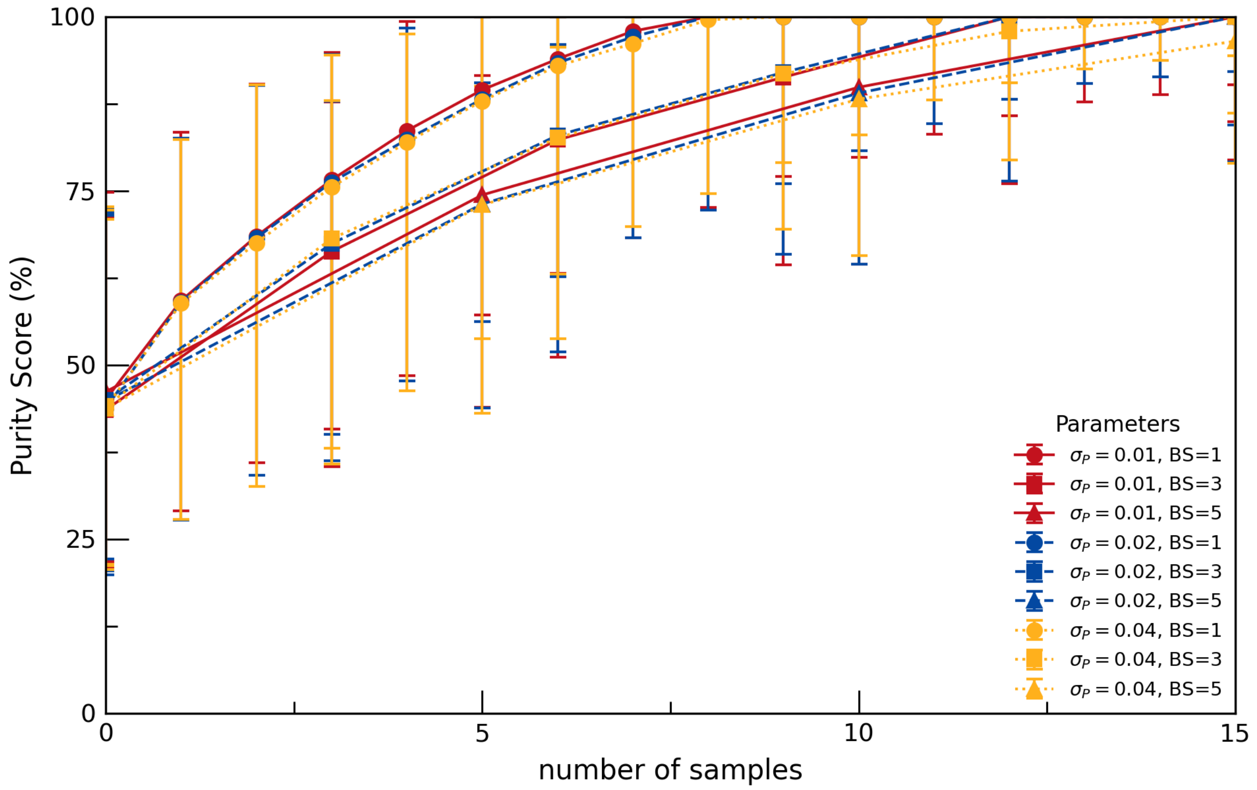

Figure S39: Median Purity Score vs number of samples for the $Li_3AlB_2O_6$ phase and an experimental error of 10 wt% (σ_E=0.1). Results are shown for all combinations of PICIP error (σ_P) and Batch size (BS). The error bars show the 16th and 84th percentiles respectively.

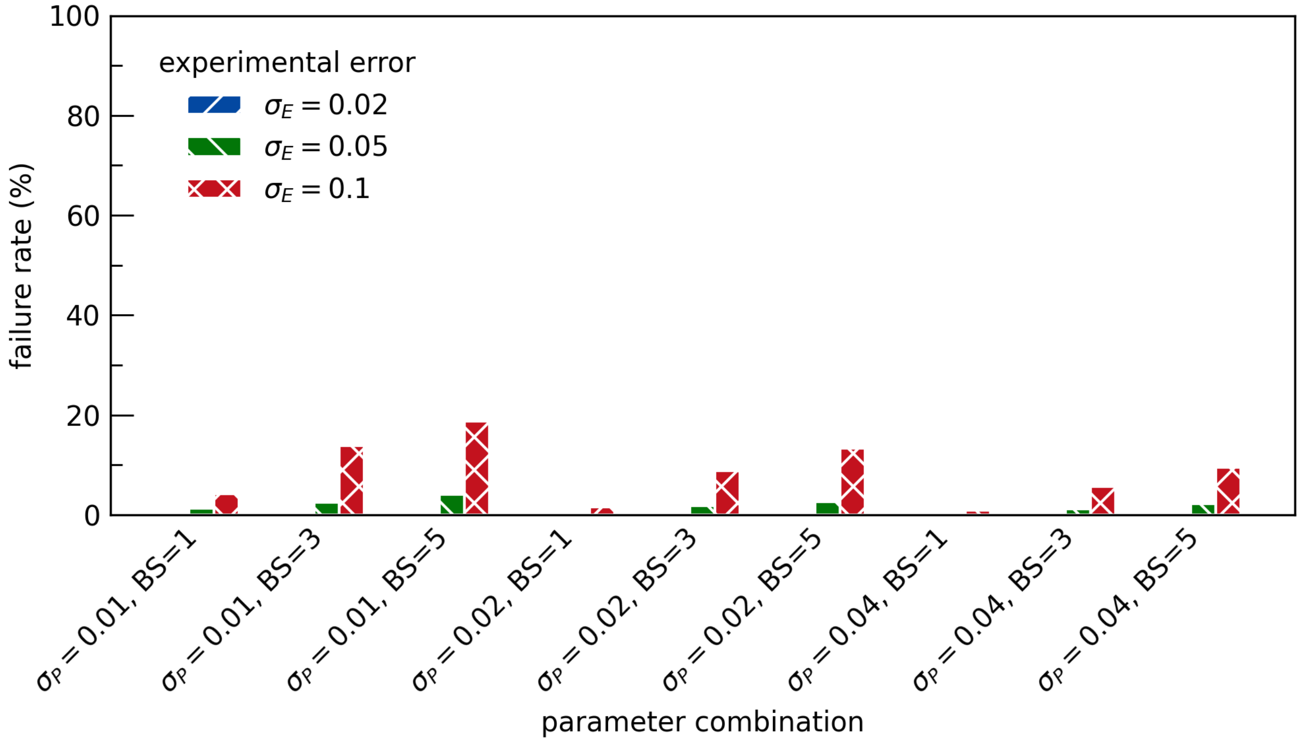

Figure S40: PICIP's failure rate for the $Li_3AlB_2O_6$ phase, for all combinations of PICIP error (σ_P), Experimental error (σ_E), and Batch size (BS)

2.6 $LiAlB_2O_5$

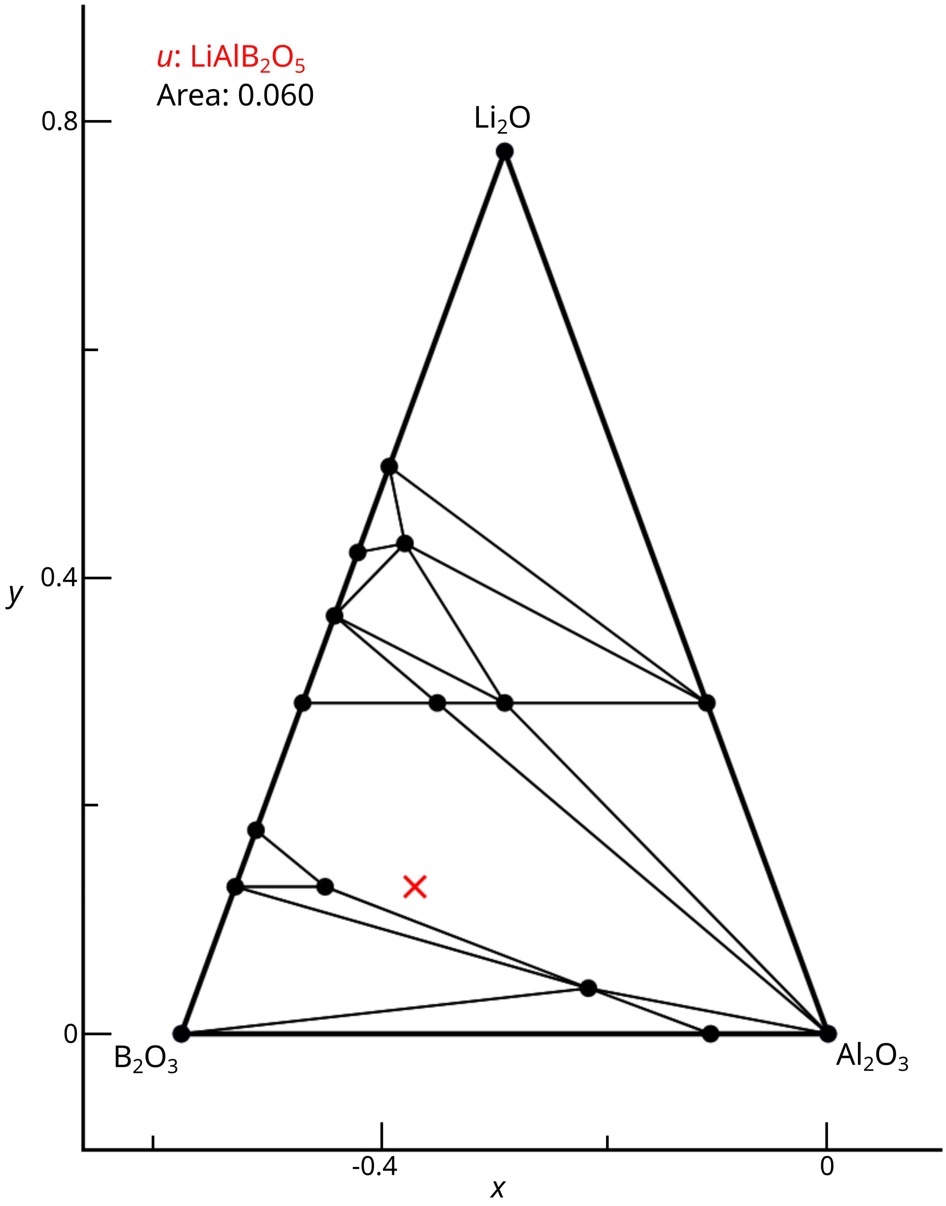

Figure S41: The Li^{1+}-Al^{3+}-B^{3+}-O^{2-} phase field, assuming the $LiAlB_2O_5$ phase is unknown. Area describes the relative area of the region which would form $LiAlB_2O_5$ at thermodynamic equilibrium.

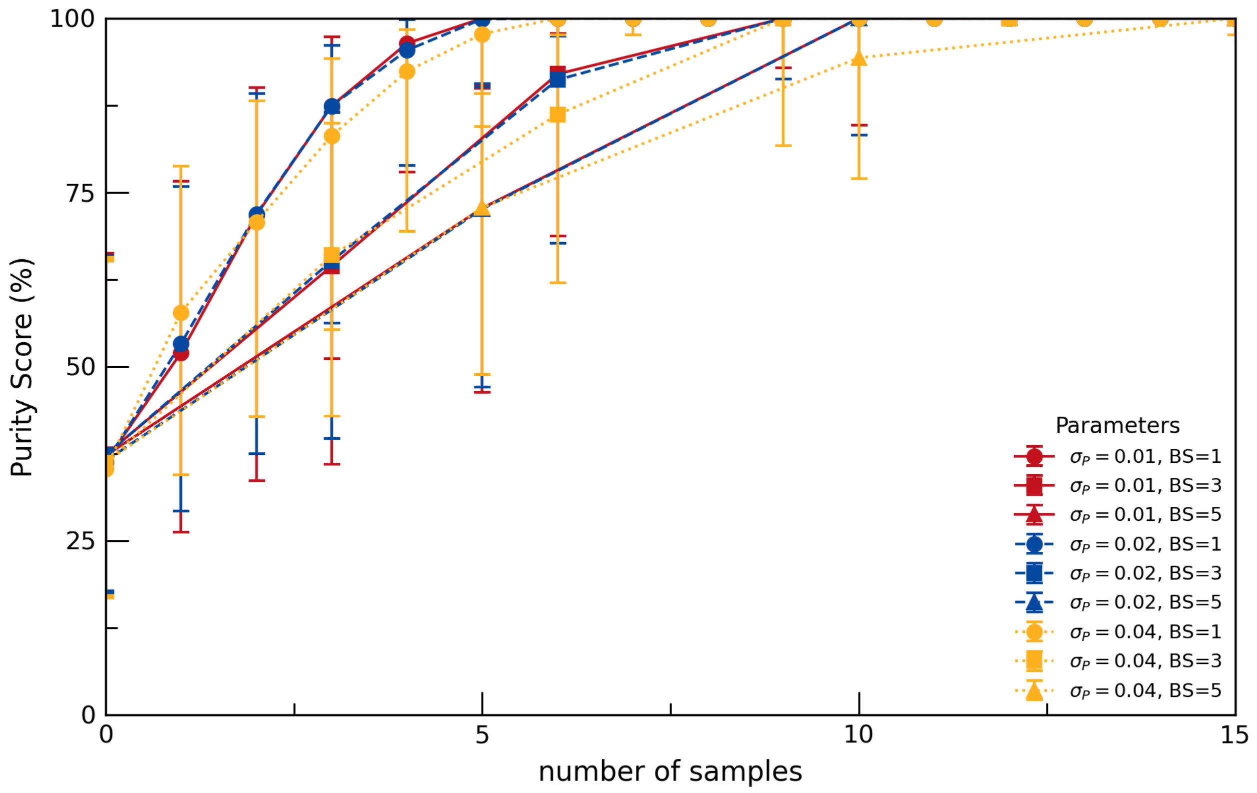

Figure S42: Median Purity Score vs number of samples for the $LiAlB_2O_5$ phase and an experimental error of 2 wt% (σ_E=0.02). Results are shown for all combinations of PICIP error (σ_P) and Batch size (BS). The error bars show the 16th and 84th percentiles respectively.

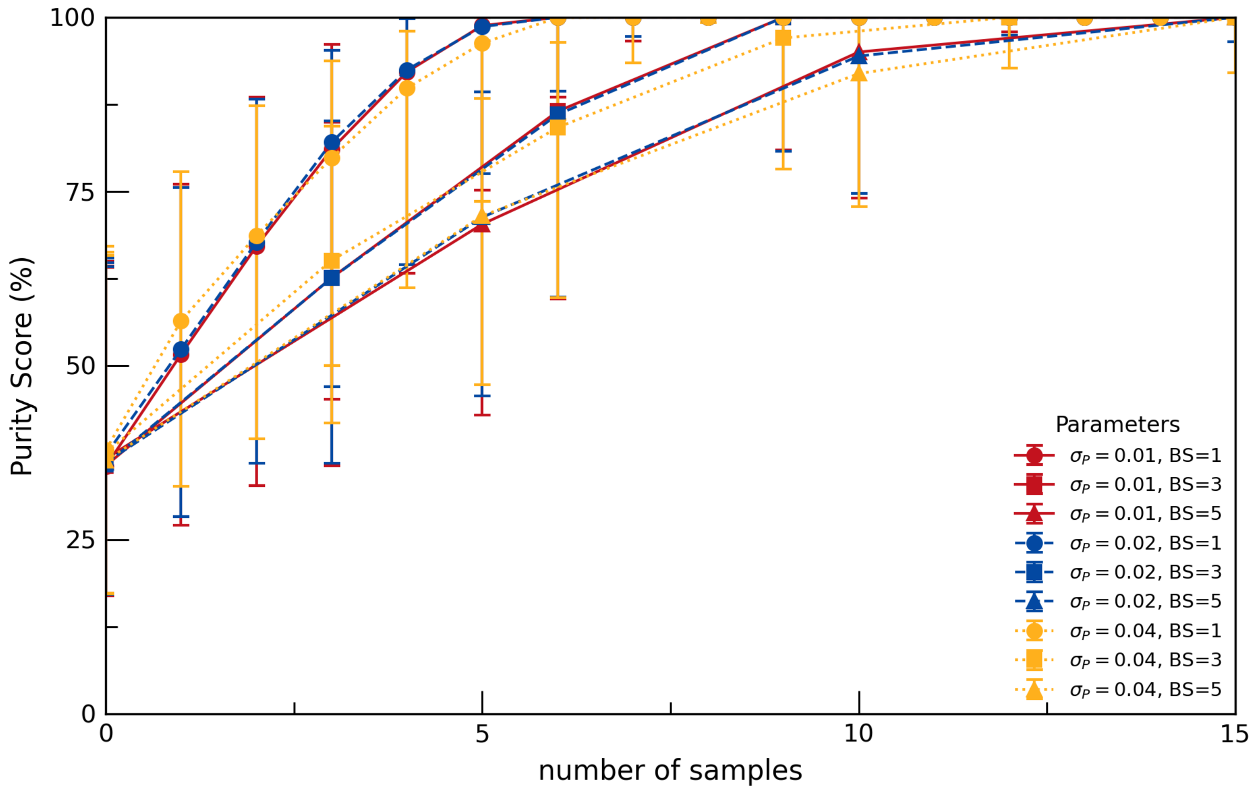

Figure S43: Median Purity Score vs number of samples for the $LiAlB_2O_5$ phase and an experimental error of 5 wt% (σ_E=0.05). Results are shown for all combinations of PICIP error (σ_P) and Batch size (BS). The error bars show the 16th and 84th percentiles respectively.

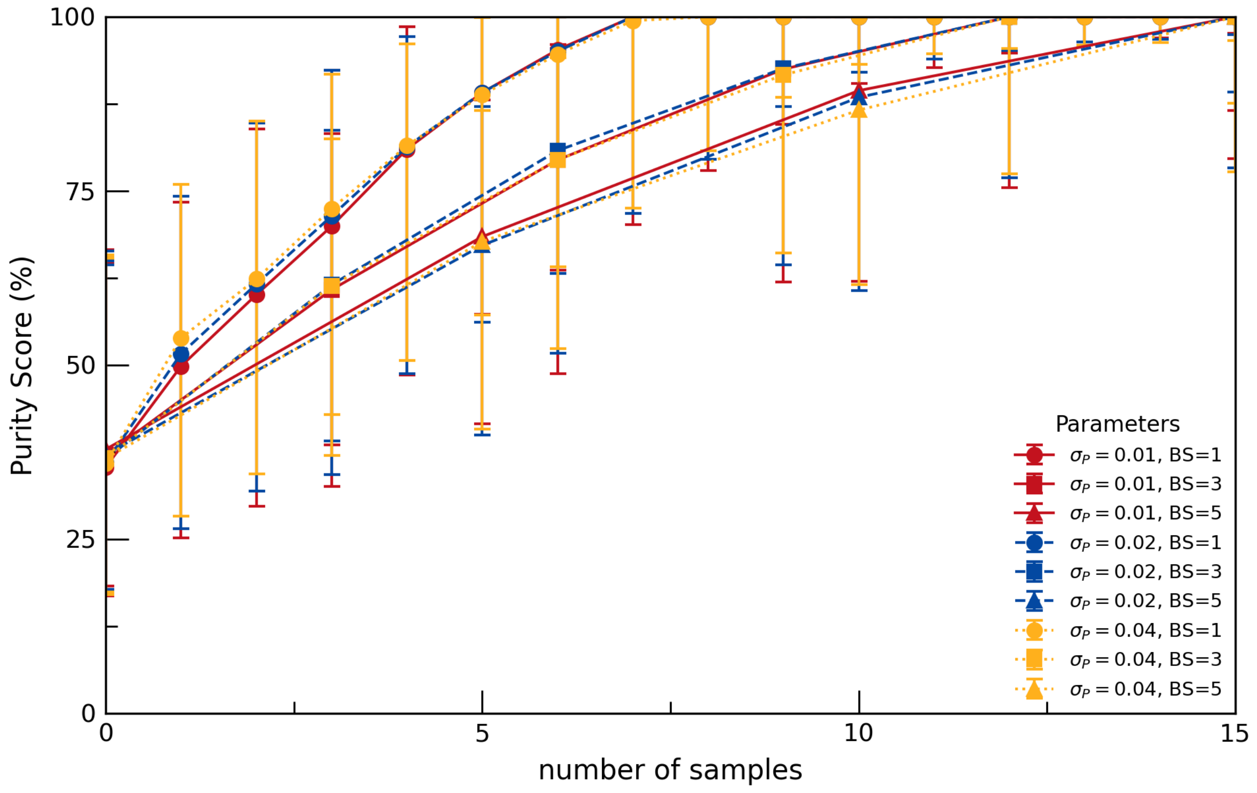

Figure S44: Median Purity Score vs number of samples for the $LiAlB_2O_5$ phase and an experimental error of 10 wt% (σ_E=0.1). Results are shown for all combinations of PICIP error (σ_P) and Batch size (BS). The error bars show the 16th and 84th percentiles respectively.

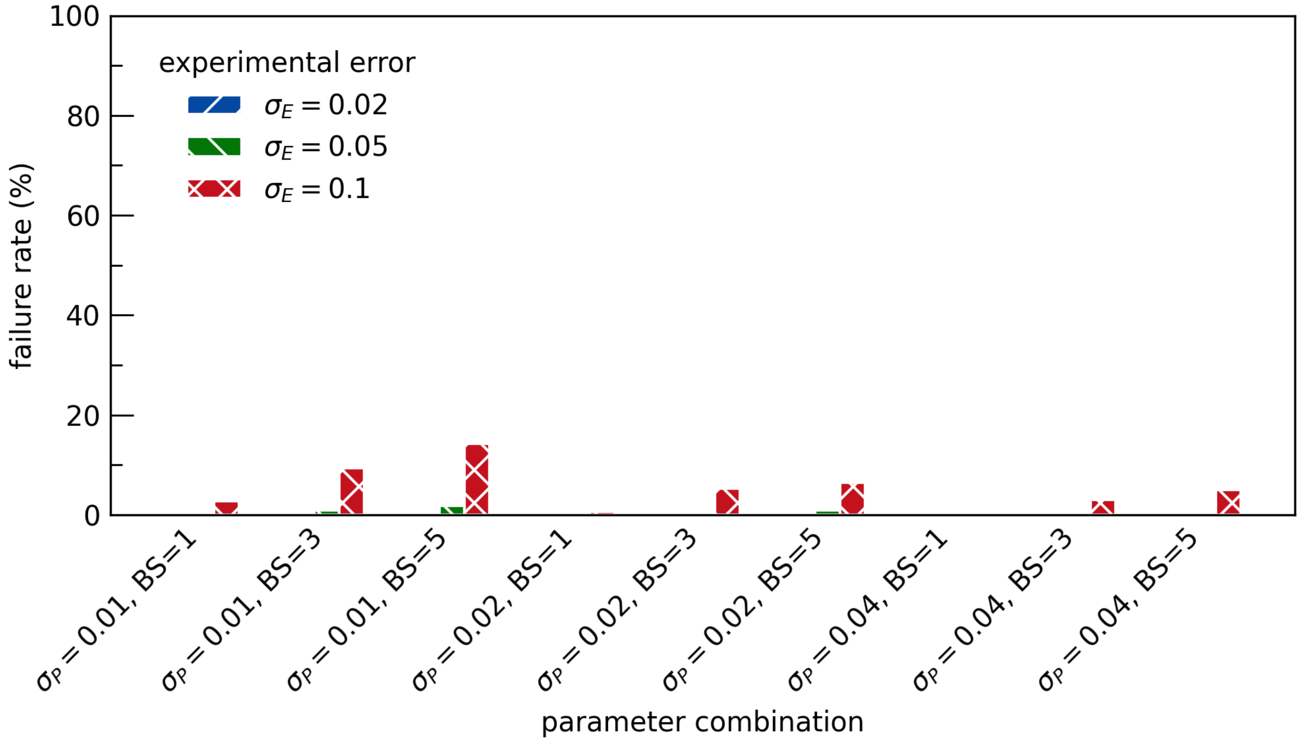

Figure S45: PICIP's failure rate for the $LiAlB_2O_5$ phase, for all combinations of PICIP error (σ_P), Experimental error (σ_E), and Batch size (BS)

3 Mg^{2+}-B^{3+}-O^{2-}-F^{1-}

3.1 $Mg_3B(OF)_3$

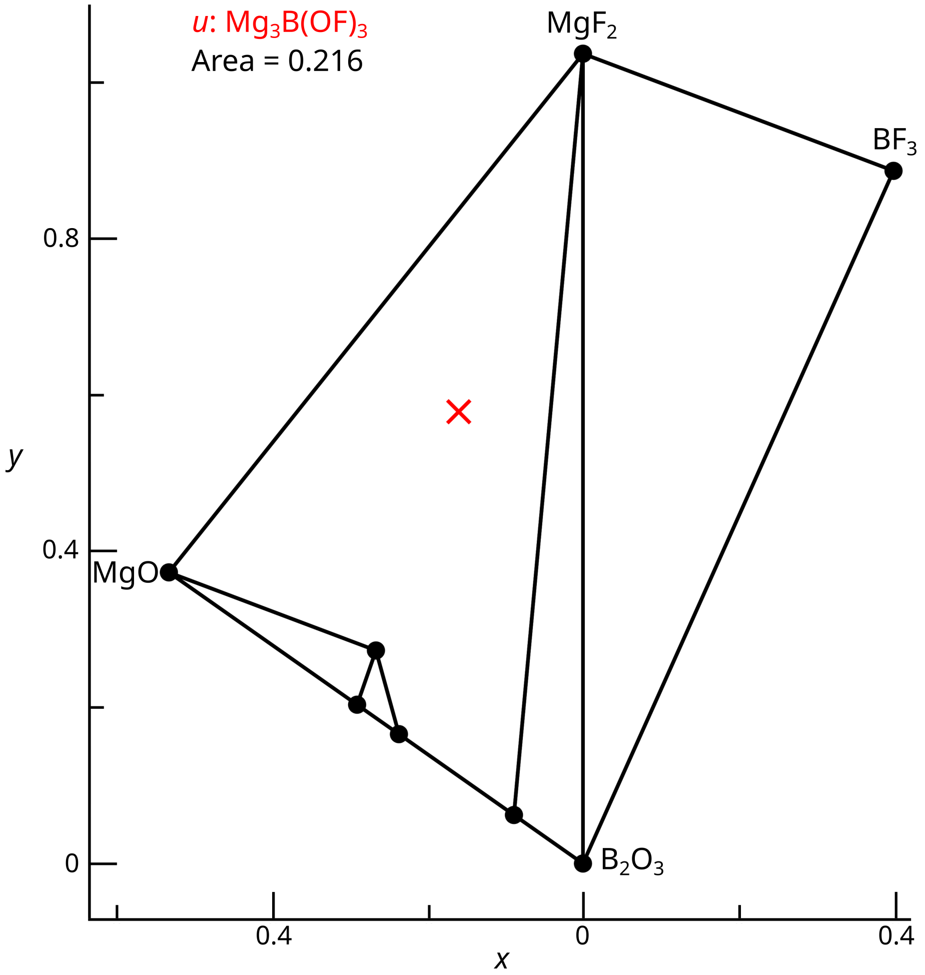

Figure S46: The Li^{1+}-Al^{3+}-B^{3+}-O^{2-} phase field, assuming the $Mg_3B(OF)_3$ phase is unknown. Area describes the relative area of the region which would form $Mg_3B(OF)_3$ at thermodynamic equilibrium.

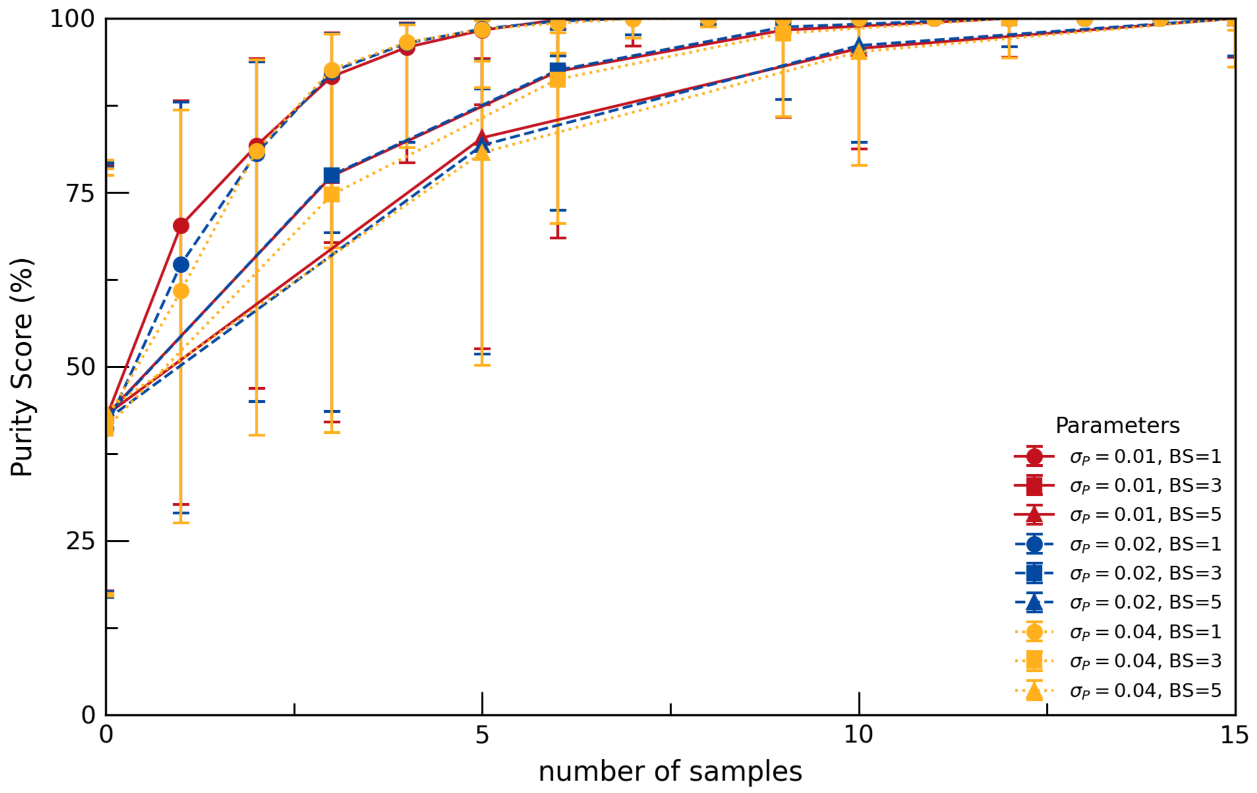

Figure S47: Median Purity Score vs number of samples for the $Mg_3B(OF)_3$ phase and an experimental error of 2 wt% (σ_E=0.02). Results are shown for all combinations of PICIP error (σ_P) and Batch size (BS). The error bars show the 16th and 84th percentiles respectively.

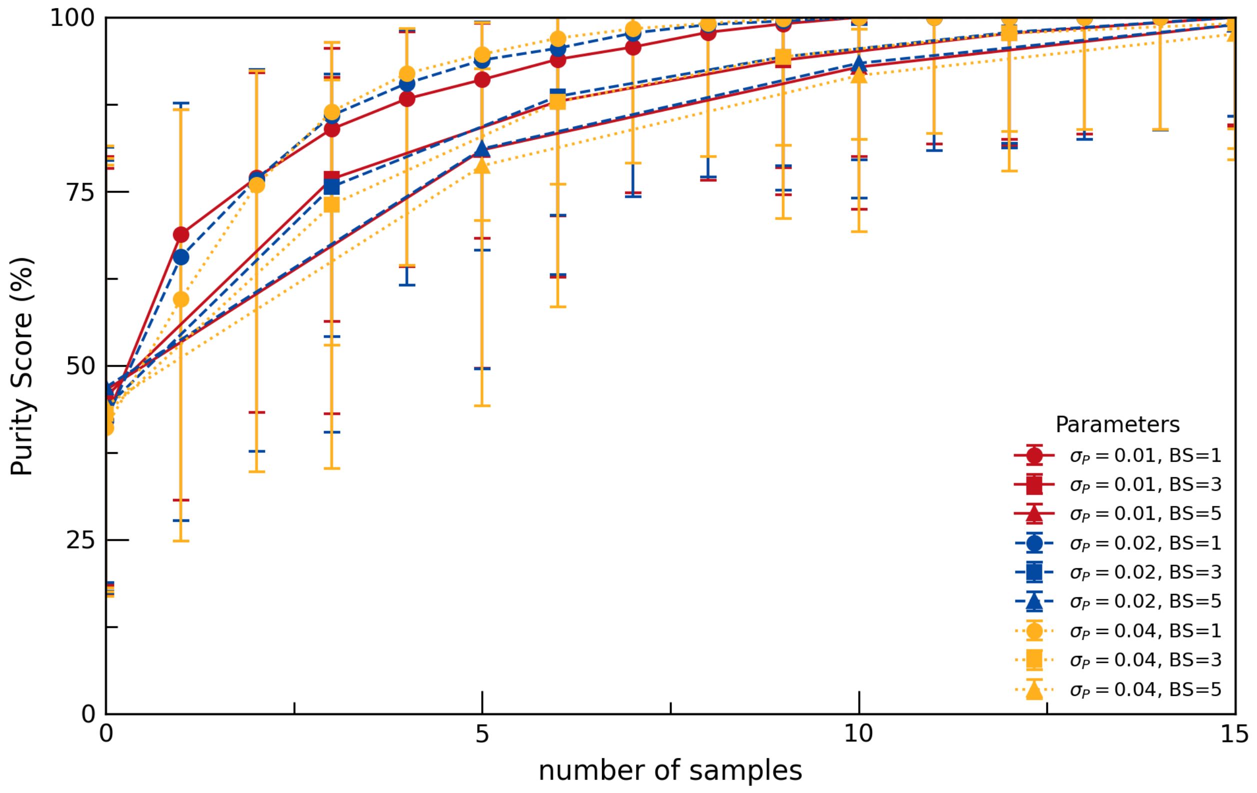

Figure S48: Median Purity Score vs number of samples for the $Mg_3B(OF)_3$ phase and an experimental error of 5 wt% (σ_E=0.05). Results are shown for all combinations of PICIP error (σ_P) and Batch size (BS). The error bars show the 16th and 84th percentiles respectively.

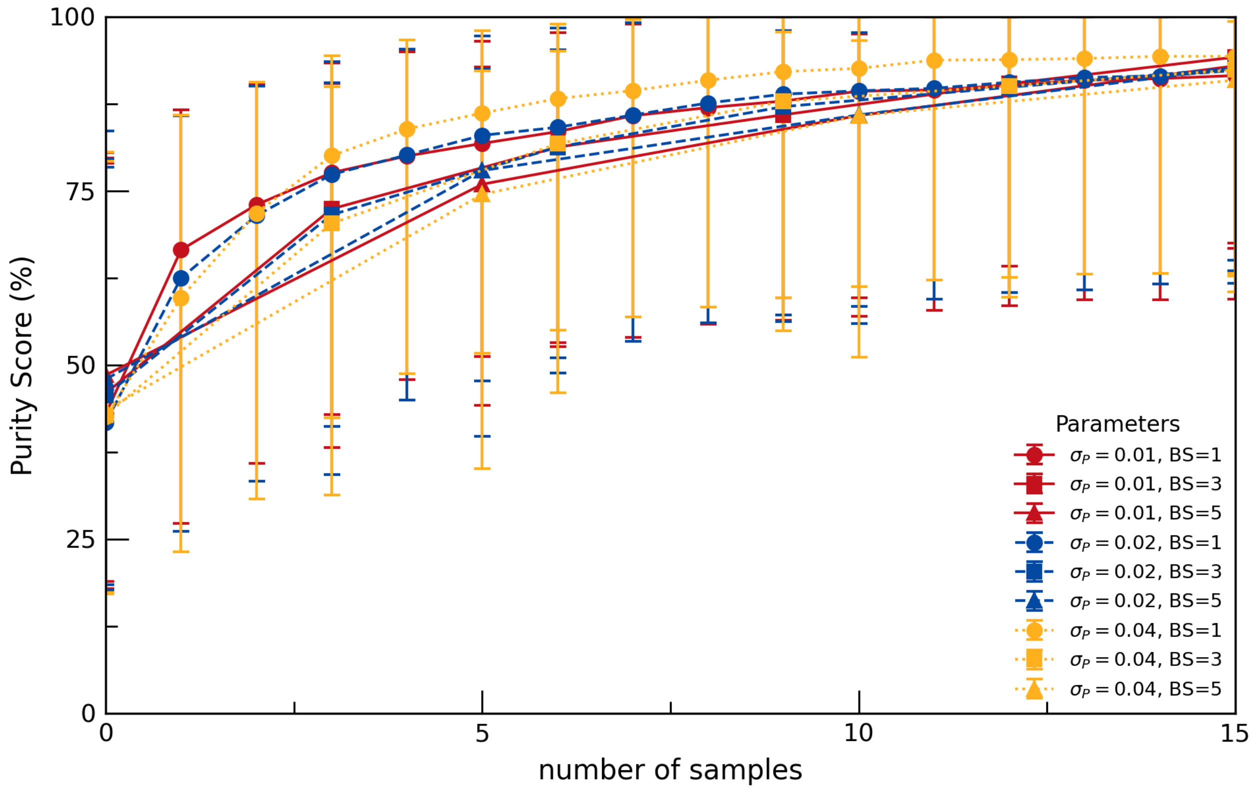

Figure S49: Median Purity Score vs number of samples for the $Mg_3B(OF)_3$ phase and an experimental error of 10 wt% (σ_E=0.1). Results are shown for all combinations of PICIP error (σ_P) and Batch size (BS). The error bars show the 16th and 84th percentiles respectively.

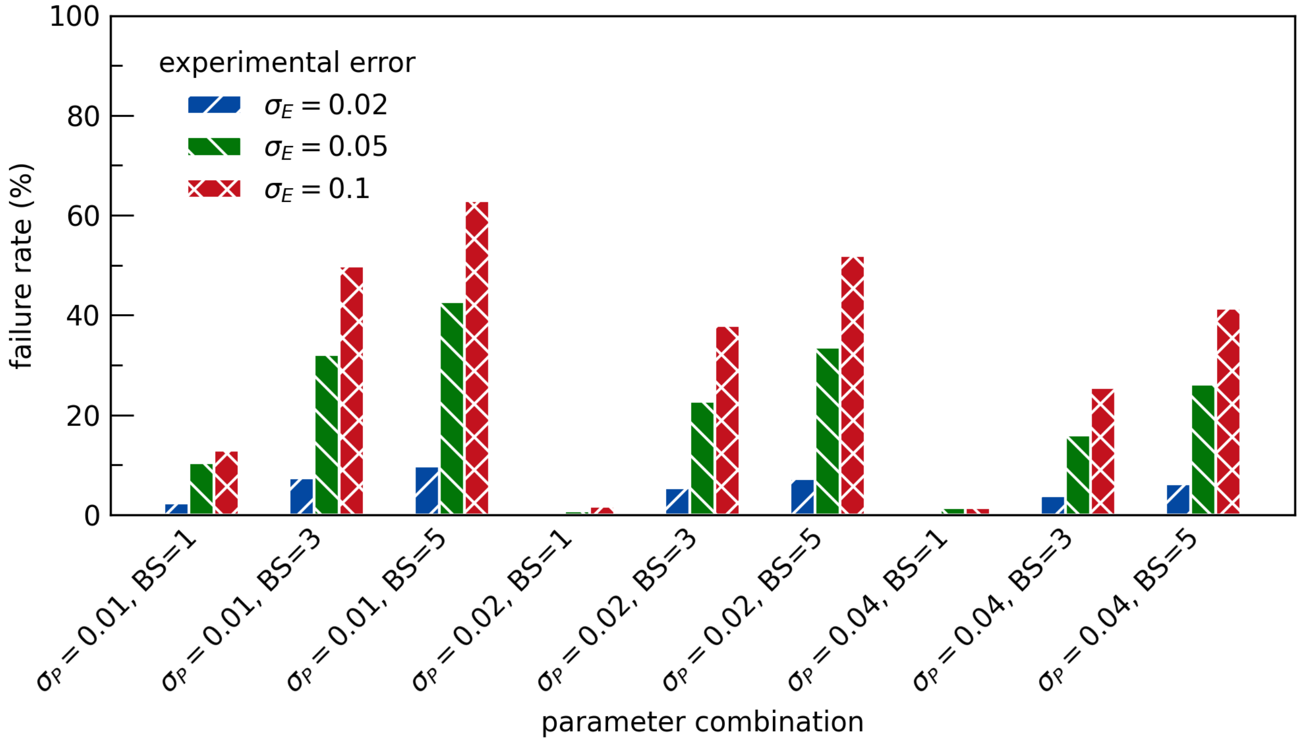

Figure S50: PICIP's failure rate for the $Mg_3B(OF)_3$ phase, for all combinations of PICIP error (σ_P), Experimental error (σ_E), and Batch size (BS)

3.2 $Mg_5B_3O_9F$

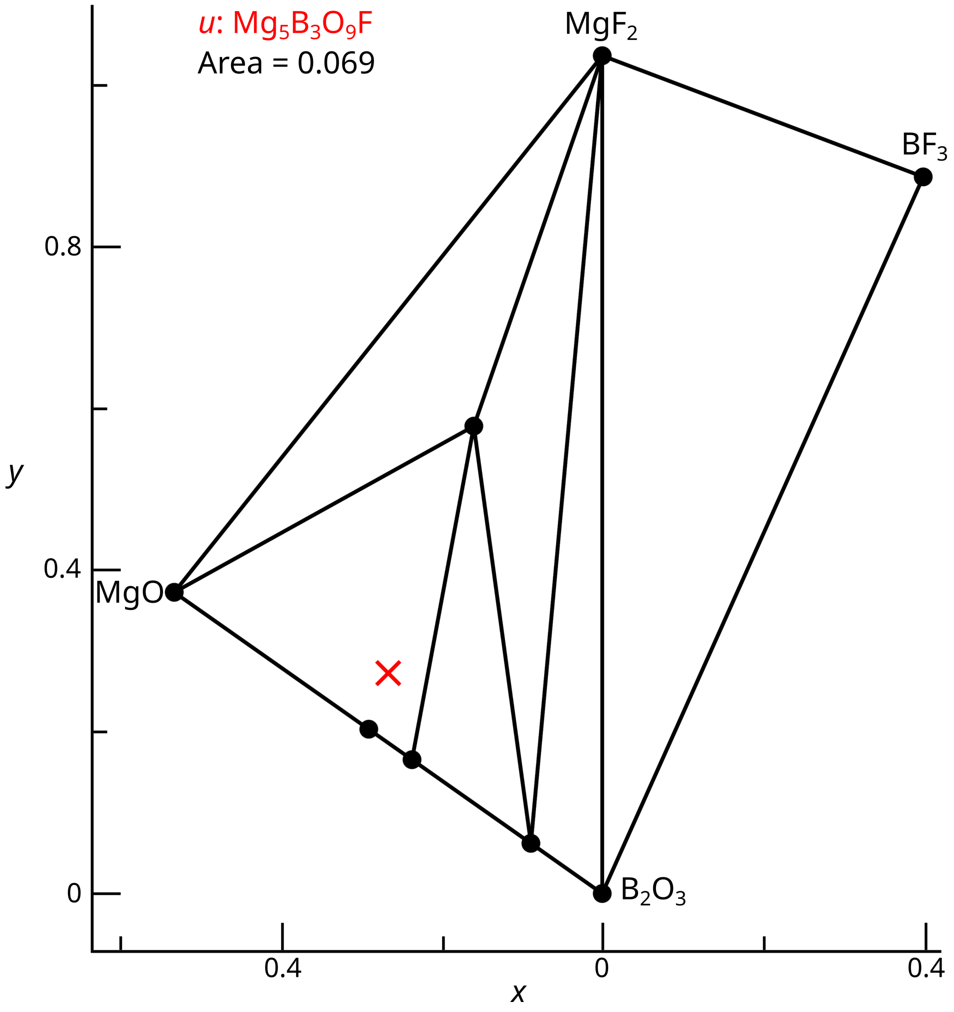

Figure S51: The Li^{1+}-Al^{3+}-B^{3+}-O^{2-} phase field, assuming the $Mg_5B_3O_9F$ phase is unknown. Area describes the relative area of the region which would form $Mg_5B_3O_9F$ at thermodynamic equilibrium.

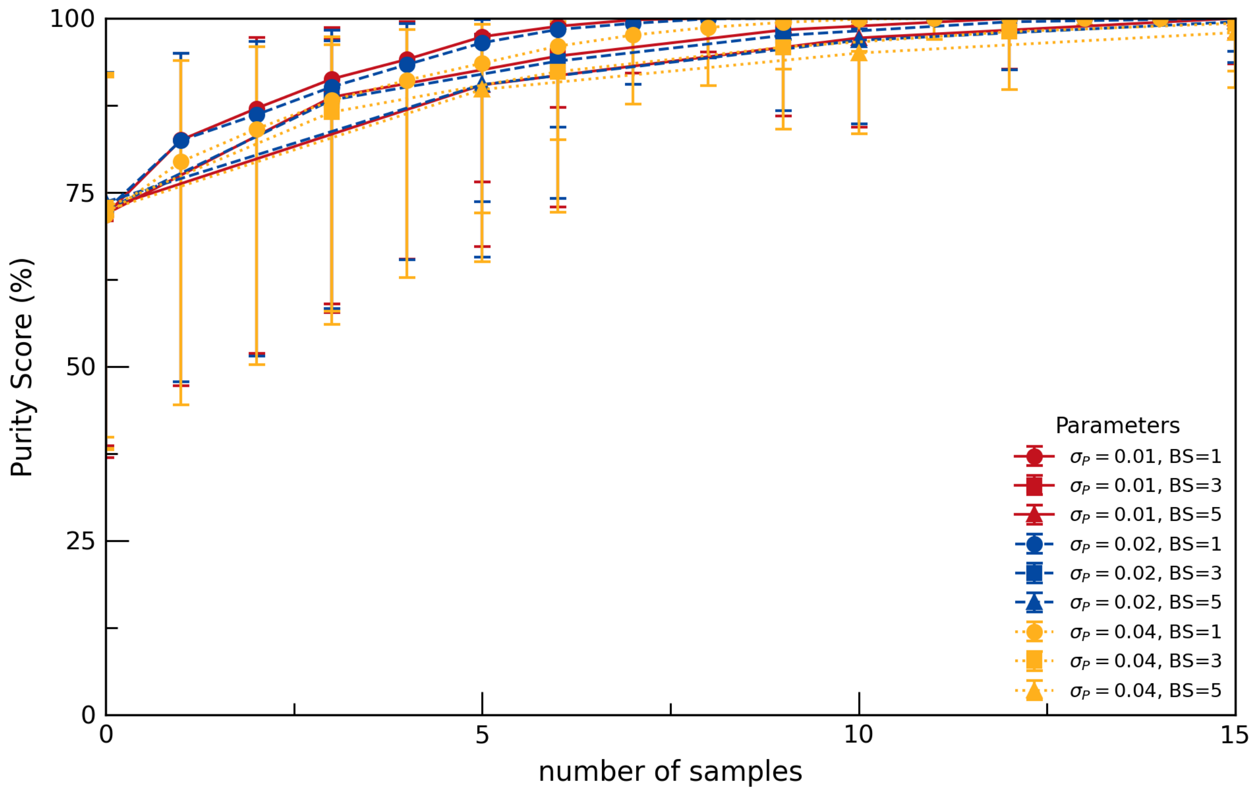

Figure S52: Median Purity Score vs number of samples for the $Mg_5B_3O_9F$ phase and an experimental error of 2 wt% (σ_E=0.02). Results are shown for all combinations of PICIP error (σ_P) and Batch size (BS). The error bars show the 16th and 84th percentiles respectively.

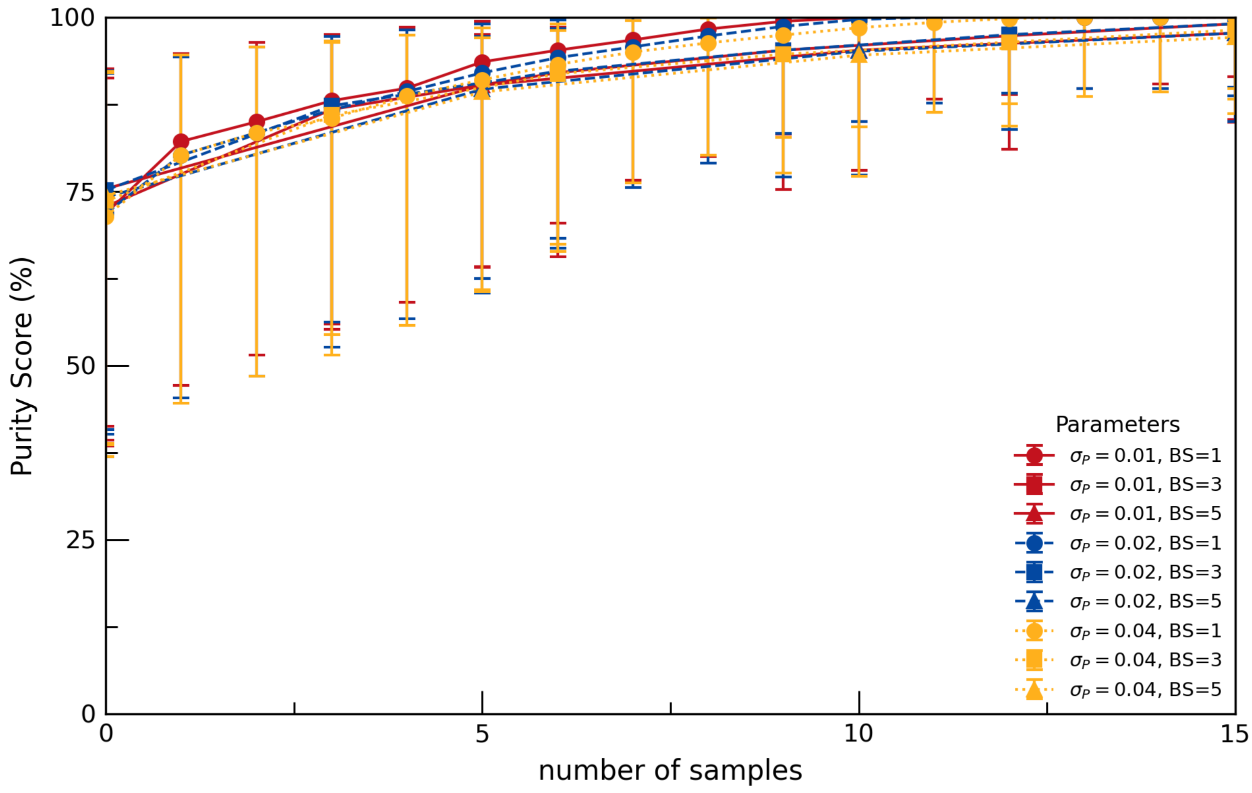

Figure S53: Median Purity Score vs number of samples for the $Mg_5B_3O_9F$ phase and an experimental error of 5 wt% (σ_E=0.05). Results are shown for all combinations of PICIP error (σ_P) and Batch size (BS). The error bars show the 16th and 84th percentiles respectively.

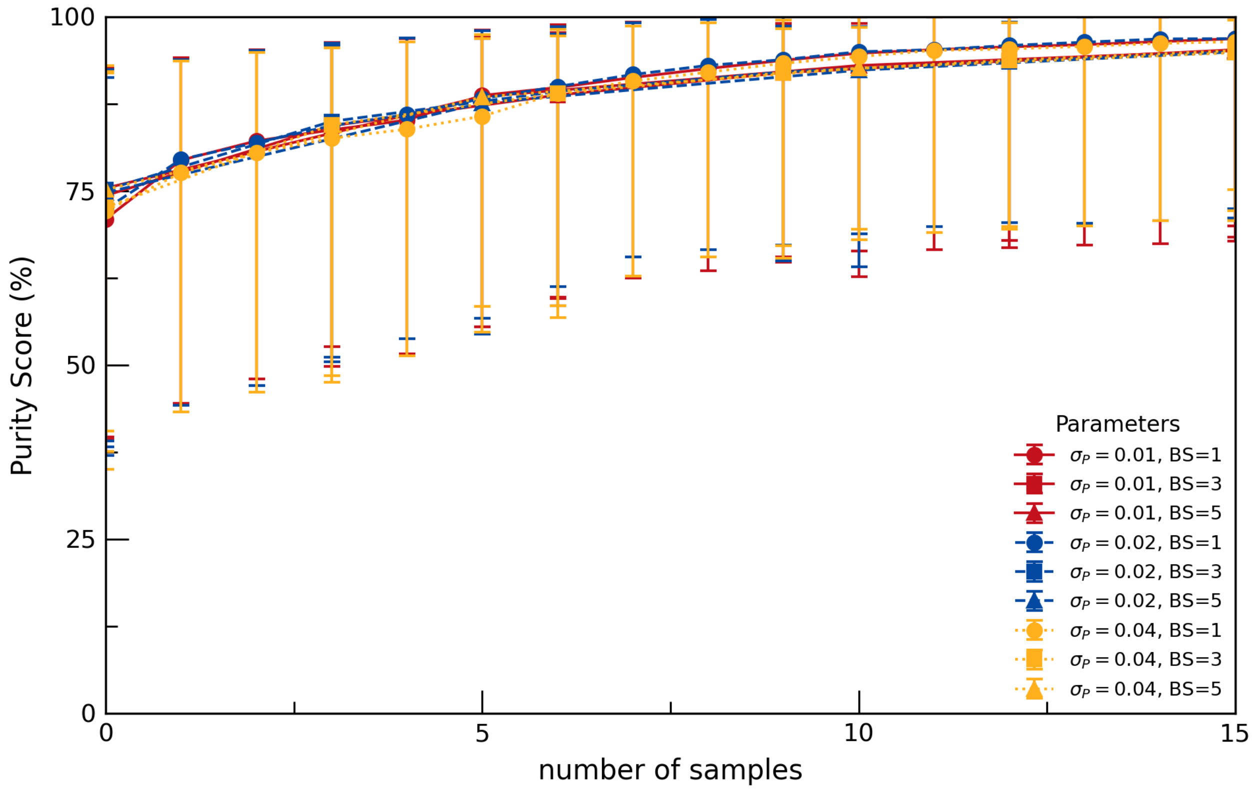

Figure S54: Median Purity Score vs number of samples for the $Mg_5B_3O_9F$ phase and an experimental error of 10 wt% (σ_E=0.1). Results are shown for all combinations of PICIP error (σ_P) and Batch size (BS). The error bars show the 16th and 84th percentiles respectively.

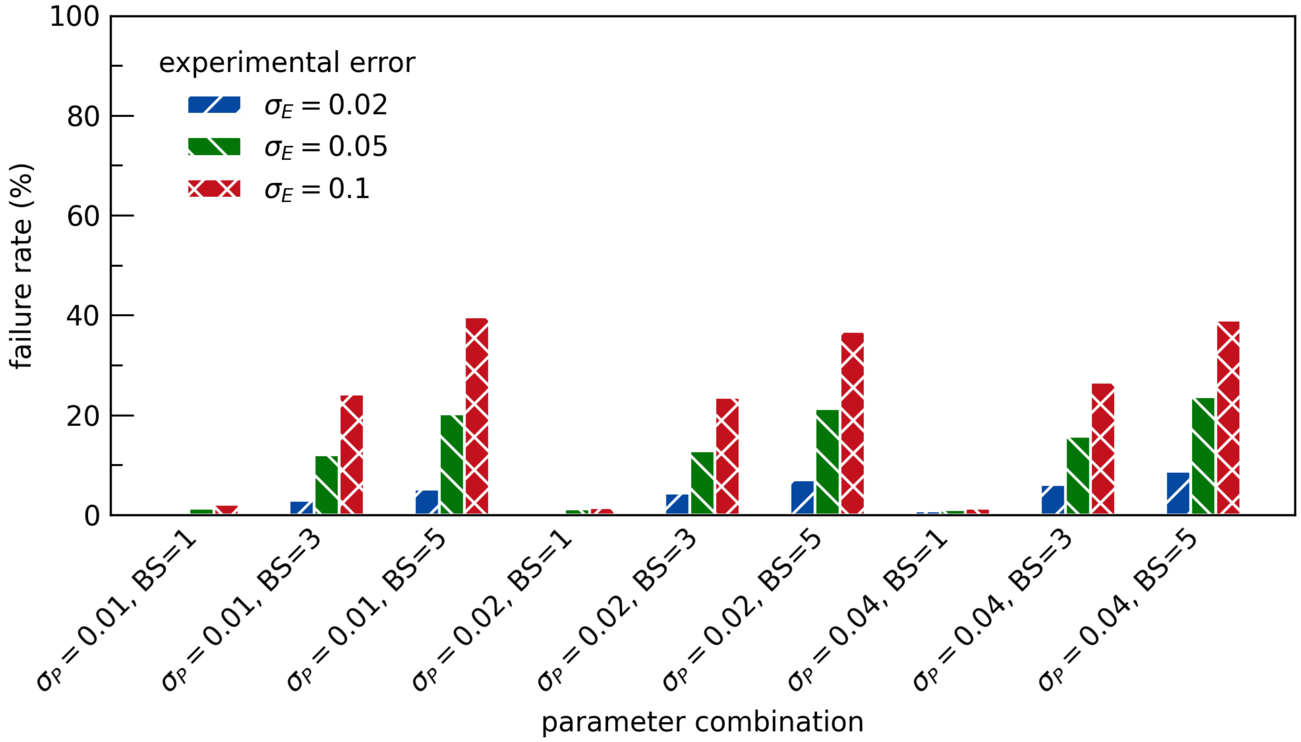

Figure S55: PICIP's failure rate for the $Mg_5B_3O_9F$ phase, for all combinations of PICIP error (σ_P), Experimental error (σ_E), and Batch size (BS)

4 Mathematical representation of phase field

Consider the phase field for a set of m elements. These m different elements are known, and labelled by $i \in \{1, 2, ..., m\}$. Any composition a in this phase field can then be represented as $\mathbf{x} = \sum_{i=1}^{m} x_i \mathbf{e_i}$, where $\{\mathbf{e_i}\}$ is the standard basis for $\mathbb{R}^m$ and x_i is the number of moles of the i'th element.

Due to the condition that compositions with equal ratios of elements are equivalent, the representation for any point in the phase field can be chosen such that the total number of moles is equal to 1. This means all points in the phase field will satisfy the linear equation $\sum_i x_i = 1$. This representation is denoted the standard representation.

The consequence of assuming exactly one formal charge for each element is that the charge neutrality constraint becomes a single linear equation, $\sum_i q_i x_i = 0$, where q_i is the formal charge for the i'th element. All points in the phase field will satisfy this linear equation.

Because all points in the phase field satisfy two independent linear equations, they will belong to a $n = m - 2$ dimensional subspace of $\mathbb{R}^m$. This subspace will be orthogonal to both of the vectors representing the linear constraint equations. It can be spanned by a set of n orthonormal basis vectors denoted $\mathbf{u}_i$, which are found using standard linear algebra methods. This basis, along with a suitable translation vector, may be used to find an affine transformation which maps composition vectors in the m dimensional standard representation to n dimensional vectors.

Let $\mathbf{c}$ be the column vector in the standard representation for a fixed composition in the phase field and let $\mathbf{x}$ be the column vector in the standard representation for some point a in the phase field. The change of basis matrix, $\mathbf{B} \in \mathbb{R}^{nxm}$, is constructed as the matrix with each of its n rows equal to a $\mathbf{u}_i$. Then $\mathbf{x'} = \mathbf{B}(\mathbf{x} - \mathbf{c})$ is the n dimensional column vector for coordinates of a in the constrained representation. Inversely, given $\mathbf{x'}$, the coordinate vector in the constrained representation, the corresponding coordinate vector in the standard representation is given by $\mathbf{x} = \mathbf{B}^{\mathrm{T}}\mathbf{x'} + \mathbf{c}$.

This lower dimensional representation is denoted the constrained representation, $C \subseteq \mathbb{R}^n$.

5 Calculating direction from relative mass fractions

After a composition has been sampled, the result will be a mixture of one or more crystalline phases. The average composition of the mixture will be a weighted average of the composition of the resultant crystalline phases, where the weighting is given by the relative number of moles of each crystalline phase. We assume that the sample is a closed system, and the composition was measured exactly, and so the average composition of the mixture is equal to the sampled composition.

Now consider the situation where one of the crystalline phases is unknown, and the rest are known. Assuming we can determine the exact ratio between the known crystalline phases, we can calculate the average composition of the known crystalline phases. This can be used in the following way to help determine the composition of the unknown crystalline phase.

Let $s \in C$ be a sampled composition. This can be represented as a weighted average of the composition of the unknown crystalline phase, denoted $u \in C$ and the average composition of the known crystalline phases, denoted $k \in C$; $s = (1-\alpha)k + \alpha u, \alpha \in [0, 1]$. By substituting $\lambda + 1 = \frac{1}{\alpha}$ this can be rearranged to get $u = s + \lambda(s - k), \lambda \in \mathbb{R}_+$, i.e. the unknown crystalline phase lies on the line segment parallel to $s - k$, with endpoints at s and the bounds of C. We denote the set of compositions contained in this segment as $T_{s,k} = \{x \in C : x = s + \lambda(s - k), \lambda \in \mathbb{R}_+\}$. To make the notation easier to follow, $T(s, k)$ will be used to denote $T_{s,k}$ from here.

6 Allowing for experimental uncertainty

Recall k is not known, it is estimated from an experimental observation with associated uncertainty, and therefore we introduce the random variable, $\mathrm{K} \sim \mathcal{N}(\mu, \Sigma)$ such that $f_{\mathrm{K}}(a)$ gives the probability density of the composition a being k.

Observe that $T(s, k_1) = T(s, k_2)$ if $k_1 = k_2 + \lambda(s - k_2), \lambda \in \mathbb{R}$. Therefore the probability density of u belonging to $T(s, k)$ is

$$f(T(s, k)) = \int_{[k]} f_{\mathrm{K}}(k) \, \mathrm{d}k$$

where $[k]$ denotes the equivalence class $k_1 \sim k_2 \implies k_1 = k_2 + \lambda(s - k_2), \lambda \in \mathbb{R}$.

To compute $f(T(s, k))$ we use the marginal probability density of K on $T^{\perp}(s, k)$, the orthogonal compliment to $T(s, k)$.

Let $A \in \mathbb{R}^{nxn-1}$ be a matrix which maps from C to $T^{\perp}(s, k)$, which implies that $\forall i \sum_j A_{ij}(s - k)_j = 0$. Denoting $\mathrm{K}^{\perp}(s, k)$ as the marginal of K on $T^{\perp}(s, k)$, we have $\mathrm{K}^{\perp}(s, k) \sim \mathcal{N}(A^T\mu, A^T\Sigma A)$. Then let b be the point of intersection between the $n - 1$ dimensional hyperplane containing μ with normal vector $s - k$, and the line through points s and k. The representation of b in the orthogonal compliment to $s - k$ would be $b^{\perp} = A^T s$. This allows us to compute $f(T(s, k))$ as

$$f(T(s, k)) = f_{\mathrm{K}^{\perp}(s,k)}(b^{\perp}).$$

Finally, the probability density of the u being at the point $p \in T(s,k)$ is simply $f(T(s,k))$ divided by the length of $T(s,k)$. By choosing a set of $T(s,k)$ to cover C with sufficient density, the probability density of u being at any point $p \in C$, denoted $f(p)$, can be estimated.

7 PICIP error

The random variable K is used to estimate k, the (weighted) average composition of the known crystalline phase's, where the weighting is given by the relative mole fractions. These relative mole fractions will have associated experimental errors, arising from the estimation of the relative mass fractions by R-QPA.

These experimental errors will be dependent on a multitude of factors such as the quality of PXRD pattern or the phase field being explored and quantifying them is an important and long standing problem. For example, in [1] identical mixtures containing multiple crystalline phases were sent to several laboratories to find the standard deviation on the estimated mass fractions. These showed variation between different crystalline phases and laboratories, and a high correlation with the amount of phase in a sample. Therefore the construction of K is designed to allow for uncertainties on each individual mass fraction to be provided.

Having this fidelity is important, and will allow for more accurate results. However utilising it may not always be possible; in many situations estimations of the experimental error on individual mass fractions will be unavailable. When this is the case a constant uncertainty may be assumed for each mole fraction. The PICIP error, σ_P, discussed in the main manuscript is exactly this; a constant standard deviation assumed on each of the relative mass fractions.

K is described by the normal distribution $\mathcal{N}(\mu, \Sigma)$ which is constructed as follows. Known crystalline phases are enumerated and labelled by j. The relative mass fraction of the j'th known crystalline phase is w_j with standard deviation σ_{w_j}. The molar mass of the j'th known crystalline phase is M_j. The total mass of the sample is m with standard deviation σ_m.

Then the number of moles of the j'th known crystalline phase, denoted n_j, and their respective standard deviations, denoted σ_{n_j} are

$$n_j = \frac{w_j m}{M_j}, \; \sigma_{n_j} = \frac{\sqrt{(m\sigma_{w_j})^2 + (w_j \sigma_m)^2}}{M_j} \; .$$

Each of the known crystalline phases can be represented by $\mathbf{x}_j \in S$ where S is the standard representation as defined above. The average composition of the known crystalline phases, denoted $\mathbf{k}$, is then

$$\mathbf{k} = \frac{\sum_j n_j \mathbf{x}_j}{\sum_j n_j}$$

. Then with x_{ji} as the i'th element of $\mathbf{x}_j$ the standard deviation on the i'th element of $\mathbf{k}$ is

$$\sigma_{\mathbf{k}_i} = \frac{\sqrt{\sum_j (\sigma_{n_j} x_{ji})^2}}{\sum_j n_j}$$

These standard deviations are then taken as the diagonal of a covariance matrix for a multivariate normal distribution - $\mathcal{N}(\boldsymbol{\mu_S}, \mathbf{\Sigma_S})$ with $\boldsymbol{\mu}_S = \mathbf{k}$ and $\mathbf{\Sigma}_S = \text{diag}([\sigma^2_{\mathbf{k}_1}, ..., \sigma^2_{\mathbf{k}_n}])$.

The final step is to project this normal distribution onto C, to result in our model for the random variable K. The multivariate normal distribution in the constrained composition space is $\mathcal{N}(\boldsymbol{\mu}, \mathbf{\Sigma})$ with $\boldsymbol{\mu} = \mathbf{B}(\boldsymbol{\mu} - \mathbf{c})$ and $\mathbf{\Sigma} = \mathbf{B}\mathbf{\Sigma}\mathbf{B}^\mathbf{T}$, where $\mathbf{c}$ is the previously mentioned fixed point in phase field.

8 Edge cases in model simulation

If there are only trace amounts of the unknown phase, then its presence would not be identified experimentally. For this reason if the true mass fraction of the unknown crystalline phase is less than a user specified threshold (*minimum u*), then it is assumed to not be present.

Compositions suggested by PICIP may fall into regions of the phase diagram where the unknown phase will not form. These regions are denoted void simplicis. Normally these sample points are then completely ignored. However it is possible for the algorithm to get stuck in a loop where the probability density falls almost entirely over these void regions. It therefore repeatedly samples in these regions, and the probability density never gets updated. To prevent this, the following logic is implemented; if an entire batch falls within one or more void simplicis, then these regions are removed from the discretised grid and the probability density is renormalised. This is deemed reasonable as if a sampled composition yielded only known phases in an experiment, then the region of the phase field bounded by those phases would not be considered for further sampling.

9 Discretisation schemes

The hyperparameter *number of lines* sets the number of $T(s,k)$. Ensuring that these are evenly spread out is equivalent to finding a uniform distribution of points on the surface of a hyper-sphere. In 2 dimensions this is trivial, in 3 dimensions the mapping of the Fibonacci lattice to the surface of the sphere yields a nearly uniform result [2], and in higher dimensions samples are drawn from the uniform distribution proposed in [3].

The hyperparameter *points per line* defines the number of points for each $T(s,k)$. Together with *number of lines* this defines the unique discretisation scheme of the phase field for each sampled point.

In order to combine the probability densities from multiple sampled points it is necessary to have a constant discretisation scheme. The lattice formed by basis vectors found in the construction of the constrained representation is used for this. Each vector is scaled by $1/gridsize$ where *gridsize* is another hyperparameter.

10 Choice of hyperparameters

The total mass of the sample is set as 1g with standard deviation 0.02g.

For the hyperparameters controlling the resolution of the discretisation schemes there is a trade off between modelling accuracy and computational time. Values of 100, 50 and 100 for *number of lines, points per line and gridsize* respectively were found to keep the computational time acceptably small. *minimum u*, the threshold yield below which its assumed no presence of the unknown crystalline phase was detected was chosen as 10% as suggested by experimental colleagues.

References

[1] L. Leon-Reina, A. De la Torre, J. Porras-Vazquez, M. Cruz, L. Ordonez, X. Alcobé, F. Gispert Guirado, A. Larrañaga-Varga, M. Paul, T. Fuellmann, R. Schmidt, and M. Aranda, "Round robin on rietveld quantitative phase analysis of portland cements," *Journal of Applied Crystallography - J APPL CRYST*, vol. 42, pp. 906–916, 10 2009.

[2] B. Keinert, M. Innmann, M. Sänger, and M. Stamminger, "Spherical fibonacci mapping," *ACM Trans. Graph.*, vol. 34, nov 2015.

[3] M. E. Muller, "A note on a method for generating points uniformly on n-dimensional spheres," *Commun. ACM*, vol. 2, p. 19–20, apr 1959.
